# From *Salvia dumetorum* Hydroethanolic Extract to Tablet Formulation: Integrated Phytochemical, Biological, and Preclinical Safety Evaluation

**DOI:** 10.3390/molecules31213802

**Published:** 2026-10-22

**Authors:** Yana K. Levaya, Karakoz Zh. Badekova, Gulnissa K. Kurmantayeva, Mussa E. Zholdasbayev, Gülmira Özek, Temel Özek, Izabela Korona-Glowniak, Wirginia Kukula-Koch, Elina Smagulova, Gayane A. Atazhanova

**Affiliations:** 1 School of Pharmacy, Karaganda Medical University, Gogol Street, 40, Karaganda 100017, Kazakhstan; badekova@qmu.kz (K.Z.B.); kurmantaeva@qmu.kz (G.K.K.); zholdasbayev.mussa.95@gmail.com (M.E.Z.); elinasmagulova0509@gmail.com (E.S.); g-atazhanova@mail.ru (G.A.A.); 2 Department of Pharmacognosy, Faculty of Pharmacy, Anadolu University, 26470 Eskisehir, Türkiye; gozek@anadolu.edu.tr (G.Ö.); tozek@anadolu.edu.tr (T.Ö.); 3 Department of Pharmaceutical Microbiology, Medical University of Lublin, 1 Chodzki Street, 20-093 Lublin, Poland; izabela.korona-glowniak@umlub.edu.pl; 4 Department of Pharmacognosy with Medical Plants Garden, Medical University of Lublin, 1 Chodzki Str., 20-093 Lublin, Poland; virginia.kukula@gmail.com

**Keywords:** *Salvia dumetorum*, phytochemical profiling, HPLC-QTOF-ESI-MS/MS, antioxidant activity, α-amylase inhibition, antimicrobial activity, preclinical safety

## Abstract

Background/Objectives: *Salvia dumetorum* Andrz. ex Besser is a relatively understudied medicinal plant with promising biological potential, but its phytochemical characteristics and the safety of preparations developed from its extract remain insufficiently investigated. This study aimed to characterize the phytochemical composition and biological properties of *S. dumetorum* extract and to evaluate the preclinical safety of tablets developed on its basis. Methods: The hydroethanolic extract was obtained by microwave-assisted extraction and characterized by HPLC-ESI-QTOF-MS/MS, while total phenolic content (TPC), total flavonoid contents (TFC), antioxidant, α-amylase inhibitory, and antimicrobial activities were determined using spectrophotometric and microbiological assays. Results: The extract TPC and TFC were 40.15 ± 2.25 mg GAE/g_extrt_ and 40.55 ± 5.56 mg QE/g_extr_, respectively. HPLC-QTOF-ESI-MS/MS profiling revealed a diverse spectrum of phenolic, flavonoid, diterpenoid, and organic acid constituents. The extract demonstrated strong antioxidant activity, with trolox equivalent antioxidant capacity (TEAC), total antioxidant capacity (TAC), and ferric reducing antioxidant power (FRAP) values of 1.88 ± 0.07 mM, 250.43 ± 12.08 mg AsAE/g_extr_, and 427.91 ± 9.74 mg TE/g_extr_, respectively, and a DPPH IC_50_ of 0.03 ± 0.00 mg/mL. α-Amylase inhibitory activity was 5.63 ± 0.51 mg AcrE/g_extr_, while antimicrobial activity against the tested microorganisms was observed at MICs of 1.25–10 mg/mL. The resulting tablets retained measurable antioxidant and α-amylase inhibitory activities, although their activity profiles differed from those of the original extract. Comprehensive in vivo evaluation demonstrated no treatment-related adverse effects following repeated administration, including no significant effects on immune parameters, female reproductive function, embryofetal development, skeletal ossification, or sensitization. Conclusions: *S. dumetorum* extract demonstrated diverse biological activities, while the tablet formulation showed a favorable preclinical safety profile. These findings extend the available acute toxicity data and support the further pharmaceutical development of this tablet formulation.

## 1. Introduction

Medicinal plants remain an important source of biologically active compounds and continue to attract considerable interest as candidates for the development of pharmaceutical products. Species of the genus *Salvia* L. (Lamiaceae) are particularly notable because of their diverse phytochemical composition and a broad spectrum of reported biological activities, including antioxidant, antimicrobial, anti-inflammatory, and other pharmacological effects [1,2]. However, the transition from a biologically active plant extract to a medicinal product requires not only demonstration of pharmacological potential but also systematic characterization of its safety profile. This is especially important for botanical preparations, which contain complex mixtures of primary and secondary metabolites whose biological effects may depend on the plant species, extraction procedure, extract composition, dose, route of administration, and pharmaceutical formulation [3,4].

*Salvia dumetorum* Andrz. ex Besser is a relatively understudied species of the genus *Salvia* that is native to Central Asia and occurs naturally in Kazakhstan, including Central Kazakhstan, where it represents a promising and locally available source of biologically active phytochemicals [5]. Despite its distribution and abundance in the region, its phytochemical composition, biological properties, and especially its safety profile remain insufficiently characterized. Although phytochemical investigations of *S. dumetorum* and related *Salvia* species have demonstrated the presence of phenolic compounds, flavonoids, and other secondary metabolites associated with potentially beneficial biological activities, the toxicological characterization of this species remains considerably less developed than its phytochemical and pharmacological characterization [1,2]. In particular, safety data concerning the final oral dosage form based on *S. dumetorum* extract remain limited. This distinction is important because the safety characteristics of a crude plant extract cannot necessarily be extrapolated directly to a finished pharmaceutical formulation, in which extract concentration, excipients, manufacturing processes, and the resulting physicochemical properties may influence the biological response [4,5].

The development of a tablet formulation based on *S. dumetorum* therefore requires a stepwise preclinical safety evaluation addressing both systemic and organ-specific effects as well as safety endpoints that cannot be assessed by acute toxicity testing alone. Previous experimental work has demonstrated the feasibility of pharmaceutical development and standardization of *S. dumetorum*-based tablets, supporting their further investigation as a potential phytopharmaceutical preparation [6]. In addition, the acute oral toxicity of the tablet formulation has been experimentally characterized [7]. Following a single oral administration at doses up to 2000 mg/kg, no mortality or treatment-related statistically significant changes in body weight, hematological parameters, relative organ weights, or major histopathological abnormalities were observed, supporting a favorable acute oral safety profile under the tested conditions [8]. These findings provide an essential foundation for further preclinical evaluation but do not establish the safety of repeated exposure or exclude potential effects on reproductive function, embryofetal development, immune responses, or sensitization.

This distinction is particularly relevant for a preparation intended for repeated administration. Acute toxicity studies primarily characterize the response to a single exposure and therefore cannot adequately address delayed or cumulative effects associated with repeated dosing [8]. Similarly, the absence of acute systemic toxicity does not provide sufficient evidence regarding female reproductive function, estrous cyclicity, fertility, embryofetal development during organogenesis, skeletal development, or the potential of a formulation to induce local irritation or immunological sensitization [9]. Although toxicological studies have been reported for several other *Salvia* species, including investigations of acute and repeated-dose exposure, these findings cannot be directly generalized to *S. dumetorum* because interspecific differences in phytochemical composition may result in substantially different biological and toxicological profiles. For example, oral acute and subacute toxicity has been investigated for *S. przewalskii* Maxim., demonstrating that toxicity characteristics can vary with dose, route, and exposure duration [10]. Safety assessment of *Salvia* preparations is also particularly relevant because some species contain biologically active diterpenoids and volatile constituents for which dose-dependent toxicological effects have been documented; thujone-containing sage preparations, for example, have received particular toxicological attention because of their potential neurotoxicity and the importance of exposure level in risk assessment [11,12].

Consequently, an important gap remains in the preclinical characterization of *S. dumetorum* tablets: a comprehensive evaluation integrating repeated-dose systemic safety with reproductive, embryofetal, immunological, and local tolerance or sensitization endpoints has not been adequately documented. Importantly, the available toxicological literature for *Salvia* is dominated by studies of crude extracts, essential oils, or acute and short-term exposure, whereas comprehensive safety characterization of a defined oral solid dosage form remains limited. In the case of *S. dumetorum*, evidence integrating repeated-dose systemic toxicity with reproductive, embryofetal, immunological, and local sensitization endpoints is particularly scarce, leaving an important translational gap between phytochemical and pharmacological characterization and evaluation of the finished pharmaceutical product.

Therefore, the present study was designed to provide an integrated preclinical safety assessment of *S. dumetorum* tablets following repeated administration. The investigation evaluated systemic tolerability and immunotoxicity, female reproductive function, including sexual behavior, estrous cyclicity and fertility, embryofetal development during the organogenesis period, skeletal ossification and developmental abnormalities, as well as local irritation and sensitizing potential. By integrating these complementary safety endpoints within a single assessment of the final tablet formulation, the findings obtained in this work extend the available acute oral toxicity evidence and address important gaps in the preclinical characterization of *S. dumetorum* tablets. This comprehensive approach provides a broader toxicological basis for evaluating the safety and further pharmaceutical development of this plant-based dosage form.

## 2. Results

The results of the study provide a comprehensive characterization of the phytochemical composition and biological properties of the *S. dumetorum* extract and the developed tablet formulation. The extract was characterized by HPLC-ESI-QTOF-MS/MS, followed by evaluation of its total phenolic and flavonoid contents, antioxidant, α-amylase inhibitory, and antimicrobial activities. The tablet formulation was subsequently evaluated for its phenolic composition and selected biological activities, including antioxidant and α-amylase inhibitory effects. In addition, a series of in vivo studies were performed to characterize the pharmacological and safety profile of the tablets, including their anti-inflammatory activity, repeated-dose toxicity, immunotoxicity, allergenic properties, and effects on reproductive function and embryofetal development.

### 2.1. Phytochemical Characterization and Biological Activity of the Extract and Tablets

#### 2.1.1. HPLC-QTOF-ESI-MS/MS Profiling of *S. dumetorum* Extract

The phytochemical profile of the *S. dumetorum* extract was characterized using HPLC-QTOF-ESI-MS/MS in the negative ionization mode (Figure 1). A total of 39 compounds were tentatively identified in the extract (Table 1). The identified constituents represented several major classes of secondary metabolites, including phenolic acids, phenylpropanoids, flavonoids and their glycosides, diterpenoids, and fatty acids, as well as several organic acids. Compounds were identified based on the combined evidence from accurate mass, molecular formula, retention behavior, and MS/MS fragmentation (Figures S1–S39), with identification confidence levels reported according to the Metabolomics Standards Initiative (MSI) framework.

**Figure 2:**
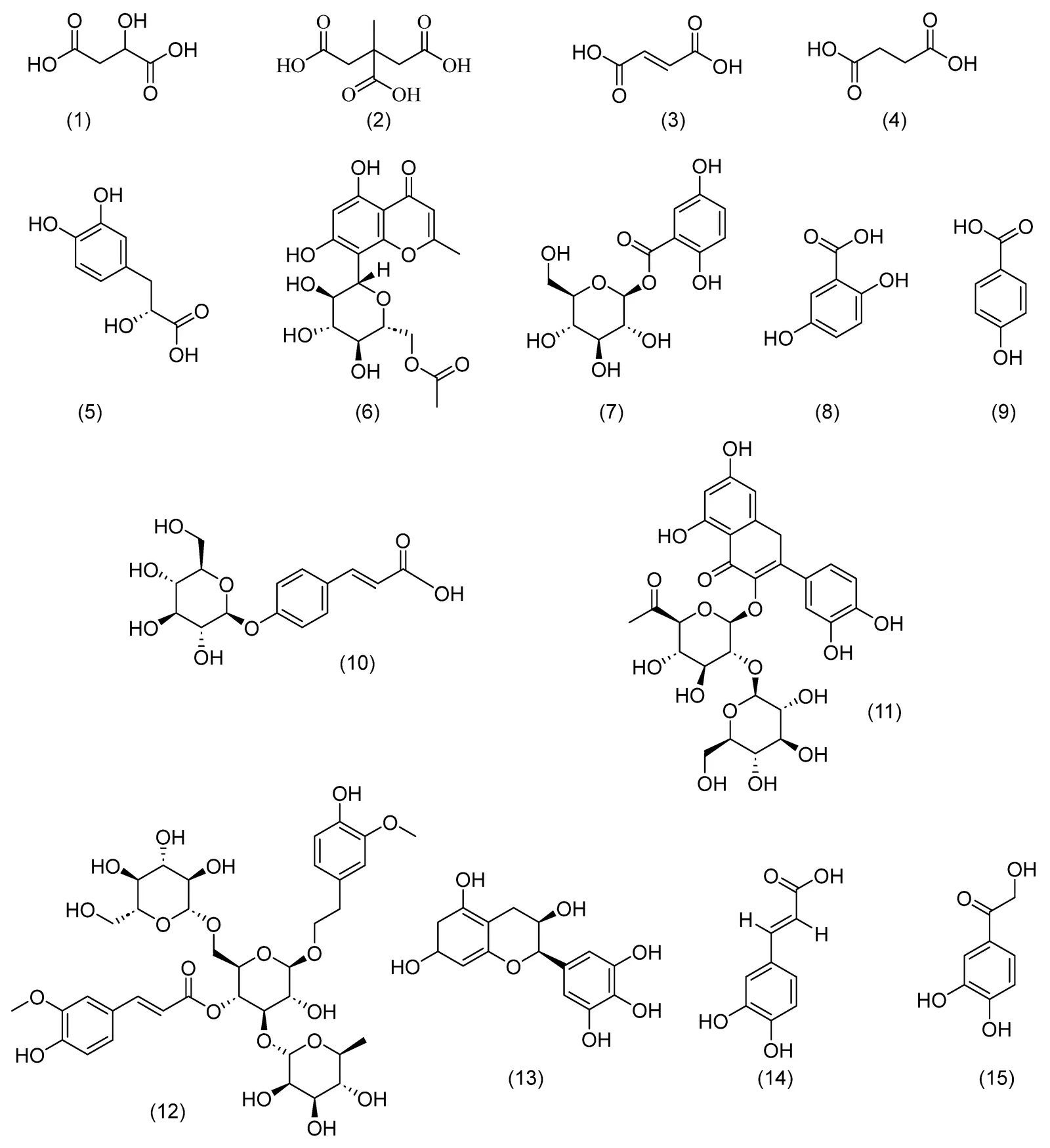

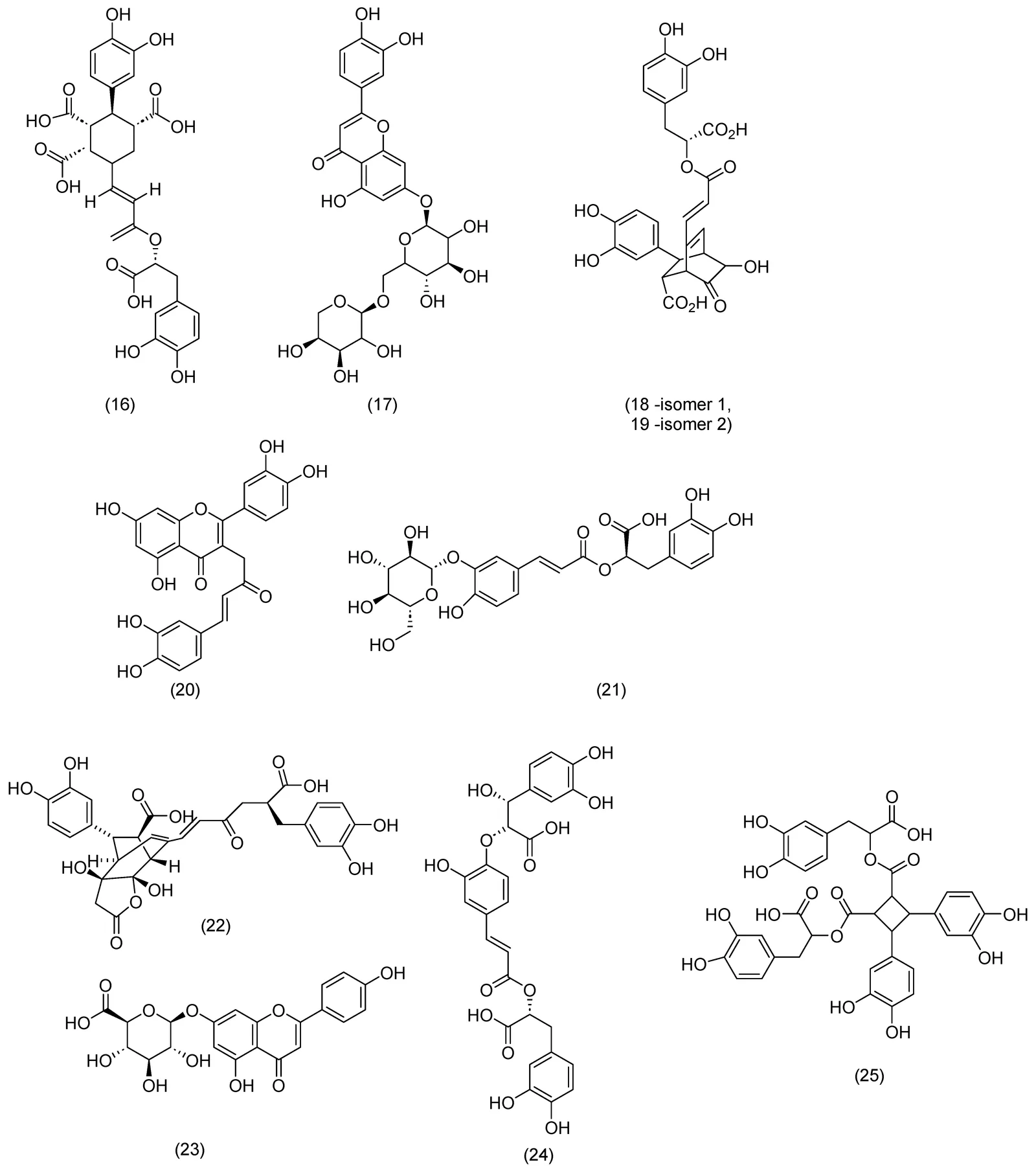

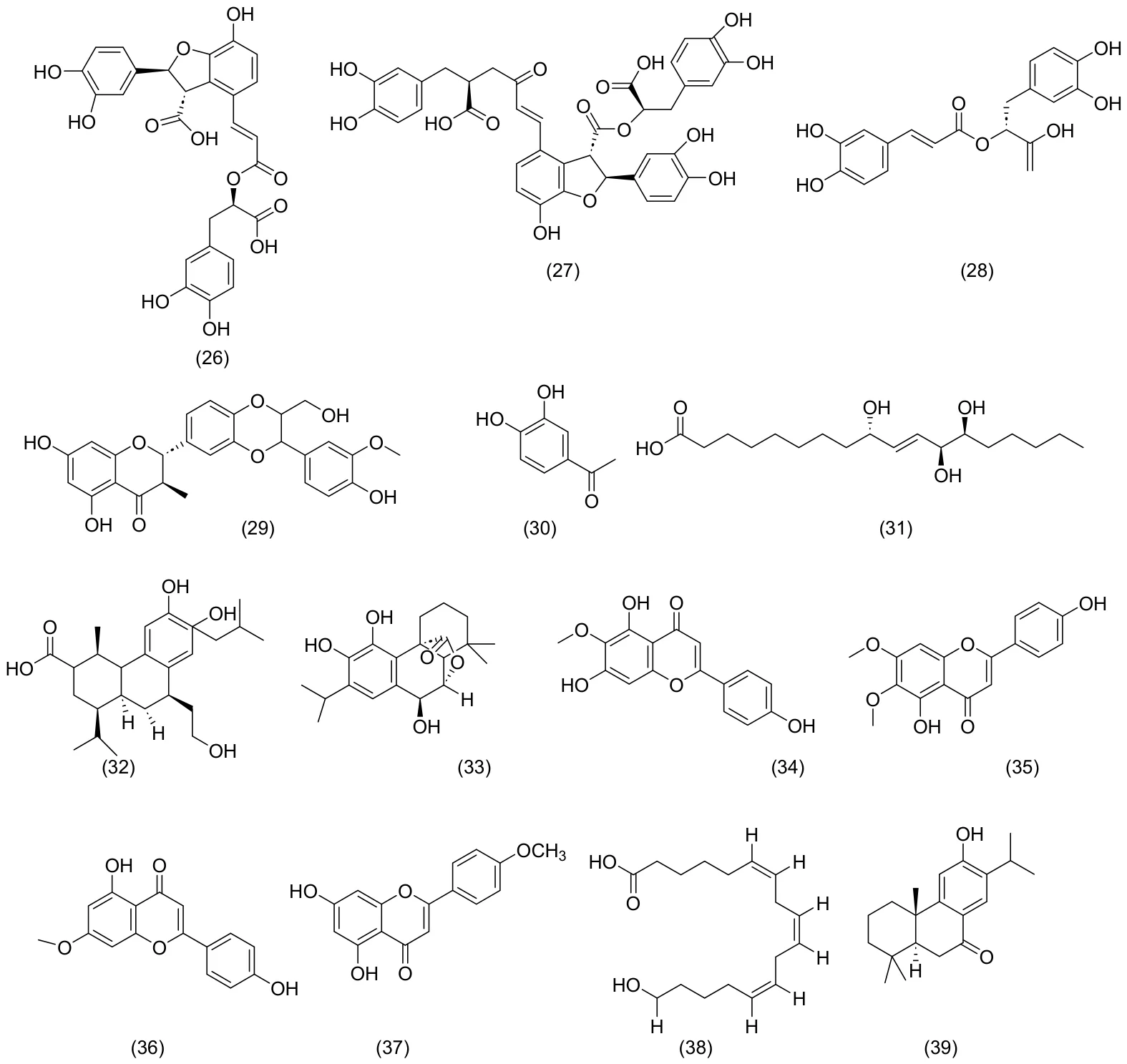
Identified constituents in *S. dumetorum* hydroethanolic extract.

The chemical profile was predominantly characterized by phenolic compounds, particularly phenylpropanoid derivatives and phenolic acids. Among the tentatively identified constituents, rosmarinic acid, lithospermic acids A and B, sagerinic acid, yunnaneic acids D–F, danshensu, caffeic acid, and p-coumaric acid glucoside were detected, indicating a diverse profile of phenolic metabolites characteristic of *Salvia* species [2] (Figure 2). This phenolic acid matrix was further extended by the presence of protocatechuic acid, gentisic acid, hydroxybenzoic acid, protocatechuic aldehyde, and gentisoyl glucoside, while primary metabolic pathways were represented by aliphatic organic acids including malic, citric, fumaric, and succinic acids. Additionally, a diverse flavonoid profile was observed, comprising epigallocatechin, castillicetin, quercetin glucosyl-glucuronide, hispidulin, cirsimaritin, genkwanin, acacetin, apigenin glucuronide, and a luteolin rutinoside derivative, along with the flavonolignan silybin. The lipophilic terpenoid fraction was characterized by phenolic diterpenes, namely rosmanol, hydroxycarnosic acid, and sugiol, accompanied by oxylipins such as pinellic and hydroxylinoleic acids. Finally, the specialized chemical profile was complemented by glycosidic constituents, specifically the phenylethanoid glycoside cistanoside E and the chromone glycoside acetylisobiflorin.

Several flavonoid derivatives, including epigallocatechin, luteolin rutinoside derivative, apigenin glucuronide, hispidulin, cirsimaritin, genkwanin, and acacetin, were also detected. In addition, diterpenoid constituents such as carnosic acid-related compounds, hydroxycarnosic acid, rosmanol, and sugiol were tentatively assigned. Organic acids, including malic, citric, fumaric, and succinic acids, were detected predominantly among the early-eluting compounds.

#### 2.1.2. Total Phenolic Content and Total Flavonoid Content of the Extract and Tablets

TPC and TFC of the *S. dumetorum* extract and tablets were determined spectrophotometrically using gallic acid and quercetin as reference standards, respectively. The extract showed a TPC of 40.15 ± 2.25 mg GAE/g_extr_ and a TFC of 40.55 ± 5.56 mg QE/g_extr_. The tablets retained a measurable phenolic content of 38.91 ± 9.63 mg GAE/g_tablet_, while flavonoids were not detected under the applied experimental conditions (Table 2).

#### 2.1.3. Antioxidant Activity of the Extract and Tablets

The antioxidant activity of the *S. dumetorum* extract and extract-based tablets was evaluated using four complementary spectrophotometric in vitro assays: TEAC, TAC, FRAP, and DPPH radical-scavenging activity (Table 3). The extract exhibited a TEAC value of 1.88 ± 0.07 mM, a TAC of 250.43 ± 12.08 mg AsAE/g_extr_, and a FRAP value of 427.91 ± 9.74 mg TE/g_extr_. Its DPPH radical-scavenging activity corresponded to an IC_50_ value of 0.03 ± 0.00 mg/mL. The tablets demonstrated TEAC, TAC, and FRAP values of 0.16 ± 0.00 mM, 69.57 ± 6.85 mg AsAE/g_tablet_, and 55.83 ± 1.92 mg TE/g_tablet_, respectively, while their DPPH IC_50_ value was >1.0 mg/mL. Overall, the tablets exhibited lower antioxidant activity than the extract across the tested assays.

**Figure 3:**
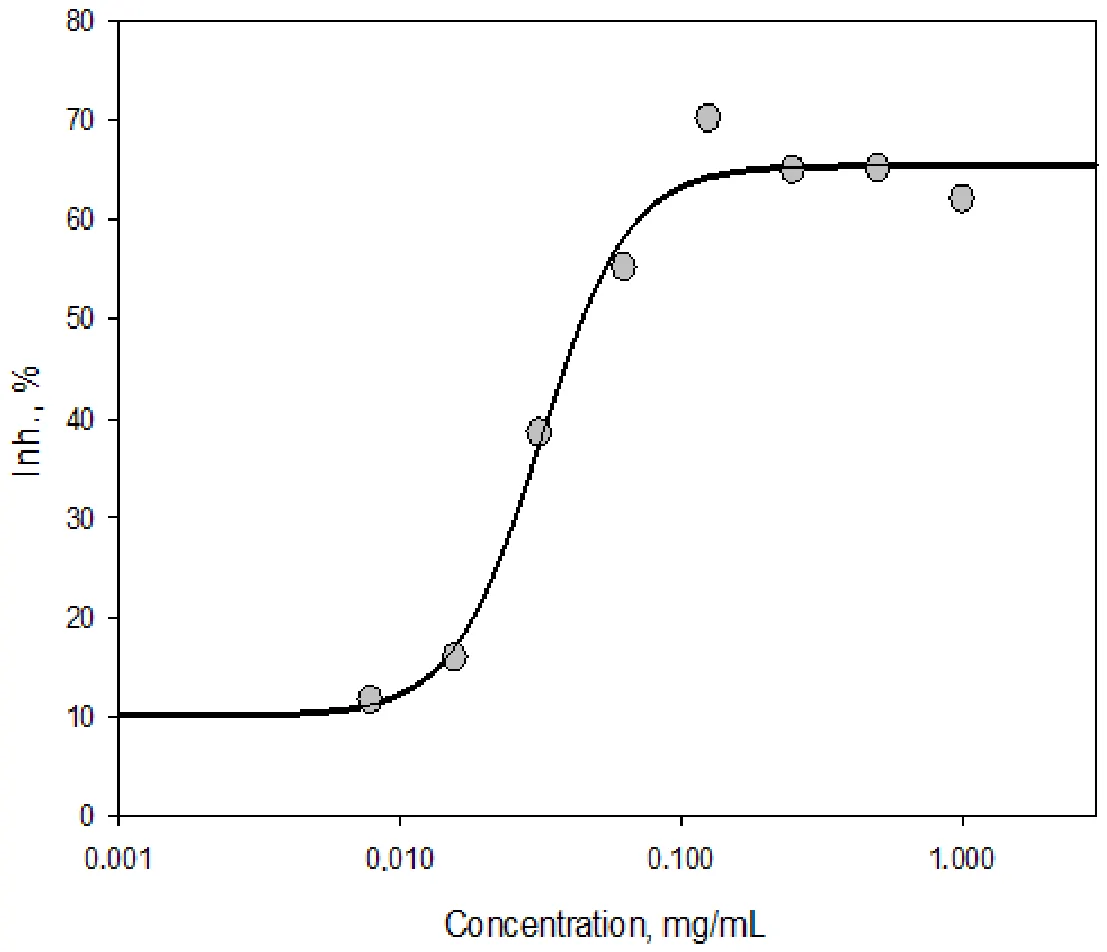
Dose–response curves used to determine the IC_50_ value of the *S. dumetorum* extract in the DPPH radical scavenging assay.

In the DPPH assay, the extract exhibited a pronounced radical-scavenging activity, with an IC_50_ value of 0.03 ± 0.00 mg/mL. The dose–response relationship obtained in the DPPH assay is shown in Figure 3.

#### 2.1.4. α-Amylase Inhibitory Activity of the Extract and Tablets

The inhibitory activity of the *S. dumetorum* extract against α-amylase was evaluated as an in vitro indicator of its potential to modulate carbohydrate-digesting enzyme activity. The extract demonstrated α-amylase inhibitory activity corresponding to 5.63 ± 0.51 mg acarbose equivalents (AcrE)/g_extr_ (Table 4).

#### 2.1.5. Antimicrobial Activity of *S. dumetorum* Extract

The *S. dumetorum* extract demonstrated antimicrobial activity against all tested microorganisms, with MIC values ranging from 1.25 to 10 mg/mL and MBC/MFC values ranging from 1.25 to 10 mg/mL (Table 5).

Among the Gram-positive bacteria, the lowest MIC (1.25 mg/mL) was recorded for *S. aureus* (both strains), *S. epidermidis*, and *M. luteus*, whereas *E. faecalis* exhibited the highest MIC (5 mg/mL). Among Gram-negative bacteria, MIC values ranged from 2.5 mg/mL for *K. pneumoniae* to 10 mg/mL for *S.* Typhimurium, *E. coli*, and *P. aeruginosa*. For the tested yeasts, *C. glabrata* showed the lowest MIC (1.25 mg/mL), followed by *C. albicans* (2.5 mg/mL) and *C. parapsilosis* (5 mg/mL).

### 2.2. Standardization of the S. dumetorum Extract and Quality Evaluation of the Tablet Formulation

#### 2.2.1. Standardization of the *S. dumetorum* Extract

To support the transition from the plant extract to the finished tablet formulation, a set of standardization parameters was established for *S. dumetorum* extract. The proposed standardization profile comprised organoleptic characteristics, solubility, identification of characteristic phenolic compounds, loss on drying, heavy metal content, microbiological purity, and quantitative determination of rosmarinic acid. The complete set of standardization parameters and acceptance criteria is presented in Table 6.

#### 2.2.2. Quality Evaluation of the Tablet Formulation

The quality of the tablet formulation containing the dry extract was evaluated in terms of appearance, mass uniformity, disintegration time, dissolution, crushing strength, and friability. The results are presented in Table 7.

In addition, HPLC-UV analysis confirmed the presence of rosmarinic acid in the tablets containing the *S. dumetorum* extract. Quantification using the external standard method showed that rosmarinic acid accounted for 0.41% of the total tablet mass.

### 2.3. Preclinical Safety Evaluation of Tablets

The safety profile of the developed *S. dumetorum* tablets was evaluated in a series of complementary in vivo studies. The experimental program included assessment of anti-inflammatory activity, repeated-dose toxicity, allergenic and immunomodulatory properties. The anti-inflammatory potential was assessed using the carrageenan-induced paw edema model, whereas the safety studies focused on clinical, physiological, immunological, and reproductive endpoints following oral administration. Together, these studies provided a comprehensive preliminary characterization of the efficacy and tolerability of the developed tablet formulation.

#### 2.3.1. Anti-Inflammatory Activity

Anti-inflammatory activity of the *S. dumetorum* extract-based tablets was evaluated using carrageenan-induced inflammation model at three doses: 100, 250 and 500 mg/kg body weights referring to the equivalent amounts of *S. dumetorum* extract in laboratory mice with comparison drugs, presented in Table 8. Values are presented as mean ± SEM. No statistically significant differences were observed between the treated groups and the intact and vehicle-control groups (*p* > 0.05). Food and water consumption were normalized to 100 g body weight.

The data in Table 8 demonstrate that tablets containing dry extract of steppe sage exhibited a dose-dependent, statistically significant anti-inflammatory effect (*p* < 0.05) in the carrageenan-induced inflammation model, as evidenced by a reduction in paw volume compared to the control group at 3, 12, and 24 h. Notably, the most pronounced anti-inflammatory activity was observed 3 h after inflammation induction at doses exceeding 250 mg/kg body weight referring to the equivalent amounts of *S. dumetorum* extract; these effects were comparable to those of the reference drug diclofenac sodium and superior to the anti-inflammatory effect of the herbal preparation “Rotokan.”

#### 2.3.2. Repeated-Dose Toxicity Test

Repeated intragastric administration of *S. dumetorum* tablets at doses of 100, 250, and 1000 mg/kg body weight referring to the equivalent amounts of *S. dumetorum* extract for three weeks did not result in treatment-related changes in the general condition or locomotor activity of mice. Throughout the treatment period, no abnormal clinical signs were observed. Following the three-week recovery period, the general condition and spontaneous activity of treated animals remained comparable to those of the control groups.

Body weight increased progressively in all experimental groups during the treatment period (Table 9). No statistically significant differences in body weight gain were observed between mice receiving *S. dumetorum* tablets and the intact or vehicle-control groups. These findings indicate that repeated administration of the tablets did not adversely affect normal body weight gain. Food and water consumption also remained comparable among the experimental and control groups during both the treatment and recovery periods, with no statistically significant differences observed.

Behavioral activity assessed using the open-field test is presented in Table 10. During the treatment period, horizontal, vertical, and exploratory activity, as well as grooming behavior, remained comparable with control values in male mice. Female mice showed a slight tendency toward increased horizontal locomotor and exploratory activity; however, these changes were not statistically significant. Following the recovery period, no significant differences in behavioral parameters were observed between treated and control animals of either sex.

Hematological analysis demonstrated no significant treatment-related changes in peripheral blood parameters (Table 11). Erythrocyte counts, hemoglobin concentrations, and erythrocyte color index remained within physiological ranges. In male mice, slight decreases in erythrocyte counts were observed at doses of 100 and 250 mg/kg body weight referring to the equivalent amounts of *S. dumetorum* extract (8.3% and 6.0%, respectively); however, these changes were not statistically significant and remained within physiological variation. After the recovery period, the evaluated hematological parameters were comparable with those of both the vehicle-control and intact-control groups.

Serum biochemical parameters measured after three weeks of treatment were also comparable between treated and control animals (Table 12). Repeated administration of *S. dumetorum* tablets at doses of 100, 250, and 1000 mg/kg body weight referring to the equivalent amounts of *S. dumetorum* extract did not result in statistically significant changes in plasma biochemical parameters in either male or female mice compared with the respective control groups (*p* > 0.05). The absence of treatment-related changes in creatinine, urea, alkaline phosphatase, ALT, and AST suggested no detectable adverse effects on renal and hepatic biochemical markers. These parameters remained comparable to control values after the three-week recovery period. Following the recovery period, creatinine, urea, alkaline phosphatase, cholesterol, and transaminase activities remained comparable with control values, indicating no evident treatment-related impairment of hepatic, renal, or metabolic function.

Gross morphological examination of the internal organs revealed no visible pathological changes in animals receiving *S. dumetorum* tablets. Furthermore, organ weight coefficients did not differ significantly from those of the corresponding control groups (Table 13 and Table S3). Comparable findings were observed after the three-week recovery period, with no treatment-related macroscopic abnormalities detected.

Overall, repeated intragastric administration of *S. dumetorum* tablets at doses of 100, 250, and 1000 mg/kg for three weeks did not produce significant adverse effects on clinical condition, body weight, food and water consumption, behavioral activity, cardiac electrophysiology, hematological or biochemical parameters, renal function, or gross organ morphology. The absence of persistent changes after the recovery period further supports the favorable tolerability of the tested tablet formulation under the experimental conditions.

#### 2.3.3. Reproductive and Embryofetal Toxicity

The effects of *S. dumetorum* tablets on female sexual behavior, fertility, and embryo–fetal development were evaluated (Table 14) following intragastric administration at a dose of 250 mg/kg body weight referring to the equivalent amounts of *S. dumetorum* extract. No statistically significant differences were observed between the treated and control groups in sexual behavior parameters, including latency period, sexual activity, emotional approaches to the male, and lordosis responses (*p* > 0.05).

The pregnancy index was numerically higher in the treated group (75.0%) than in both control groups (64.3%); however, this difference was not statistically significant. No statistically significant differences were observed in ovarian relative mass, the numbers of corpora lutea and fetuses, or the rates of resorptions, preimplantation loss, and postimplantation loss.

In the embryo–fetal development assessment, the numbers of corpora lutea, implantation sites, resorptions, and fetuses were comparable between the treated and control groups. No statistically significant differences were observed in preimplantation or postimplantation loss rates, fetal cranio-caudal length, or fetal body weight (*p* > 0.05).

Overall, administration of *S. dumetorum* tablets at 250 mg/kg body weight referring to the equivalent amounts of *S. dumetorum* extract did not result in statistically significant changes in the reproductive and embryo–fetal development parameters.

#### 2.3.4. Allergenicity Assessment

The local irritant and sensitizing potential of *S. dumetorum* tablets was evaluated in guinea pigs. Following a single dermal application of the tablet suspension at a dose of 250 mg/kg body weight referring to the equivalent amounts of *S. dumetorum* extract, no signs of local irritation were observed throughout the 10-day observation period. Skin reaction scores remained at 0/10 in both the control and treatment groups on each observation day (Table 15).

Repeated epicutaneous application was subsequently performed to assess the potential sensitizing activity of the tablets. No positive skin reactions were observed after either 10 or 20 applications. According to the skin reaction scoring system of S. V. Suvorov and V. V. Chernyshova, all animals in both the control and treatment groups had a score of 0/10 at 1, 24, and 48 h following the challenge application (Table 16). These findings indicate that repeated epicutaneous exposure to *S. dumetorum* tablets did not induce detectable skin sensitization.

To further assess sensitizing potential under conditions in which the skin barrier was bypassed, the tablet suspension was administered subcutaneously in combination with complete Freund’s adjuvant. No positive skin reactions were detected in the treated animals at 15 min, 24 h, or 48 h after the challenge. Overall, *S. dumetorum* tablets did not exhibit local irritant or sensitizing activity in the experimental models used.

#### 2.3.5. Immunotoxicity

The effects of *S. dumetorum* tablets on humoral immune parameters were evaluated in male and female mice following 3 weeks of intragastric administration at doses of 100, 250, and 1000 mg/kg body weight referring to the equivalent amounts of *S. dumetorum* extract. No statistically significant differences were observed in serum TNF-α, IL-6, C-reactive protein, ferritin, total antibody titers, or IgG levels between treated and control animals (*p* > 0.05). The observed values remained comparable to those of the intact and vehicle-control groups in both sexes (Table 17).

To assess the persistence of any potential immunotoxic effects, humoral immune parameters were additionally evaluated 3 weeks after discontinuation of *S. dumetorum* tablets. No significant differences were detected between the treated and control groups in either male or female mice. TNF-α, IL-6, C-reactive protein, ferritin, total antibody titers, and IgG levels remained within the range observed in the control animals (Table 18).

Repeated administration of *S. dumetorum* tablets at doses of 100, 250, and 1000 mg/kg body weight referring to the equivalent amounts of *S. dumetorum* extract did not produce significant changes in the evaluated humoral immune parameters in either male or female mice. The absence of treatment-related alterations was also confirmed 3 weeks after treatment withdrawal, indicating no persistent adverse effect on humoral immune function.

## 3. Discussion

### 3.1. Phytochemical Composition of S. dumetorum Extract and Its Relationship with Biological Activity

The current investigation demonstrated that the hydroethanolic extract of *S. dumetorum* contains a chemically diverse spectrum of secondary metabolites and exhibits pronounced biological activity. TPC was 40.153 ± 2.253 mg GAE/g extract, while TFC reached 40.551 ± 5.563 mg QE/g extract. The relatively high levels of both phenolic and flavonoid compounds indicate that these constituents represent an important chemical fraction of the extract and may contribute substantially to its observed biological properties. TPC obtained in the present study was higher than the maximum value previously obtained for *S. dumetorum* leaves under optimized microwave-assisted extraction conditions (35.23 ± 0.50 mg GAE/g DW) [18]. One possible contributing factor may be differences in the raw plant material, particularly the harvesting period and environmental conditions. Seasonal and climatic factors are known to influence the biosynthesis and accumulation of phenolic compounds in medicinal plants. For example, significant seasonal fluctuations in phenolic constituents and antioxidant activity have been demonstrated, with the timing of harvest affecting the accumulation of individual phenolic compounds [19]. Environmental conditions, including climate and altitude, have also been shown to influence both total phenolic content and the composition of individual phenolic compounds [20]. Therefore, the higher TPC observed in this study may partly reflect differences in the harvesting season and environmental conditions during plant collection, in addition to differences in extraction and sample preparation. The TPC obtained in our study falls within the broad range reported for other *Salvia* species. Previous studies confirmed substantial differences in the phenolic content of aerial parts of *Salvia* plants, with TPC values ranging from 12.67 to 62.46 mg GAE/g DW across 102 samples representing different *Salvia* taxa [21]. Similarly, considerable variation has been reported for *S. officinalis*, with TPC values ranging from 2.02 to 184 mg GAE/g DW, while a value of 25.13 mg GAE/g DW was reported for Iranian *S. officinalis* L. [22]. Other *Salvia* species, including *S. lanceolata* Lam., *S. dolomitica* Codd, and *S. garipensis* E.Mey. ex Benth., have also shown relatively high TPC values of 54.2, 53.0, and 45.6 mg GAE/g DW, respectively [23]. Another study reported a broad range in TPC (63.9–134.4 mg GAE/g plant) of *S. officinalis* and *S. sclarea* L. [24]. A broader investigation of six *Salvia* species from spontaneous Moldavian flora reported TPC values ranging from 22.25 to 118.75 mg GAE/g dry plant material [25]. Differences associated with geographical origin have also been reported within the *Salvia* genus. For example, TPC values of 167.1 mg GAE/g extract were reported for *S. verticillata* L. from Turkey [26], whereas a value of 175.6 mg GAE/g extract was reported for the same species from Serbia [27]. Similarly, *S. sclarea* from Iran showed a TPC of 268 mg GAE/g extract [28], while *S. officinalis* from Romania exhibited a lower value of 65.02 mg GAE/g extract [29]. Such differences between geographically distinct populations further support the potential contribution of environmental and growing conditions to phenolic accumulation in *Salvia* plants.

A similar comparison can be made for TFC. However, interpretation of the difference from our previous findings requires particular consideration of the reference standard used for quantification. In our previous study on *S. dumetorum*, TFC was expressed as rutin equivalents, whereas quercetin equivalents were used in this study. Therefore, direct comparison of the absolute TFC values should be made with caution, as the reference standard can influence the values obtained in colorimetric assays. The TFC obtained in the current study (40.551 ± 5.563 mg QE/g extract) is within the range reported for several *Salvia* species. In ethanol extracts of *S. officinalis* and *S. sclarea*, TPC values of 24.52–95.62 and 29.39–91.02 mg GAE/g, respectively, have been reported, while TFC ranged from 13.73 to 55.38 mg QE/g for *S. officinalis* and from 25.91 to 53.82 mg QE/g for *S. sclarea* [30]. TFC values of 4.68 and 9.1 mg QE/g have been reported for methanolic extracts of *S. chudaei* Batt. & Trab. and *S. officinalis*, respectively [21], whereas substantially higher values have been reported for *S. hierosolymitana* Boiss., *S. eigii* Zohary, and *S. viridis* L. (770.85, 520.60, and 311.36 mg QE/g DW, respectively) [31]. These differences may also be influenced by the plant material, harvesting conditions, extraction procedures, and analytical methods used.

The HPLC-QTOF-ESI-MS/MS analysis demonstrated a complex phenolic profile of the *S. dumetorum* extract, with 39 compounds tentatively identified in the negative ionization mode. The detected metabolites comprised phenolic acids, phenylpropanoids, flavonoids and their glycosides, caffeic acid-derived oligomers, diterpenoids, fatty acids, and organic acids. The predominance of phenolic metabolites is consistent with previous phytochemical investigations of *S. dumetorum* and other *Salvia* species [2,32].

Caffeic acid represents a key biosynthetic precursor and structural building block for a broad range of caffeic acid-derived phenolic metabolites in *Salvia*, from simple phenolic acids to dimers and higher oligomers. It is therefore widely distributed throughout the genus, having been reported in numerous taxa, including *S. africana* L., *S. aethiopis* L., *S. cadmica* Boiss., *S. deserta* Schangin, *S. fruticosa* Mill., *S. glutinosa* L., *S. nemorosa* L., *S. officinalis*, *S. potentillifolia* Boiss. & Heldr. ex, *S. pratensis* L., *S. sclarea*, *S. tomentosa* Mill., *S. verticillata*, *S. verbenaca* L., and *S. virgata* [27,33,34]. Importantly, the present HPLC-QTOF-ESI-MS/MS profiling also revealed a broad spectrum of caffeic acid-derived phenolics, including danshensu, sagerinic acid, lithospermic acids A and B, salvianolic acid K, and yunnaneic acids D–F and rosmarinic acid. In particular, rosmarinic acid and caffeic acid have repeatedly been reported among the major phenolic constituents of *S. dumetorum*. An earlier comparative study of 11 *Salvia* species reported 2.09% rosmarinic acid and 0.08% caffeic acid in *S. dumetorum* [35]. HPLC-DAD analysis of *S. dumetorum* growing in Ukraine previously identified caffeic and rosmarinic acids together with cynaroside, rutin, cirsimaritin, kaempferol-3-O-glucoside, and hispidulin [36]. A recent comprehensive review of phenolic constituents of Greek *Salvia* taxa reported rosmarinic acid in all 21 investigated taxa, highlighting its importance as a characteristic caffeic acid-derived metabolite of the genus, while caffeic acid was likewise described as a prominent phenolic acid occurring across *Salvia* species [32]. Rosmarinic acid is being reported as a predominant compound in several species, including *S. plebeia* R.Br. [33], *S. officinalis* [37], and *S. pratensis* [38], *S. nemorosa* [39], *S. rosmarinus* Spenn. [40], *S. hydrangea* DC. ex Benth. [41], *S. fruticosa* [42], *S. limbata* C.A.Mey. [43], *S. tomentosa* [44], *S. przewalskii* [45]. LC-DAD-QTOF-MS analysis of eight *Salvia* species detected sagerinic acid, rosmarinic acid, and salvianolic acid K across multiple taxa, with sagerinic acid being particularly widespread, while salvianolic acid K was also detected in several species [46]. In *S. africana*, yunnaneic acid E isomers were especially abundant, reaching 74 μg/mg extract and accounting for approximately 32% of the total quantified phenolics; yunnaneic acids D and F, salvianolic acid K, and sagerinic acid were also identified [47]. Similarly, analysis of *S. verbenaca* revealed yunnaneic acids D, E, and F together with salvianolic acid K [13]. In *S. officinalis*, sagerinic acid was quantified at 2.92 ± 0.08 mg/g in an infusion, whereas salvianolic acid K occurred at 6.8–56.4 mg/L in commercial sage teas and at 18.10–50.14 mg/100 g dry matter in hydroethanolic extracts [48]. Piatczak et al. reported that *S. tomentosa* contains lithospermic acid A at 9.34–148.20 mg/kg, while sagerinic acid was detected at 2.03–4.04 mg/kg in some extracts [44]. Nicolì et al. reported danshensu in the shoots of *S. clandestina* L. at 4.96 mg/g dry weight [49]. In *S. miltiorrhiza* Bunge, danshensu has likewise been quantitatively determined, with reported concentrations ranging from 0.34 to 1.85 mg/g in different root samples, while other extraction procedures yielded considerably higher concentrations of up to 14.20 ± 0.40 mg/g extract [50]. Protocatechuic acid and protocatechuic aldehyde were also detected in *S. dumetorum* leaf extract at *m*/*z* 153.0186 and 137.0232, respectively. Both compounds have previously been identified in *S. miltiorrhiza* roots, including heat-reflux and methanolic extracts, where protocatechuic acid and protocatechuic aldehyde were characterized by HPLC-MS/MS at *m*/*z* 153.0191 and 137.0242, respectively [51]. They were also detected in extracts of *S. officinalis* aerial material by HPLC-DAD-MS [52].

Flavonoids constitute another characteristic group of phenolic metabolites in *Salvia*, with flavones and their glycosylated and methylated derivatives being particularly widespread. A comprehensive review of Greek *Salvia* species showed that methylated flavones were also broadly distributed, with genkwanin reported in 13 taxa, hispidulin in 10 taxa, and cirsimaritin in 14 taxa [32]. Quantitative studies further demonstrate substantial variation in flavonoid abundance among species. Hispidulin has been found in *S. officinalis* quantified as 2.24 ± 0.13 mg/g. In another study dedicated to methanolic extract of *S. fruticosa*, genkwanin, hispidulin, and cirsimaritin were identified at *m*/*z* 283.0607, 299.0556, and 313.0712, respectively [53]. Consistent with these previous findings, *S. dumetorum* extract yielded corresponding molecular ions at *m*/*z* 283.0589, 299.0538, and 313.0692, supporting the tentative assignment of genkwanin, hispidulin, and cirsimaritin. Hispidulin and cirsimaritin have also been reported in *S. stepposa* Des.-Shost. leaves from Ukraine in the comparative HPLC-MS study by Koshovyi et al. at concentrations of 461 and 1378 mg/kg, respectively [36].

The present analysis also provided evidence for the occurrence of abietane-type diterpenoids such as hydroxycarnosic acid, rosmanol, and sugiol. Hydroxycarnosic acid has been identified in *S. sclarea*, *S. tesquicola* Klokov & Pobed., *S. nemorosa*, *S. kopetdaghensis* Kudr., *S. rosmarinus*, and *S. apiana* Jeps. [27], whereas rosmanol has been reported in *S. tesquicola*, *S. officinalis*, *S. nemorosa*, *S. pratensis*, *S. rosmarinus*, and *S. apiana* [54,55,56]. Notably, sugiol has previously been isolated and structurally characterized from the roots of *S. deserta* [57] and *S. viridis* [58]. Obtained data expands the known phytochemical composition of *S. dumetorum* by tentatively assigning danshensu, sagerinic acid, lithospermic acids A and B, salvianolic acid K, yunnaneic acids D–F, hydroxycarnosic acid, rosmanol, and sugiol, none of which have previously been documented in this species. This finding highlights a substantially broader diversity of caffeic acid-derived phenolics and abietane-type diterpenoids in *S. dumetorum* than previously recognized.

The phytochemical profiling performed in this study substantially expands the range of metabolites previously documented for *S. dumetorum*, with several of the tentatively assigned metabolites not previously reported for this species in the literature reviewed.

The diverse phenolic composition of the *S. dumetorum* extract, encompassing phenolic acids, flavonoids, caffeic acid-derived oligomers, and abietane-type diterpenoids, provides a plausible chemical basis for its pronounced antioxidant activity. Many of these metabolites contain hydroxylated aromatic structures capable of electron or hydrogen donation and radical stabilization, while the coexistence of structurally distinct constituents may result in additive or synergistic antioxidant effects [59]. Accordingly, the antioxidant activity observed in our study should be considered a property of the complex phytochemical matrix rather than attributed to a single constituent, particularly because the individual contribution of each tentatively identified metabolite was not quantitatively assessed. Consistent with this interpretation, the extract demonstrated high antioxidant activity across all applied assays. TEAC, TAC, and FRAP values reached 1.882 ± 0.074 mM, 250.43 ± 12.08 mg AsAE/g_extr_, and 427.91 ± 9.74 mg TE/g_extr_, respectively, while the DPPH radical-scavenging assay yielded an IC_50_ of 0.031 ± 0.002 mg/mL. The consistent activity observed across these complementary assays strengthens the evidence for the antioxidant capacity of the extract, as the methods involve different reaction mechanisms and therefore assess distinct aspects of redox activity. In particular, the low DPPH IC_50_ indicates a high efficiency of the extract in scavenging DPPH radicals, supporting the substantial radical-neutralizing potential suggested by its phenolic-rich chemical profile. Previous studies have demonstrated pronounced radical-scavenging and reducing capacities among *Salvia* extracts, with antioxidant activity frequently associated with their phenolic composition. In particular, significant correlations have been reported between DPPH/FRAP activity and individual caffeic acid-derived metabolites, including rosmarinic acid, lithospermic acid, salvianic acid, and caffeic acid, as well as luteolin derivatives. In *S. lavandulifolia* Vahl, rosmarinic acid and lithospermic acid showed strong positive correlations with DPPH (r = 0.8937 and 0.8307, respectively) and FRAP (r = 0.7078 and 0.8984, respectively), while caffeic acid was significantly correlated with DPPH activity [60]. Similarly, studies of sage extracts demonstrated strong positive correlations of total phenolic content with DPPH (r = 0.986) and FRAP (r = 0.982), supporting the contribution of the overall phenolic matrix to antioxidant capacity [61]. An earlier comparative study of eleven *Salvia* species also demonstrated pronounced antioxidant activity of *S. dumetorum*, with an EC_50_ of 6.5–10.2 μg/mL in an enzyme-independent lipid peroxidation model, markedly exceeding the activity of ascorbic acid (EC_50_ = 123.8 μg/mL) [35]. Notably, antioxidant activity showed a close correlation with total phenolic content, whereas correlations with individual caffeic and rosmarinic acids were considerably weaker, suggesting that the overall polyphenolic composition rather than a single dominant constituent contributes substantially to the antioxidant activity of *Salvia* extracts. The antioxidant capacity of the *S. dumetorum* extract was comparable with that reported for several other *Salvia* species. The DPPH IC_50_ of the *S. dumetorum* extract was 0.031 ± 0.002 mg/mL (31 ± 2 μg/mL), which was within the range reported for methanolic extracts of several Eastern European *Salvia* species, including *S. officinalis* (25.33 ± 0.05 μg/mL), *S. verticillata* (27.36 ± 0.32 μg/mL), *S. sclarea* (32.23 ± 0.35 μg/mL), and *S. pratensis* (39.53 ± 0.15 μg/mL), while indicating stronger radical-scavenging activity than that reported for *S. nemorosa* (57.40 ± 0.40 μg/mL) and *S. nutans* (178.90 ± 1.10 μg/mL) under comparable assay conditions [27]. Together, the low DPPH IC_50_ and high ferric-reducing capacity observed in this study indicate pronounced antioxidant activity of the extract. A recent study of 70 *S. nemorosa* populations from 54 habitats demonstrated substantial variation in DPPH and FRAP activities among populations, despite the use of the same species and aerial plant material. The authors associated this variability with differences in phytochemical composition and habitat-related factors [62]. Likewise, analysis of 102 *Salvia* samples from Iran demonstrated marked variation in DPPH and FRAP responses among samples; for example, FRAP values ranged from 64.28 ± 3.40 to 1090.55 ± 3.55 μmol Fe^2+^/g DW across individual samples, including *S. verticillata*, *S. nemorosa*, *S. officinalis*, and *S. sclarea* [21]. Because these studies used different reference standards, units, extraction procedures, and sample bases, their absolute FRAP values are not directly comparable with the present value of 427.91 ± 9.74 mg TE/g_extr_. Nevertheless, the available evidence consistently indicates that antioxidant activity within *Salvia* is strongly influenced by the qualitative and quantitative composition of the phenolic matrix as well as by species, population, geographical origin, and extraction conditions.

In addition to its antioxidant properties, the *S. dumetorum* extract exhibited α-amylase inhibitory activity, with an activity of 5.631 ± 0.515 mg AcrE/g_extr_, indicating that its biological effects extend beyond redox-related mechanisms. The α-amylase-inhibitory activity observed for the *S. dumetorum* extract may be related to the combined presence of several phenolic constituents identified by HPLC-QTOF-ESI-MS/MS. Caffeic acid may also contribute to the α-amylase-inhibitory effect, as it has been shown to inhibit porcine pancreatic α-amylase with an IC_50_ of 3.68 μg/mL and to inhibit α-glucosidase in vitro [63]. Several flavones detected in the present extract may provide an additional contribution to enzyme inhibition; apigenin and hispidulin directly inhibited α-amylase, with reported IC_50_ values of 21.66 ± 0.96 and 30.08 ± 1.12 μM, respectively, and combinations of apigenin with hispidulin and related flavones produced synergistic inhibition of the enzyme [64]. Protocatechuic acid, also detected in the *S. dumetorum* extract, was isolated from Kadsura longipedunculata extract and demonstrated direct α-amylase inhibitory activity, with an IC_50_ of 12.5 μmol/L [65]. Isolated rosmarinic acid exhibited an IC_50_ of 0.92 μg/mL against α-amylase, substantially lower than that of the reference inhibitor (17.68 μg/mL), further supporting the direct inhibitory potential of this caffeic acid-derived metabolite [66]. α-Amylase inhibition has likewise been documented for other *Salvia* species, although the magnitude of activity varies considerably depending on species, plant material, extraction procedure, and assay conditions. For example, a phenolic-enriched fraction obtained from the aerial parts of *S. officinalis* exhibited potent α-amylase inhibition, with an IC_50_ of 46.52 ± 2.68 μg/mL [67]. An aqueous extract of *S. lavandulifolia* leaves showed a more moderate inhibitory effect, with an IC_50_ of 0.99 ± 0.00 mg/mL, compared with 0.52 mg/mL for acarbose [66]. In another study methanolic extract of *S. nemorosa* exhibited strong α-amylase inhibition, with an IC_50_ of 4.4 μg/mL, exceeding the activity of acarbose [68]. Recent evaluation of three endemic Turkish *Salvia* species demonstrated considerable interspecific variation in α-amylase inhibition, with *S. vermifolia* Hedge & Hub.-Mor. showing the strongest activity among the tested extracts (IC_50_ = 68.38 ± 4.27 μg/mL) [69]. Earlier comparative work also demonstrated α-amylase inhibition by *S. verticillata* and *S. virgata*, with IC_50_ values of 18.34 and 19.73 mg/mL, respectively [70]. Although these IC_50_ values cannot be directly compared with the acarbose-equivalent activity obtained in the present study because different assay formats, extraction procedures, and expression systems were employed, the available evidence supports the occurrence of α-amylase-inhibitory potential across chemically diverse *Salvia* extracts.

The antimicrobial properties of the crude extract were additionally characterized as part of its preliminary biological profiling. The extract demonstrated broad-spectrum antimicrobial activity, with MIC values ranging from 1.25 to 10 mg/mL. Among the Gram-positive bacteria, *S. aureus*, *S. epidermidis*, and *M. luteus* were the most susceptible species (MIC 1.25 mg/mL), whereas *E. faecalis* showed a higher MIC of 5 mg/mL. Among the Gram-negative bacteria, *K. pneumoniae* was the most susceptible (MIC 2.5 mg/mL), while *S.* Typhimurium, *E. coli*, and *P. aeruginosa* required 10 mg/mL. Among the yeasts, *C. glabrata* showed the lowest MIC (1.25 mg/mL).

The broad antimicrobial activity observed for the *S. dumetorum* extract may be related to the combined presence of several phenolic, flavonoid, and diterpenoid constituents identified by HPLC-QTOF-ESI-MS/MS. Among the detected phenolic acids, caffeic acid showed direct antibacterial activity against *S. aureus*, with MIC values of 256–1024 μg/mL, whereas protocatechuic acid inhibited *S. aureus*, *E. coli*, and *P. aeruginosa* at MIC values of 450, 550, and 300 μg/mL, respectively [71,72]. Rosmarinic acid demonstrated a broader antimicrobial spectrum, with reported MIC values of 0.002–0.8 mg/mL against bacteria and 0.1–0.2 mg/mL against *Candida* spp., together with antibiofilm activity [73]. Among the flavonoids detected in the *S. dumetorum* extract, cirsimaritin showed MIC values of 31.25 μg/mL against *S. aureus*, *E. coli*, and *K. pneumoniae*, 45 μg/mL against *P. aeruginosa*, and 62.5 μg/mL against *S. epidermidis* [74]. Genkwanin likewise demonstrated antibacterial activity, with MIC values of 25 μg/mL against *E. faecalis* and 100 μg/mL against *S. pyogenes*, while additional studies reported activity against *E. coli*, *P. aeruginosa*, and *M. luteus* at 50–100 μg/mL [75]. Acacetin exhibited MIC values of 0.16, 0.32, 0.32, and 0.35 mg/mL against *S. aureus*, *E. faecalis*, *E. coli*, and *P. aeruginosa*, respectively [76]. Luteolin, corresponding to the aglycone of the luteolin-derived glycoside tentatively identified in the extract, showed MIC values of 32 μg/mL against both *E. faecalis* and *C. albicans* and also inhibited biofilm formation [77]. Epigallocatechin similarly demonstrated antibacterial activity, with MIC values of 160 μM against *S. aureus* and *S.* Typhimurium and 320 μM against *E. coli* [78]. The diterpenoid fraction may provide an additional contribution, as recent studies of *S. officinalis* isolated sugiol together with ferruginol and sclareol and demonstrated significant antimicrobial activity against clinical *S. aureus* strains, with the resulting formulation showing MIC values of 0.09–0.74 mg/mL and pronounced antibiofilm effects [79]. In addition, antimicrobial activity of rosmarinol-type diterpenoids has been documented in *Salvia* and related Lamiaceae, although evidence for the activity of individual hydroxycarnosic acid and rosmanol remains more limited.

In a comparative study of ten Eastern European *Salvia* species, MIC values against *S. aureus* ranged from 0.625 to 5 mg/mL, while *E. coli* and *P. aeruginosa* showed MICs of 2.5–5 and 2.5 mg/mL, respectively; *C. albicans* showed MICs of 1.25–5 mg/mL depending on the species [27]. A phenolic-acid-enriched extract of *S. bulleyana* Diels similarly inhibited *S. epidermidis* at 1.25 mg/mL and *S. aureus*, *E. coli*, and *P. aeruginosa* at 2.5 mg/mL, whereas its activity against *C. albicans* and *C. glabrata* was considerably weaker (MIC 10 mg/mL) [80]. Other recent studies further demonstrate substantial variability among *Salvia* species and extraction types, with MIC values ranging from 1.25 to 5 mg/mL for several *Salvia* extracts, while some extracts of *S. glutinosa* and *S. splendens* Sellow ex Schult. showed MIC values as high as 20 mg/mL [81,82]. Thus, the MIC of 1.25 mg/mL observed for *S. aureus*, *S. epidermidis*, and *C. glabrata* in the *S. dumetorum* extract indicates appreciable antimicrobial activity, particularly against these susceptible strains. The lower susceptibility of several Gram-negative bacteria may be related to differences in cell-envelope structure and permeability; however, the observed activity against both bacterial and fungal strains suggests that the extract contains multiple constituents capable of acting on different microbial targets.

Beyond expanding the known phytochemical profile of *S. dumetorum*, the current study provides an integrated evaluation of its antioxidant, α-amylase-inhibitory, and antimicrobial properties within the same hydroethanolic leaf extract. In particular, the α-amylase-inhibitory activity represents an additional biological effect for *S. dumetorum* that has not been characterized in the previous phytochemical and bioactivity studies of this species, thereby extending the pharmacological profile of *S. dumetorum*.

### 3.2. Impact of Tablet Formulation on the Measurable Phytochemical Characteristics and Biological Activities of S. dumetorum Extract

The incorporation of the *S. dumetorum* extract into the tablet formulation resulted in differences in the measured phytochemical and biological characteristics compared with the original extract. These differences should be interpreted in the context of the composition of the final dosage form. Each tablet contained 50 mg of the dry extract within a total tablet mass of 750 mg, corresponding to approximately 6.7% of the tablet weight, while the remaining mass consisted of pharmaceutical excipients. Therefore, when the results are expressed per gram of the final tablet formulation, lower values may, at least in part, reflect the lower proportion of extract-derived constituents within the tablet matrix [83]. The effect of extract concentration within the formulation has also been demonstrated in antioxidant tablets prepared from *Ficus auriculata* Lour. leaf extract, where antioxidant activity increased with increasing extract content in the formulations [84]. The tablets demonstrated measurable TPC, whereas flavonoids were not detected under the applied experimental conditions. Importantly, the absence of a measurable TFC signal should not be interpreted directly as evidence of complete degradation or loss of flavonoid constituents during tablet manufacture. The measured response may also be influenced by the lower concentration of extract-derived constituents in the formulated product and by differences in the analytical response of the tablet matrix. A formulation-dependent effect has also been reported for *Cymbopogon citratus* (DC) Stapf. extract, where the type of excipient influenced dissolution behavior and the stability of phenolic constituents [85]. The differences observed between the TPC and antioxidant activity results further indicate that these parameters should not be interpreted interchangeably. Although the Folin–Ciocalteu method is widely used for estimating total phenolic content, the assay primarily reflects the reducing capacity of the analyzed sample and may be influenced by the chemical composition of the sample and other reducing substances. Consequently, similar or measurable TPC values do not necessarily correspond to proportional antioxidant responses in individual assays [86].

A more pronounced reduction was observed in the antioxidant assays. Compared with the dried extract, the tablet formulation showed lower TEAC, TAC, and FRAP values, while the DPPH radical-scavenging activity decreased to an IC_50_ value of >1.0 mg/mL. Given that the extract represented only approximately 6.7% of the total tablet mass, these lower responses may partly reflect dilution of extract-derived antioxidant constituents by the excipient matrix. In addition, the presence of excipients and the formation of a solid dosage form may influence the recovery and measurable availability of individual constituents during sample preparation and analysis [87]. Furthermore, the antioxidant assays applied in this study are based on different chemical principles and may therefore respond differently to changes in sample composition and the availability of antioxidant constituents. Consequently, the differences observed between the extract and tablet formulation should not necessarily be interpreted as evidence of chemical degradation of bioactive constituents. In another study, the influence of solid formulation and processing conditions on the measurable antioxidant activity of plant extracts was investigated using liquid–solid tablets. Extracts of *Aronia melanocarpa* (Michx.) Britton, *Crataegus laevigata* (Poir.) DC., and *Rosa canina* L. were incorporated into solid dosage forms using a porous magnesium aluminometasilicate carrier together with conventional excipients, and their antioxidant activity was evaluated by the FRAP assay after dissolution [88]. The authors found that the antioxidant response of the resulting tablets was influenced by the type of plant extract, the solvent used for extract incorporation, and the drying conditions. In particular, the use of water and the drying process generally reduced the measured antioxidant activity, although the magnitude of this effect differed among the investigated extracts. These findings demonstrate that the measurable antioxidant capacity of a plant extract can be affected by its incorporation into a solid dosage form and by subsequent formulation and processing conditions.

In contrast to the pronounced changes observed for several antioxidant parameters, α-amylase inhibitory activity showed a less pronounced reduction following incorporation of the *S. dumetorum* extract into the tablet formulation. The tablets retained measurable enzyme-inhibitory activity of 4.539 ± 0.183 mg AcrE/g_tablet_ compared with 5.631 ± 0.515 mg AcrE/g_extr_ for the dried extract. Considering the substantially lower proportion of extract in the final tablet formulation, the persistence of measurable α-amylase inhibitory activity suggests that the formulation retained, at least in part, the enzyme-inhibitory properties associated with the plant extract. Comparable findings have been reported for other plant-based solid formulations. A recent study on *Hymenocardia punctata* Wall. ex Lindl. demonstrated that incorporation of the plant extract into orodispersible tablets containing croscarmellose sodium and microcrystalline cellulose resulted in a formulation that retained α-amylase inhibitory activity together with antioxidant and α-glucosidase inhibitory properties [89]. Similarly, Sharma et al. evaluated different dosage forms of *Tinospora cordifolia* (Willd.) Hook.f. & Thomson and demonstrated measurable α-amylase inhibitory activity in the formulated preparations, although the magnitude of inhibition varied among the different dosage forms [90]. These findings indicate that enzyme-inhibitory activity of plant-derived materials can persist following incorporation into a solid dosage form, while the extent of the measured activity may depend on the formulation and analytical conditions. A further example was reported by Riaz et al., who compared a polyherbal extract with its formulated granules and found that α-amylase inhibitory activity was retained following conversion of the extract mixture into a solid formulation [91]. Notably, the formulated granules showed an IC_50_ of 1.47 mg/mL compared with 1.86 mg/mL for the extract mixture, demonstrating that conversion into a solid formulation does not necessarily result in a loss of enzyme-inhibitory activity and may, depending on the formulation, modify the measured response.

Overall, the results demonstrate that the transition from the dried *S. dumetorum* extract to a tablet dosage form was associated with changes in the measured phytochemical and biological parameters. These differences are likely influenced primarily by the lower proportion of extract in the final formulation and may additionally reflect matrix-related effects on the measurable response of extract constituents. Further quantitative analysis of selected individual marker compounds in both the extract and the final tablet formulation would help determine whether the observed differences are associated with changes in chemical composition or predominantly reflect differences related to formulation and analytical measurement.

### 3.3. Pharmacological Relevance and Preclinical Safety of the Tablet Formulation

The biological activity demonstrated for the *S. dumetorum* extract was complemented by evidence of pharmacological activity of the final tablet formulation in vivo. In the carrageenan-induced paw edema model, the *S. dumetorum* extract-based tablets produced a dose-dependent and statistically significant reduction in paw volume at 3, 12, and 24 h after induction of inflammation. The most pronounced effect was observed at doses above 250 mg/kg, at which the anti-inflammatory activity was comparable to that of diclofenac sodium and greater than that observed for the herbal preparation Rotokan. These findings are particularly relevant because the anti-inflammatory effect was demonstrated using the final solid dosage form rather than the dried extract alone, providing evidence that the pharmacological properties of the plant-derived material were retained following formulation. The observed anti-inflammatory activity is consistent with previous studies demonstrating the potential of *Salvia* extracts in models of acute inflammation. For example, an ethanol extract of *Salvia argentea* L. produced a dose-dependent reduction in carrageenan-induced paw edema and decreased serum C-reactive protein levels, together with suppression of pro-inflammatory mediators including IL-1β, IL-6, and TNF-α [92]. Similarly, an ethanolic extract of *Salvia lachnostachys* Benth. significantly inhibited carrageenan-induced paw edema in mice in a dose-dependent manner [93]. Importantly, direct evidence that formulation into a tablet can preserve anti-inflammatory activity has also been reported for *Phyllanthus emblica* L. [94]. Hydroalcoholic leaf extract and its tablet formulation significantly reduced carrageenan-induced paw edema, with inhibition values of 66.41% and 69.43%, respectively, compared with the carrageenan control; the effect of the tablet was close to that of diclofenac (70.18%). Taken together, these observations support the relevance of evaluating the pharmacological activity of the finished dosage form rather than relying exclusively on biological activity measurements performed with the starting extract.

The pharmacological findings were complemented by a series of repeated-dose safety studies. Repeated intragastric administration of the *S. dumetorum* tablets at 100, 250, and 1000 mg/kg for three weeks did not result in treatment-related adverse changes in general clinical condition, locomotor activity, body weight, food or water consumption, cardiac electrophysiology, hematological or biochemical parameters, renal function, or gross organ morphology. Moreover, the absence of persistent treatment-related changes after the three-week recovery period supports the reversibility of any minor physiological variations and indicates favorable tolerability of the formulation under the tested experimental conditions. Our previous acute oral toxicity study established the initial tolerability of the *S. dumetorum* tablet formulation, with no mortality or treatment-related signs of toxicity following single-dose administration [7]. The present study extends these findings by evaluating the effects of repeated exposure and assessing the reversibility of any potential treatment-related changes. Thus, the present findings provide a subsequent level of evidence by addressing repeated exposure and recovery rather than single-dose tolerability alone. Taken together, the acute and repeated-dose studies provide complementary information on the tolerability of the tablet formulation and support its further preclinical evaluation.

The absence of treatment-related effects was also observed across a range of immune-related endpoints. Following three weeks of administration, serum concentrations of TNF-α, IL-6, C-reactive protein, and ferritin, as well as total antibody titers and IgG, did not differ significantly between treated and control animals. The values remained comparable across the tested doses and between male and female animals. Importantly, assessment after the recovery period likewise revealed no significant differences in these parameters, indicating the absence of persistent alterations in humoral immune status. The biochemical findings were further supported by assessment of lymphoid organs. Thymic and splenic cellularity and organ-related parameters remained comparable with those of the control groups, providing no evidence of treatment-related structural or functional impairment of the major immune organs under the experimental conditions. Therefore, the combined evaluation of humoral immune markers and lymphoid-organ parameters does not indicate immunotoxicity associated with repeated administration of the *S. dumetorum* tablets at the tested doses. The reproductive and embryofetal studies provided additional information regarding the safety profile of the formulation. Repeated administration of the tablets at 250 mg/kg did not produce treatment-related changes in female sexual behavior or estrous-cycle distribution. Although the pregnancy index was numerically 16.6% higher in treated animals, this difference was not statistically significant and therefore should not be interpreted as evidence of an improvement in reproductive performance. The numbers of corpora lutea, implantation sites, and fetuses, as well as preimplantation and postimplantation loss and time to conception, remained comparable with the control groups. Similarly, administration of the tablets during the organogenesis period at 250 mg/kg did not result in evidence of maternal or embryofetal toxicity. Minor numerical variations in corpora lutea, implantation sites, viable fetuses, and resorptions were not statistically significant and remained within the observed physiological range. Fetal body weight was approximately 8% higher than in the vehicle-control group; however, this difference was not accompanied by changes in crown–rump length, skeletal ossification, or visceral morphology. No developmental abnormalities or malformations were detected on macroscopic or histological examination. Accordingly, the numerical increase in fetal body weight should not be interpreted as a treatment-related developmental effect in the absence of accompanying developmental changes. The overall reproductive and embryofetal findings therefore provide no evidence of adverse effects under the conditions investigated.

Finally, the tablet formulation demonstrated a favorable profile in the local irritation and sensitization models. A single dermal application of the tablet suspension at 250 mg/kg produced no local irritation during the 10-day observation period, with skin reaction scores remaining at 0/10 in all observations. Repeated epicutaneous application likewise produced no positive skin reactions after either 10 or 20 applications. Furthermore, no positive reactions were detected following challenge after administration of the tablet suspension in combination with complete Freund’s adjuvant. Thus, no detectable local irritant or sensitizing activity was observed in the experimental models employed.

Overall, the findings provide an integrated preclinical characterization of the *S. dumetorum* tablet formulation. The formulation retained measurable pharmacological activity in vivo, including a dose-dependent anti-inflammatory effect, while repeated administration was not associated with clinically relevant adverse changes in physiological, hematological, biochemical, cardiac, renal, or morphological parameters. In addition, no evidence of treatment-related immunotoxicity, impairment of female reproductive function, embryofetal developmental toxicity, local irritation, or sensitization was detected under the tested conditions. Together with the previously established acute oral tolerability of the formulation, these findings support the continued preclinical development of *S. dumetorum* tablets and provide a basis for further investigation of their pharmacological efficacy and safety.

## 4. Materials and Methods

### 4.1. Chemicals and Drugs

All chemicals and reagents used in this study were of analytical grade or HPLC grade, as appropriate. Ethanol, Folin–Ciocalteu reagent, gallic acid, quercetin, sodium carbonate, aluminum chloride, sodium nitrite, sodium hydroxide, 2,2-diphenyl-1-picrylhydrazyl, Trolox, ascorbic acid, acetonitrile (HPLC grade), formic acid, rutin, ABTS, potassium persulfate (K_2_S_2_O_8_), sodium phosphate, hydrochloric acid, 2,4,6-tris(2-pyridyl)-S-triazine (TPTZ), ferric chloride (FeCl_3_·6H_2_O), sodium acetate, sulfuric acid, sodium phosphate, ammonium molybdate, α-amylase, acarbose, soluble starch, iodine/potassium iodide (I_2_/KI), and dimethyl sulfoxide (DMSO) were used in the phytochemical and biological assays. Microcrystalline cellulose (Foodchem International Corporation, Shanghai, China; food grade), α-lactose monohydrate (Merck KGaA, Darmstadt, Germany; EMPROVE^®^ ESSENTIAL, Ph. Eur., BP, ChP, JP, NF; special grade for tabletting), citric acid (Sigma-Aldrich, St. Louis, MO, USA); anhydrous; analytical grade, compliant with Ph. Eur., BP, and USP; purity 99.5–100.5%), and calcium stearate (Sigma-Aldrich, St. Louis, MO, USA; Ca content 6.6–7.4%) were used for tablet production. All chemicals were purchased from (Sigma-Aldrich, St. Louis, MO, USA; Merck KGaA, Darmstadt, Germany), or equivalent suppliers, and distilled water was used throughout all procedures.

### 4.2. Animals and Experimental Design

All animal experiments were conducted in accordance with the principles of the European Convention for the Protection of Vertebrate Animals Used for Experimental and Other Scientific Purposes, Directive 2010/63/EU [95], applicable Good Laboratory Practice (GLP) requirements, and relevant guidelines for the preclinical safety evaluation of medicinal products. The studies were designed considering the intended oral route of administration of the tablet formulation containing dry *S. dumetorum* extract. Laboratory animals were obtained from an accredited breeding facility and underwent a 14-day quarantine and acclimatization period before initiation of the experiments. During this period, animals were clinically examined and body weight was monitored; animals showing signs of disease or other abnormalities were excluded. Animals were housed under controlled vivarium conditions with free access to standard laboratory chow and drinking water. Following acclimatization, animals were randomly allocated to experimental groups according to body weight, with individual identification maintained throughout the studies. All experimental procedures were performed under standardized conditions, and appropriate measures were taken to minimize animal discomfort and the number of animals used.

All experimental procedures were performed under standardized conditions, with appropriate measures taken to minimize animal discomfort and the number of animals used. The detailed endpoints and analytical procedures for each experimental model are described in the corresponding subsections below.

### 4.3. Preparation of S. dumetorum Extract and Tablet Formulation

#### 4.3.1. Plant Material

Leaves of *Salvia dumetorum* Andrz. ex Besser were collected in July 2025 in the vicinity of Karaganda, Kazakhstan (49°88.898′ N, 73°15.569′ E). The plant material was taxonomically authenticated by botanist Prof. Dr. M.Yu. Ishmuratova, and a voucher specimen (QAR00068) was deposited in the herbarium of E.A. Buketov Karaganda University. The freshly collected leaves were air-dried under shade at 25–30 °C for seven days, protected from direct sunlight to minimize degradation of thermolabile and photosensitive constituents. The moisture content of the dried and milled plant material was 4.2%, determined as the loss on drying in compliance with the Eurasian Economic Union Pharmacopoeia (Volume I, Chapter 2.1.2.31) [96]. The dried leaves were subsequently ground to a powder and passed through a stainless-steel sieve with a mesh size of 1.4 mm.

#### 4.3.2. Preparation of *S. dumetorum* Extract

The plant material was extracted using the microwave-assisted extraction technique according to the previously described procedure [18]. Briefly, 100 g of powdered *S. dumetorum* leaves were mixed with 1000 mL of 40% (*v*/*v*) ethanol and subjected to microwave irradiation in an ETHOS X microwave extraction system (Milestone, Sorisole, Italy) at 600 W for 6 min. The extraction procedure was performed three times. The resulting extracts were filtered through a paper filter and concentrated under reduced pressure using a rotary evaporator RV 8 (IKA, IKA-Werke GmbH & Co. KG, Staufen, Germany). The concentrate was subsequently further evaporated in a water bath maintained at 55 °C. The final dry extract yield was 22.01% based on the dry weight of the initial plant material.

#### 4.3.3. Tablet Formulation

Tablets containing *S. dumetorum* leaf extract were manufactured by direct compression using a 10 mm punch on a hydraulic press at a compression pressure of 1400 psi [97]. The formulation consisted of 50 mg of dry extract together with microcrystalline cellulose (75 mg), α-lactose monohydrate (589.5 mg), citric acid (28 mg), calcium stearate (7.5 mg). All ingredients were accurately weighed, passed through a sieve, and thoroughly blended to ensure uniform distribution. Calcium stearate was subsequently incorporated as a lubricant, followed by additional mixing. The moisture content of the powder blend was determined before compression in accordance with the Eurasian Economic Union Pharmacopoeia (Volume I, Chapter 2.1.2.31, “Loss on drying”) and was found to be 5–6% [96]. A total of 400 tablets (SDT5) were manufactured. During production, the tablets were subjected to routine quality control testing, including assessment of weight uniformity, mechanical strength, friability, and dimensional characteristics.

#### 4.3.4. Technical Specification of the Extract and Quality Control of Tablets

The quality control of the *S. dumetorum* extract and the tablets containing this extract was performed in accordance with the Pharmacopoeia of the Eurasian Economic Union (EAEU Pharmacopoeia), as applicable [96]. The dry extract was evaluated for appearance, solubility, identification of phenolic compounds, rosmarinic acid content, loss on drying, heavy metals, and microbiological purity. Phenolic compounds were identified using qualitative reactions with potassium dichromate and ferric chloride, while rosmarinic acid was quantified by HPLC using the external standard method. Tablet appearance was assessed according to the general monograph “Tablets” EAEU Pharmacopoeia, general chapter 2.1.6.0. Identification and testing for related substances were performed in accordance with the relevant normative documentation. Uniformity of mass was evaluated according to EAEU Pharmacopoeia, general chapter 2.1.9.5. Disintegration and dissolution were determined according to EAEU Pharmacopoeia, general chapter 2.1.9.3. Tablet friability was determined using an AE-1 friability tester (Charles Ischi AG, Zuchwil, Switzerland) according to EAEU Pharmacopoeia, general chapter 2.1.9.6. Resistance to crushing was measured using an HC 6.2 tablet hardness tester (Kraemer Elektronik GmbH, Darmstadt, Germany) according to EAEU Pharmacopoeia, general chapter 2.1.9.7.

### 4.4. Phytochemical Analysis

#### 4.4.1. HPLC-ESI-QTOF-MS/MS Analysis

Phytochemical characterization of the *S. dumetorum* extract was conducted by liquid chromatography-mass spectrometry using an Agilent 1200 HPLC system interfaced with an Agilent 6210 Quadrupole Time-of-Flight (QTOF) mass spectrometer (Agilent Technologies, Santa Clara, CA, USA). Ionization was achieved with a Dual AJS electrospray ionization source operated in the negative-ion mode (ESI^−)^. The chromatographic separation was achieved on a Zorbax Eclipse Plus RP-18 chromatographic column by Agilent Technologies (Santa Clara, CA, USA). The column’s dimensions were: 150 mm × 2.1 mm, and the particle size: dp = 3.5 μm. Chromatographic separation was performed with a binary mobile-phase system comprising solvent A (water containing 0.1% formic acid) and solvent B (acetonitrile containing 0.1% formic acid). The gradient was programmed as follows: the proportion of solvent A was maintained at 99% from 0 to 2 min, followed by a linear decrease to 80% between 2 and 10 min. From 10 to 15 min, the composition was adjusted to 60% A, after which a further gradient was applied to reach 5% A at 17 min. This composition was held until 22 min, followed by re-equilibration to the starting conditions of 99% A and 1% B from 22.1 to 25 min. The mobile phase was delivered at 0.2 mL/min, and the chromatographic analysis was conducted over a total period of 30 min. UV absorption was monitored at 290 nm. Samples were introduced in standard injection mode at a volume of 5 μL. Mass spectrometric data were collected in Auto MS/MS acquisition mode. The full-scan MS range was *m*/*z* 100–1400, whereas MS/MS spectra were recorded over *m*/*z* 60–1400. Both MS and MS/MS measurements were obtained at an acquisition rate of one spectrum per second. The instrumental settings were as follows: capillary voltage, 3000 V; nozzle voltage, 1000 V; fragmentor voltage, 110 V; skimmer voltage, 65 V; and octopole RF peak voltage, 750 V. Nitrogen served as both the drying and sheath gas, supplied at 12 L/min. The drying gas and sheath gas temperatures were maintained at 250 and 300 °C, respectively, while the nebulizer pressure was set to 35 psig. Instrument control, data processing, and metabolite annotation were performed using MassHunter software, version 10.0. Compound assignments were based on an integrated assessment of chromatographic retention times, UV absorption characteristics, and the interpretation of observed MS/MS fragmentation pathways. The obtained spectral information was compared with entries available in MassBank [98], PubChem [99], and relevant published studies. Metabolite annotations were subsequently classified in accordance with the recommendations of the Metabolomics Standards Initiative (MSI) [100].

#### 4.4.2. Determination of TPC and TFC

TPC of the *S. dumetorum* extract and tablet samples was determined spectrophotometrically using the Folin–Ciocalteu method [101]. Methanol was used to prepare the extract dilutions and gallic acid standard solutions. For the assay, 20 µL of the test solution was combined with 1560 µL of ultrapure water and 100 µL of Folin–Ciocalteu reagent in 96-deep-well plates using a 12-channel micropipette. Following an incubation period of 1–8 min, 300 µL of 20% sodium carbonate solution was added, and the mixture was thoroughly homogenized. The reaction mixtures were then kept in the dark at 25 °C for 2 h. Subsequently, 300 µL of each reaction mixture was transferred to a 96-well microplate, and the absorbance was measured at 760 nm. Quantification was performed using a five-point calibration curve prepared with gallic acid at concentrations ranging from 0.01 to 1.0 mg/mL. The calibration equation was y = 0.7897x + 0.0606, with a coefficient of determination (*R*^2^) of 0.9915 (Figure S40). All measurements were performed in triplicate. The TPC was calculated from the calibration curve and expressed as milligrams of gallic acid equivalents per gram of dry extract (mg GAE/g_extr_) for the extract samples. For the tablet formulation, the result was expressed as milligrams of gallic acid equivalents per gram of tablet powder (mg GAE/g_tablet_), based on the dry mass of the analyzed tablet material.

TFC of the *S. dumetorum* extract and tablet samples was determined using the aluminum chloride colorimetric assay with quercetin as the reference standard, according to a previously described procedure [102]. Briefly, 80 µL of either the test sample or quercetin standard solution was mixed with 80 µL of aluminum chloride solution and 1840 µL of absolute ethanol in 96-deep-well plates. For the reagent blank, aluminum chloride was replaced with 10 µL of 99.8% acetic acid. The reaction mixtures were incubated in the dark for 40 min. Thereafter, 300 µL of each mixture was transferred to a 96-well microplate, and absorbance was recorded at 415 nm using a microplate reader. Quantification was based on a seven-point quercetin calibration curve covering the concentration range of 0.01–1.0 mg/mL. The resulting calibration equation was y = 1.6177x + 0.0479, with *R*^2^ = 0.9987 (Figure S41). All determinations were conducted in triplicate. The TFC was calculated from the quercetin calibration curve and expressed as milligrams of quercetin equivalents per gram of dry extract (mg QE/g_extr_) for the extract samples and as milligrams of quercetin equivalents per gram of tablet powder (mg QE/g_tablet_) for the tablet formulation.

### 4.5. Assessment of Biological Activity

#### 4.5.1. Trolox Equivalent Antioxidant Capacity Assay (TEAC)

The free-radical-scavenging capacity of the extract and tablet samples toward the ABTS^•+^ radical cation was evaluated using the method originally proposed by Re et al. [103], with minor modifications were made by Zengin et al. [104]. The ABTS^•+^ working radical was generated by combining 7 mM ABTS with 2.5 mM potassium persulfate (K_2_S_2_O_8_) in 10 mL of ultrapure water, followed by incubation in the dark at 22 °C for 16 h. Before use, the resulting ABTS^•+^ solution was diluted with absolute ethanol until an absorbance of 0.7–0.8 was obtained at 734 nm. Test solutions of the extract and tablet samples were prepared in methanol at a concentration of 1 mg/mL. Trolox standard solutions were prepared in methanol over the concentration range of 0.125–3.0 mM (0.125, 0.25, 0.5, 1.0, 2.0, and 3.0 mM). For the reaction, 10 µL of either the sample or Trolox solution was added to 990 µL of the ABTS^•+^ working solution and thoroughly mixed. The control consisted of 10 µL of methanol combined with 990 µL of the radical solution. After incubation for 30 min at 22 °C, the decrease in absorbance was measured at 734 nm. The antioxidant capacity was quantified using the Trolox calibration curve, described by the equation *y* = 23.075*x* − 2.5945, with a coefficient of determination (*R*^2^) of 0.9924 (Figure S42). The ABTS^•+^ scavenging activity was expressed as Trolox equivalent antioxidant capacity (TEAC, mM), calculated from the linear regression equation obtained for the Trolox standards.

#### 4.5.2. Total Antioxidant Capacity (TAC): Phosphomolybdenum Assay

TAC of the *S. dumetorum* extract and tablet samples was determined using the phosphomolybdenum assay described by Zengin et al. [105], with appropriate adaptations for the present study. Sample solutions were prepared at a concentration of 1 mg/mL and mixed with freshly prepared phosphomolybdenum reagent containing 0.6 M sulfuric acid, 28 mM sodium phosphate, and 4 mM ammonium molybdate. The resulting mixtures were incubated at 95 °C for 90 min and subsequently allowed to cool to 22 °C.

Following incubation, absorbance was measured spectrophotometrically at 695 nm. TAC was quantified against an ascorbic acid calibration curve (Figure S43). For the extract, the results were expressed as milligrams of ascorbic acid equivalents per gram of dry extract (mg AsAE/g_extr_), whereas for the tablet formulation, the values were expressed as milligrams of ascorbic acid equivalents per gram of tablet powder (mg AsAE/g_tablet_).

#### 4.5.3. Ferric Reducing Antioxidant Power (FRAP)

Ferric-reducing antioxidant capacity was determined using a modified FRAP procedure based on the method reported by Wong et al. [106]. Three solutions were prepared separately: 300 mM acetate buffer adjusted to pH 3.6, 10 mM 2,4,6-tris(2-pyridyl)-S-triazine (TPTZ) prepared in 40 mM hydrochloric acid, and 20 mM ferric chloride hexahydrate (FeCl_3_·6H_2_O). Immediately before analysis, the FRAP reagent was prepared by combining the acetate buffer, TPTZ solution, and FeCl_3_ solution in a volume ratio of 10:1:1. For the assay, 15 µL of each sample solution prepared in methanol containing 10% dimethyl sulfoxide (DMSO) was mixed with 285 µL of freshly prepared FRAP reagent. The reaction mixtures were incubated at 37 °C for 30 min in the dark, after which the absorbance of the developed colored complex was measured at 593 nm. Trolox was used to generate the calibration curve (Figure S44). The ferric-reducing antioxidant capacity was calculated from the Trolox calibration curve and expressed as milligrams of Trolox equivalents per gram of dry extract (mg TE/g_extr_) for the extract samples. For the tablet formulation, the results were normalized to the mass of tablet powder and reported as milligrams of Trolox equivalents per gram of tablet (mg TE/g_tablet_).

#### 4.5.4. Free Radical Scavenging Activity (DPPH Assay)

The free-radical-scavenging activity of the *S. dumetorum* extract and tablet samples was assessed using the DPPH assay based on the procedure of Brand-Williams et al. [107], with minor modifications were made by Sarikurkcu et al. [108] The method relies on the reduction of the stable purple-colored DPPH radical through hydrogen-atom or electron transfer, resulting in a decrease in absorbance. A fresh DPPH solution was prepared daily at a concentration of 0.08 mg/mL in methanol. Between measurements, the reagent was protected from light and stored at 4 °C. Serial dilutions of the test samples were prepared in methanol containing 10% DMSO over the concentration range of 1.0–0.0078 mg/mL. Reference antioxidant solutions were prepared over the following ranges: gallic acid, 0.1–0.00078 mg/mL; ascorbic acid, 0.50–0.0039 mg/mL; and Trolox, 0.75–0.0058 mg/mL. For each measurement, 100 µL of the corresponding sample, tablet/extract, or reference standard solution was transferred into a well of a 96-well flat-bottom microplate and combined with 100 µL of the DPPH working solution using an Eppendorf multichannel pipette (Eppendorf SE, Hamburg, Germany). The reaction mixtures were incubated in the dark for 30 min at 22 °C. Control wells contained 100 µL of methanol instead of the test solution and 100 µL of DPPH solution. Methanol-filled wells were also included as background controls. After incubation, the absorbance was measured at 517 nm. All measurements were carried out in triplicate. The DPPH radical-scavenging capacity was calculated from the decrease in absorbance relative to the control and expressed as percentage inhibition according to the following equation:

$$\mathrm{Inh}\%=(\frac{{\mathrm{Abs}}_{\mathrm{control}}-{\mathrm{Abs}}_{\mathrm{sample}}}{{\mathrm{Abs}}_{\mathrm{control}}})\times 100$$

where *Abs_control_*—absorbance of control, *Abs_sample_*—absorbance of sample.

#### 4.5.5. Antimicrobial Activity

The antimicrobial activity of the extract was assessed by the broth microdilution method following the guidelines of the European Committee on Antimicrobial Susceptibility Testing (EUCAST) [109]. The antibacterial assay included selected Gram-positive strains, namely *Staphylococcus aureus* ATCC 25923, *Staphylococcus aureus* ATCC BA1707, *Staphylococcus epidermidis* ATCC 12228, *Micrococcus luteus* ATCC 10240, *Bacillus cereus* ATCC 10876, and *Enterococcus faecalis* ATCC 29212, as well as Gram-negative strains, including *Salmonella* Typhimurium ATCC 14028, *Escherichia coli* ATCC 25922, *Proteus mirabilis* ATCC 12453, *Klebsiella pneumoniae* ATCC 13883, and *Pseudomonas aeruginosa* ATCC 9027. Mueller–Hinton (MH) broth was used as the culture medium for the bacterial assays. Antifungal activity was investigated using the yeast strains *Candida glabrata* ATCC 90030, *Candida albicans* ATCC 102231, and *Candida parapsilosis* ATCC 22019. For yeast cultivation, MH broth supplemented with 2% glucose was employed. Prior to the assay, the extract was dissolved in DMSO, diluted with the corresponding broth medium, and subjected to serial two-fold dilution. The resulting dilutions were dispensed into sterile 96-well microtiter plates (Nunc, Roskilde, Denmark), providing final extract concentrations ranging from 20 to 0.156 mg/mL. Fresh microbial cultures were suspended in sterile 0.85% NaCl solution and adjusted to a turbidity equivalent to the 0.5 McFarland standard. The standardized suspensions were then added to the wells to obtain final inoculum concentrations of 5 × 10^5^ CFU/mL for bacterial strains and 5 × 10^4^ CFU/mL for yeast strains. The plates were incubated at 35 °C for 18–20 h. Following incubation, MIC was determined by assessing microbial growth inhibition based on turbidity, using both visual inspection and spectrophotometric measurement. Absorbance was recorded at 600 nm with a BioTek ELx800 microplate reader (BioTek^®^ Instruments, Inc., Winooski, VT, USA). MIC was defined as the lowest concentration of the extract at which no visible microbial growth was detected. MBC and MFC were determined by transferring 5 μL aliquots from the corresponding wells onto MH agar plates free of the extract. The plates were subsequently incubated at 35 °C for 24 h. The lowest extract concentration resulting in complete inhibition of microbial growth was recorded as MBC or MFC, as applicable. All antimicrobial assays were conducted in triplicate, and the highest value obtained was reported. The assay included the following controls: a DMSO control with a final concentration of 1% (*v*/*v*), a positive control consisting of inoculum without extract, and a negative control containing extract without inoculum.

#### 4.5.6. *α*-Amylase Enzyme Inhibition Assay

The inhibitory activity of the samples toward α-amylase was determined using the Caraway–Somogyi iodine/potassium iodide (I_2_/KI) assay, with slight modifications were made by Xiao et al. and Zengin et al. [110,111]. The α-amylase from porcine pancreas, type VI-B (Sigma-Aldrich, St. Louis, MO, USA) at concentration 0.8 U/mL was used in enzyme inhibition test. The extract samples were tested at concentration 10 mg/mL. The substrate was prepared as a 0.05% soluble potato starch solution by dissolving 10 mg of starch in 20 mL of ultrapure water, followed by heating at boiling temperature for 10 min and subsequent cooling to 22 °C before use. Acarbose (0.01–0.1 mg/mL prepared in buffer) was included as the positive control. The assay was performed in 96-well flat-bottom plates using a multichannel automatic pipette (Eppendorf SE, Hamburg, Germany.). Initially, 20 mM sodium phosphate buffer (pH 6.9) was added to the wells, followed by 25 μL of the sample solution and 50 μL of α-amylase solution (0.8 U/mL in buffer). The reaction mixtures were incubated at 37 °C for 10 min. Subsequently, 50 μL of the prepared substrate solution was added, and the mixtures were incubated again at 37 °C for 10 min. The enzymatic reaction was terminated by adding 25 μL of 1 M HCl, after which 100 μL of the I_2_/KI reagent was added to each well. For the sample blanks, all assay components were included except the enzyme, which was replaced by 50 μL of buffer. Control wells contained all assay components with 25 μL of the solvent used to prepare the sample instead of the sample solution. Control incubations, corresponding to 100% α-amylase activity, were performed under identical conditions, with the plant extract replaced by its respective solvent. Absorbance was measured at 630 nm for both the samples and their corresponding blanks. The percentage inhibition of α-amylase activity (Inh %) was calculated according to the following equation:

$$\mathrm{Inh}\%=\left[\frac{\left({\mathrm{Abs}}_{\mathrm{control}}-{\mathrm{Abs}}_{\mathrm{control}\mathrm{blank}}\right)-\left({\mathrm{Abs}}_{\mathrm{sample}}-{\mathrm{Abs}}_{\mathrm{sample}\mathrm{blank}}\right)}{{\mathrm{Abs}}_{\mathrm{control}}-{\mathrm{Abs}}_{\mathrm{control}\mathrm{blank}}}\right]\times 100$$

where *Abs_control_*—absorbance of control, *Abs_sample_*—absorbance of sample, *Abs_control blank_*—absorbance of control blank, *Abs_sample blank_*—absorbance of sample blank.

### 4.6. Preclinical Evaluation

#### 4.6.1. Anti-Inflammatory Study

The anti-inflammatory activity of the tablet formulation was evaluated in adult male outbred mice weighing 23–25 g using a carrageenan-induced paw edema model [112]. Animals were allocated to six groups (*n* = 20 per group): vehicle control; *S. dumetorum* extract tablets at doses of 100, 250, and 500 mg/kg body weight referring to the equivalent amounts of *S. dumetorum* extract; and reference treatment groups receiving Rotokan (0.87 mL/kg) or diclofenac sodium (8 mg/kg).

The test and reference formulations were administered orally by gavage. Acute inflammation was induced by subplantar injection of 0.1 mL of 1% carrageenan suspension into the left hind paw. Paw volume was measured before carrageenan administration and at 3, 12, and 24 h after induction of inflammation. The anti-inflammatory effect was assessed based on the reduction in paw edema relative to the vehicle-treated control group.

#### 4.6.2. Repeated-Dose Toxicity and Immunotoxicity Studies

Repeated-dose toxicity and immunotoxicity of the tablet formulation were evaluated following previously established acute toxicity data. The formulation was administered orally by gavage once daily for 21 consecutive days at the doses specified in the experimental protocol, while control animals received the corresponding vehicle [113]. Throughout the treatment period, animals were monitored for general clinical condition, behavior, food and water consumption, and changes in body weight. At the end of the treatment period, animals were subjected to hematological, biochemical, immunological, organ-weight, and morphological/histopathological examinations. Potential immunotoxic effects were assessed based on changes in relevant immune-related parameters and the morphology of immune organs.

#### 4.6.3. Allergenicity and Skin Sensitization

The allergenic and sensitizing potential of the tablet formulation was evaluated in albino guinea pigs, a species commonly used for the assessment of dermal sensitization [114]. The studies included assessment of local irritant effects, epicutaneous sensitization, and sensitization under conditions of Freund’s complete adjuvant-induced immune stimulation. Guinea pigs of both sexes (6 females and 6 males per group) with a body weight of approximately 200–250 g were used. For the local irritation study, a suspension prepared from the tablet formulation corresponding to a dose of 250 mg/kg body weight referring to the equivalent amounts of *S. dumetorum* extract was applied once to a previously shaved 2 × 2 cm area of the lateral trunk, while control animals received physiological saline. Skin reactions were assessed visually at 1, 24, and 48 h after application using a standardized erythema and edema scoring scale. For the epicutaneous sensitization study, the tablet suspension was applied daily for 20 consecutive days at concentrations of 10% and 20% [113]. Skin reactions were monitored throughout the sensitization period, and challenge testing was performed after the 10th and 20th applications using a three-fold increased challenge dose. In a separate protocol, sensitization was induced using the tablet suspension administered subcutaneously in combination with Freund’s complete adjuvant (1:1), followed by challenge testing after 14 days. The occurrence, severity, and frequency of skin reactions were recorded according to the established scoring system.

#### 4.6.4. Reproductive and Embryofetal Toxicity

Reproductive study. The study was conducted in 50 sexually mature outbred white rats (30 females and 20 males), aged 3.5–4 months and weighing 220–240 g. Female rats were randomly allocated to three groups: an intact control group (*n* = 10), a vehicle control group receiving distilled water by oral gavage for 14 days (*n* = 10), and a treatment group receiving *S. dumetorum* tablets at 250 mg/kg body weight referring to the equivalent amounts of *S. dumetorum* extract by oral gavage for 14 days (*n* = 10). Twenty intact males were used for mating and assessment of female reproductive behavior and were not included in the experimental groups. Following treatment, female sexual behavior was assessed using a modified open-field test with zoosocial preference [115], including latency and duration of sexual activity, number of approaches to the male, and lordosis responses. The estrous cycle was monitored daily for 14 days by vaginal smear examination. Fertility was subsequently assessed by mating treated or control females with intact males for 10 days. At the end of the observation period, females were euthanized and reproductive organs were examined. Pregnancy rate, ovarian weight and relative ovarian weight, number of corpora lutea, implantation sites, live fetuses, and pre- and post-implantation losses were determined.

Embryo–fetal developmental study. The potential embryotoxic and fetotoxic effects of the tablets were evaluated in 30 sexually mature outbred female rats weighing 220–240 g. Animals were allocated to three groups (*n* = 10 per group): intact control, vehicle control receiving 0.4 mL of water by oral gavage, and treatment receiving *S. dumetorum* tablets at 250 mg/kg body weight referring to the equivalent amounts of *S. dumetorum* extract by oral gavage. Treatment was administered from gestational days 6 to 16. Maternal body weight and general clinical condition were monitored throughout pregnancy. On gestational day 20, females were euthanized and the uterus and ovaries were examined [111]. The numbers of corpora lutea, implantation sites, live and dead fetuses, and resorptions were recorded, and pre- and post-implantation losses were calculated. Fetuses were examined macroscopically, weighed, measured for crown–rump length, and sexed. Approximately one-third of fetuses from each litter were fixed in Bouin’s solution for visceral examination, while the remaining fetuses were fixed in 96% ethanol and processed for skeletal examination using alizarin staining.

### 4.7. Statistical Analysis

Statistical analyses were conducted using SigmaPlot software (version 12.0), Statistica 10.0 (StatSoft Inc., Tulsa, OK, USA), Microsoft Excel 2019 (Microsoft, Redmond, WA, USA), and GraphPad Prism (version 10.2, GraphPad Software, San Diego, CA, USA), as appropriate. Data obtained from inhibition assays were reported as mean ± standard error of the mean (SEM). The IC_50_ values for the DPPH assay were estimated by nonlinear regression analysis. The normality of the data distribution was evaluated using the Shapiro–Wilk test. Statistical procedures were selected according to the distribution characteristics of the data and the experimental design, with either parametric or non-parametric tests applied as appropriate. Quantitative results are presented as mean ± standard error or median with interquartile range, depending on data distribution. Differences were considered statistically significant at *p* < 0.05.

## 5. Conclusions

The present study provides an integrated phytochemical, biological, and preclinical evaluation of *S. dumetorum* and a tablet formulation developed from its hydroethanolic extract. The extract demonstrated a diverse phytochemical profile, including phenolic, flavonoid, diterpenoid, and organic acid constituents, together with pronounced antioxidant, α-amylase inhibitory, and broad-spectrum antimicrobial activities. Following incorporation into the tablet dosage form, measurable antioxidant and α-amylase inhibitory activities were retained, although their quantitative profiles differed from those of the original extract, highlighting the influence of the final formulation matrix on the measurable biological properties of the extract.

Comprehensive in vivo assessment of the tablet formulation revealed no treatment-related adverse effects under the tested conditions following repeated administration. No evidence of clinically relevant immunotoxicity, impairment of female reproductive function, embryofetal developmental toxicity, abnormalities in skeletal ossification, or sensitization was observed. The present findings therefore extend the previously established acute oral tolerability data and provide additional evidence supporting the favorable preclinical safety profile of the *S. dumetorum* tablet formulation.

Overall, these results support the continued pharmaceutical development of *S. dumetorum* tablets and provide a basis for further studies addressing their pharmacological efficacy, formulation performance, and longer-term safety.

## Figures and Tables

**Figure 1:**
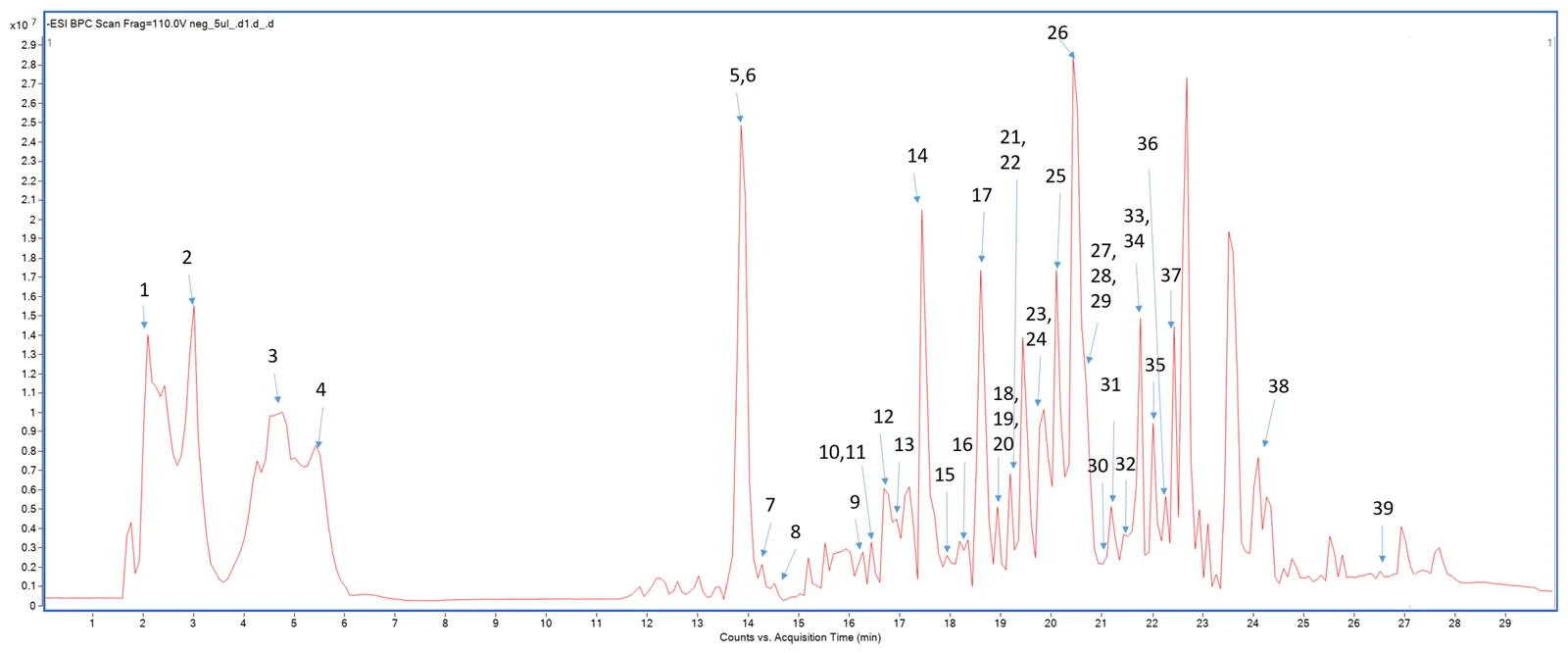
Base peak chromatogram (BPC) of the *S. dumetorum* extract (HPLC-ESI-QTOF-MS/MS).

**Table 1:** HPLC-ESI-QTOF-MS/MS analysis of the *S. dumetorum* extract in negative ionization mode.

№	RT (min)	Formula	Theoretical (*m*/*z*)	Experimental (*m*/*z*)	∆ ppm	DBE	MS/MS Fragments	Tentative Identification	Ref.	MSI Level
1	2.254	C_4_H_6_O_5_	133.0142	133.0135	4.83	2	71.0149 115.0034 133.0136	Malic acid	[13]	2
2	2.922	C_6_H_8_O_7_	191.0197	191.0209	−6.11	3	87.0100 111.0094 191.0195	Citric acid	[13]	2
3	4.589	C_4_H_4_O_4_	115.0037	115.0043	−5.33	3	71.0161 115.0045	Fumaric acid	[14]	2
4	5.489	C_4_H_6_O_4_	117.0193	117.0187	5.36	2	73.0305 99.0071 117.0187	Succinic acid	[15]	2
5	13.844	C_9_H_10_O_5_	197.0455	197.0443	6.3	5	72.9944 135.0450 179.0344	Danshensu	[15,16]	2
6	13.894	C_18_H_20_O_10_	395.0984	395.0982	0.43	9	72.9944 135.0449 197.0445 315.0749	Acetylisobiflorin	[13]	2
7	14.244	C_13_H_16_O_9_	315.0722	315.0713	2.7	6	71.0145 123.0444 153.0547 197.0466 255.0482	Gentisoyl glucoside	[13]	2
8	14.612	C_7_H_6_O_4_	153.0193	153.0187	4.1	5	64.9992 109.0274	Dihydroxybenzoic acid (gentisic acid)	[17]	2
9	16.229	C_7_H_6_O_3_	137.0244	137.0244	0.13	5	108.0223	Hydroxybenzoic Acid	[13]	2
10	16.479	C_15_H_18_O_8_	325.0929	325.0925	1.2	7	65.0399 119.0502 163.0403	p-Coumaric acid glucoside	[13]	2
11	16.579	C_27_H_28_O_18_	639.1203	639.1158	7.01	14	72.9938 179.0322 243.0617 297.0730 359.0721 495.1250	Quercetin glucosyl-glucuronide isomer 1	[13]	2
12	16.729	C_21_H_32_O_12_	475.1821	475.1827	−1.26	6	113.0250 161.0450 271.0995 329.1233 431.1381	Cistanoside E	[13]	2
13	17.180	C_15_H_14_O_7_	305.0667	305.0667	−0.08	9	96.9604 165.0896 225.1111	Epigallocatechin	[14]	2
14	17.59	C_9_H_8_O_4_	179.035	179.0330	11.01	6	107.0500 135.0435 179.0336	Caffeic acid	[13,14,15,16]	2
15	17.847	C_7_H_6_O_4_	153.0193	153.0186	4.75	5	65.0407 109.0299 153.0189	Protocatechuic acid	[14]	2
16	18.581	C_27_H_24_O_14_	571.1093	571.1043	8.79	16	72.9936 135.0434 197.0423 285.0735 329.0635 439.1351 527.1153	Yunnaneic acid E	[13]	2
17	18.748	C_27_H_30_O_15_	593.1512	593.1474	6.39	13	285.0368	Luteolin rutinoside derivative	[15]	2
18	18.931	C_27_H_24_O_12_	539.1195	539.1155	7.41	16	72.9935 179.0323 297.0734 359.0733 495.1291	Yunnaneic acid D Isomer 1	[13]	2
19	18.998	C_27_H_24_O_12_	539.1195	539.1148	8.7	16	72.9931 135.0441 197.0429 297.0734 359.0732 495.1273	Yunnaneic acid D Isomer 2	[13]	2
20	19.098	C_24_H_16_O_10_	463.0671	464.0674	−0.71	17	251.0449 347.0656 419.2094	Castillicetin	[13]	2
21	19.198	C_24_H_26_O_13_	521.1301	521.1282	3.57	12	359.0690	Salviaflaside	[15]	2
22	19.231	C_29_H_26_O_14_	597.125	597.1223	4.48	17	72.9926 135.0419 197.0401 311.0845 399.0622 447.1197	Yunnaneic acid F	[13]	2
23	20.015	C_21_H_18_O_11_	445.0776	445.0776	0.08	13	85.0297 113.0242 175.0234 242.0849 269.0441 311.0504 359.0766	Apigenin glucuronide	[15]	2
24	20.37	C_27_H_24_O_13_	555.1144	555.1158	−2.49	16	72.9939 135.0451 197.0447 269.0803 359.0769 493.1141	Salvianolic acid K	[13]	2
25	20.43	C_36_H_32_O_16_	719.1618	719.1645	−3.81	21	72.9939 161.0210 223.0211 359.0723	Sagerinic acid	[13]	2
26	20.732	C_27_H_22_O_12_	537.1016	537.1038	4.18	17	109.0274 161.0215 221.0412 295.0560 359.0713 449.1186 149.1053	Lithospermic acid A	[13]	2
27	20.84	C_27_H_22_O_12_	537.1038	537.1009	5.48	17	72.9927 135.0439 197.0436 295.0585 359.0734	Lithospermic acid B (Salvianolic acid B)	[15]	2
28	20.849	C_18_H_16_O_8_	359.0772	359.0750	6.22	11	72.9941 135.0450 161.0224 197.0430	Rosmarinic acid	[13,15,16]	2
29	20.93	C_25_H_22_O_10_	481.114	481.11	8.34	15	72.9931 137.0226 197.0432 257.0773 283.0575 343.0733	Silybin	[13]	2
30	21.066	C_7_H_6_O_3_	137.0244	137.0232	8.82	5	93.0329 137.0202	Protocatechuic aldehyde	[14,15]	2
31	21.35	C_18_H_34_O_5_	329.2233	329.2296	11.35	2	139.1105 211.1310 293.2087	Pinellic acid	[13]	2
32	21.566	C_20_H_28_O_5_	347.1864	347.1834	8.61	7	109.0614 191.1404 303.1911	Hydroxycarnosic acid	[15]	2
33	21.733	C_20_H_26_O_5_	345.1707	345.1682	7.36	8	301.1722	Rosmanol	[15]	2
34	21.967	C_16_H_12_O_6_	299.0561	299.0538	7.7	11	284.0286	Hispidulin	[15]	2
35	22.083	C_17_H_14_O_6_	313.0718	313.0692	8.16	11	283.0324 298	Cirsimaritin	[15]	2
36	22.417	C_16_H_12_O_5_	283.0612	283.0589	8.09	11	240.0391 268.0339	Genkwanin	[15,16]	2
37	22.434	C_16_H_12_O_5_	283.0612	283.062	−2.83	11	63.0234 117.0334 240.0428	Acacetin	[14]	2
38	24.335	C_18_H_32_O_3_	295.2256	295.2279	7.66	3	113.1012 195.1440 230.9900	Hydroxylinoleic acid	[13]	2
39	26.603	C_20_H_28_O_2_	299.2017	299.2032	−5.15	7	88.0415 187.9421 255.2445	Sugiol	[13]	2

RT—retention time; DBE—number of double bonds and rings; ∆ ppm—error of *m*/*z* measurement; the letters placed at the numbers of ions indicate the occurrence of the isomers with the same *m*/*z*.

**Table 2:** Total phenolic and flavonoid contents of *S. dumetorum* extract and tablets.

Sample Name	TFC	TPC
*S. dumetorum* extract	40.55 ± 5.56 mg QE/g_extr_	40.15 ± 2.25 mg GAE/g_extr_
Tablets	ND	38.91 ± 9.63 mg GAE/g_tablet_

QE—quercetin equivalent; GAE—gallic acid equivalent; ND—not detected.

**Table 3:** Antioxidant activity of *S. dumetorum* extract and tablets.

Sample Name	TEAC, mM	TAC	FRAP	DPPH IC_50_, mg/mL
*S. dumetorum* extract	1.88 ± 0.07	250.43 ± 12.08 mg AsAE/g_extr_	427.91 ± 9.74 mg TE/g_extr_	0.03 ± 0.00
Tablets	0.16 ± 0.00	69.57 ± 6.85 mg AsAE/g_tablet_	55.83 ± 1.92 mg TE/g_tablet_	>1.0

AsA: Ascorbic acid equivalent; TE: Trolox equivalent.

**Table 4:** α -Amylase enzyme inhibitory potential of *S. dumetorum* extract and tablets.

Sample Name	Result
*S. dumetorum* extract	5.63± 0.51 mg AcrE/g_extr_
Tablets	4.53± 0.18 mg AcrE/g_tablet_

AcrE: Acarbose equivalent.

**Table 5:** Antimicrobial activity of investigated *S. dumetorum* extract expressed as MICs and MBCs/MFCs (mg/mL).

Group	Microorganism (ATCC)	MIC	MBC/MFC	Control, mg/L
Gram-positive	*Staphylococcus aureus* (ATCC 25923)	1.25	2.5	0.98 ^1^
	*Staphylococcus aureus* (ATCC BA1707)	1.25	2.5	0.98 ^1^
	*Staphylococcus epidermidis* (ATCC 12228)	1.25	1.25	0.98 ^1^
	*Micrococcus luteus* (ATCC 10240)	1.25	2.5	0.12 ^1^
	*Bacillus cereus* (ATCC 10876)	2.5	2.5	0.98 ^1^
	*Enterococcus faecalis* (ATCC 29212)	5	10	1.95 ^1^
Gram-negative	*Salmonella* Typhimurium (ATCC 14028)	10	10	0.061 ^2^
	*Escherichia coli* (ATCC 25922)	10	10	0.015 ^2^
	*Proteus mirabilis* (ATCC 12453)	5	5	0.030 ^2^
	*Klebsiella pneumoniae* (ATCC 13883)	2.5	2.5	0.122 ^2^
	*Pseudomonas aeruginosa* (ATCC 9027)	10	10	0.488 ^2^
Yeast	*Candida glabrata* (ATCC 90030)	1.25	5	0.24 ^3^
	*Candida albicans* (ATCC 10231)	2.5	5	0.48 ^3^
	*Candida parapsilosis* (ATCC 22019)	5	5	0.24 ^3^

^1^ Vancomycin, ^2^ Ciprofloxacin, ^3^ Nystatin.

**Table 6:** Technical specification of the *S. dumetorum* extract.

Quality Attribute	Acceptance Criterion	Result
Appearance	Dry, concentrated powder; characteristic aromatic odor; bitter taste	Dry, dark green powder
Solubility	Soluble in water at 30 °C and in 90% ethanol	Soluble
Identification of phenolic compounds	Characteristic color reactions with potassium dichromate and ferric chloride, as specified in the analytical procedure	A yellow-brown coloration developed after the addition of potassium dichromate; a dark blue-black coloration developed after the addition of ferric chloride.
Rosmarinic acid content	-	6.092 ± 0.176%
Loss on drying	Not more than 5%	4.8%
Heavy metals	Not more than 0.01%	Meets the acceptance criterion
Microbiological purity	Category 4B, with microbial limits and absence criteria as specified in the applicable pharmacopoeial requirements	Meets the acceptance criterion

**Table 7:** Quality control of *S. dumetorum*-based tablets.

Quality Attribute	Acceptance Criterion	Result
Appearance	Light grey tablets with a uniform surface	Conforms
Mass uniformity	Maximum deviation within ± 10%	5%
Disintegration time	Not more than 15 min	11 min 47 s
Dissolution at 45 min	Not less than 75%	79%
Crushing strength	Not less than 50 N	107.2 N
Friability (mass retained)	At least 97%	99.991%
Rosmarinic acid content	-	0.41%

**Table 8:** Results of a comparative evaluation of the anti-inflammatory activity of tablets.

Sample	Paw Thickness, mm			
	0 h	3 h	12 h	24 h
Control	3.3 (2.8–3.4)	4.4 (4.2–4.5) ^a^	4.1 (3.7–4.3) ^a^	3.7 (3.6–4.1) ^a,β^
Tablets, 500 mg/kg	2.9 (2.6–3.1)	3.4 (3.3–3.4) *^,a^	3.2 (3.0–3.3) *	3.1 (2.9–3.4)*
Tablets, 250 mg/kg	3.2 (2.7–3.3)	3.5 (3.4–3.5) *^,a^	3.3 (2.9–3.3) *	3.2 (2.8–3.3) *^,β^
Tablets, 100 mg/kg	2.8 (2.7–3.2)	4.0 (3.6–4.0) *^,a,†^	3.8 (3.7–4.1) ^a,†,§^	3.3 (3.2–3.3) *^,β^
Rotokan	2.9 (2.8–3.5)	3.4 (3.3–4.0) *^,a^	3.0 (2.9–3.2) *	3.3 (3.1–3.4) *
Diclofenac sodium	2.8 (2.7–3.1)	3.3 (3.2–3.5) *^,a^	3.2 (3.0–3.4) *	3.1 (3.0–3.3) *

* *p* < 0.05—vs. control at the corresponding time point; ^a^ *p* < 0.05—0 h vs. 3 h, 12 h, and 24 h; ^β^ *p* < 0.05—3 h vs. 24 h; ^†^ *p* < 0.05—*S. dumetorum* extract-based tablets vs. diclofenac sodium; ^§^ *p* < 0.05—*S. dumetorum* extract-based tablets vs. Rotokan; *n* = 20.

**Table 9:** Effects of repeated intragastric administration of *S. dumetorum* tablets on body weight, food intake, and water consumption in mice during the treatment and recovery periods.

Parameter	Sex	Period	Intact Control	Vehicle Control	100 mg/kg	250 mg/kg	1000 mg/kg
Body weight, g	Male	Baseline	22.8 ± 1.3	22.2 ± 1.4	22.1 ± 1.5	22.5 ± 1.4	22.4 ± 1.4
		Week 1	23.7 ± 1.2	23.8 ± 1.3	23.9 ± 1.3	23.4 ± 1.3	23.6 ± 1.1
		Week 2	24.5 ± 1.4	24.9 ± 1.3	24.4 ± 1.1	24.1 ± 1.5	24.7 ± 1.2
		Week 3	24.9 ± 1.1	24.8 ± 1.2	24.7 ± 1.3	24.4 ± 1.4	24.1 ± 1.3
		Recovery week 3	25.3 ± 1.3	25.1 ± 1.4	25.8 ± 1.2	25.7 ± 1.2	24.2 ± 1.4
	Female	Baseline	21.7 ± 1.4	21.3 ± 1.2	21.8 ± 1.4	21.3 ± 1.5	21.7 ± 1.3
		Week 1	22.9 ± 1.3	23.4 ± 1.4	22.7 ± 1.5	22.8 ± 1.2	22.7 ± 1.4
		Week 2	24.3 ± 1.2	24.1 ± 1.5	24.3 ± 1.2	24.3 ± 1.3	24.2 ± 1.1
		Week 3	24.6 ± 1.3	24.1 ± 1.3	24.7 ± 1.4	24.7 ± 1.1	24.5 ± 1.4
		Recovery week 3	25.4 ± 1.5	25.4 ± 1.1	25.7 ± 1.3	25.6 ± 1.4	25.7 ± 1.3
Food intake, g/100 g bw	Male	Week 3	13.6 ± 0.43	13.1 ± 0.53	14.1 ± 0.53	13.2 ± 0.56	12.8 ± 0.51
		Recovery week 3	16.5 ± 0.28	16.2 ± 0.70	16.7 ± 0.73	16.1 ± 0.61	16.4 ± 0.87
	Female	Week 3	12.6 ± 0.41	12.7 ± 0.26	12.4 ± 0.27	12.2 ± 0.34	12.1 ± 0.31
		Recovery week 3	13.3 ± 0.86	13.2 ± 0.93	13.1 ± 0.84	13.5 ± 0.29	13.6 ± 0.58
Water consumption, mL/100 g bw	Male	Week 3	16.9 ± 0.41	17.4 ± 0.38	17.2 ± 0.46	17.4 ± 0.62	17.3 ± 0.36
		Recovery week 3	17.1 ± 0.54	17.6 ± 0.45	17.4 ± 0.40	17.7 ± 0.30	17.2 ± 0.36
	Female	Week 3	18.0 ± 0.36	18.6 ± 0.21	18.9 ± 0.27	18.3 ± 0.20	18.1 ± 0.20
		Recovery week 3	18.4 ± 0.22	18.8 ± 0.56	18.6 ± 0.40	18.7 ± 0.48	18.4 ± 0.53

Values are presented as mean ± SEM. No statistically significant differences were observed between treated and control groups (*p* > 0.05). Food and water consumption were normalized to 100 g body weight. “Week 3” indicates the end of the treatment period, whereas “Recovery week 3” indicates three weeks after treatment discontinuation.

**Table 10:** Effects of repeated administration of *S. dumetorum* tablets on open-field behavioral activity in mice during the treatment and recovery periods.

Behavioral Parameter	Sex	Period	Intact Control	Vehicle Control	100 mg/kg	250 mg/kg	1000 mg/kg
Horizontal activity	Male	Treatment	29.3 ± 1.66	32.2 ± 2.11	34.9 ± 3.43	29.6 ± 2.21	27.2 ± 4.20
	Female		44.8 ± 4.39	46.7 ± 4.53	48.6 ± 4.21	46.4 ± 3.26	48.7 ± 3.08
	Male	Recovery	23.0 ± 1.74	20.4 ± 1.92	24.5 ± 1.59	22.8 ± 2.30	22.4 ± 2.24
	Female		45.3 ± 2.86	42.0 ± 3.57	41.2 ± 3.67	37.4 ± 2.20	43.3 ± 3.32
Vertical activity	Male	Treatment	8.7 ± 1.09	9.3 ± 1.17	12.2 ± 1.11	8.7 ± 0.93	8.6 ± 1.59
	Female		13.7 ± 1.10	15.3 ± 1.88	13.4 ± 1.81	16.9 ± 1.64	15.2 ± 2.32
	Male	Recovery	6.4 ± 0.70	7.5 ± 0.81	6.7 ± 0.82	7.7 ± 1.08	6.5 ± 0.90
	Female		11.8 ± 1.09	11.3 ± 1.33	9.0 ± 1.37	12.0 ± 1.43	11.0 ± 1.03
Burrowing activity	Male	Treatment	4.5 ± 0.62	4.6 ± 1.03	2.1 ± 0.55	4.9 ± 1.12	2.8 ± 0.57
	Female		5.4 ± 1.19	3.0 ± 1.00	3.0 ± 0.61	4.4 ± 0.31	4.1 ± 0.74
	Male	Recovery	3.0 ± 0.47	1.8 ± 0.33	1.5 ± 0.27	1.8 ± 0.49	2.1 ± 0.57
	Female		2.0 ± 0.54	2.5 ± 0.67	2.3 ± 0.65	3.5 ± 0.87	3.3 ± 0.80
Grooming	Male	Treatment	4.5 ± 0.27	4.2 ± 0.42	5.1 ± 0.77	3.1 ± 0.41	5.1 ± 0.74
	Female		8.0 ± 0.76	7.0 ± 0.75	5.6 ± 0.31	5.1 ± 0.41	6.1 ± 0.53
	Male	Recovery	4.4 ± 0.34	4.3 ± 0.73	4.7 ± 0.65	3.2 ± 0.68	3.8 ± 0.76
	Female		4.4 ± 0.58	4.5 ± 0.64	3.7 ± 0.65	3.6 ± 0.70	4.2 ± 0.57
Defecation	Male	Treatment	4.1 ± 0.46	5.1 ± 0.43	3.3 ± 0.75	3.3 ± 0.83	3.6 ± 0.62
	Female		3.0 ± 0.77	2.6 ± 0.54	2.5 ± 1.02	4.0 ± 0.47	1.7 ± 0.37
	Male	Recovery	4.1 ± 0.53	3.3 ± 0.90	3.0 ± 0.83	3.6 ± 0.96	2.5 ± 0.65
	Female		2.1 ± 0.59	1.1 ± 0.41	3.0 ± 0.82	1.3 ± 0.96	1.8 ± 0.53
Center entries	Male	Treatment	0.7 ± 0.26	0.5 ± 0.22	0.2 ± 0.13	0.4 ± 0.16	0.2 ± 0.13
	Female		0.5 ± 0.17	0.7 ± 0.30	0.5 ± 0.17	0.9 ± 0.31	0.9 ± 0.28
	Male	Recovery	0.5 ± 0.22	0.3 ± 0.15	0.4 ± 0.16	0.4 ± 0.16	0.4 ± 0.16
	Female		0.1 ± 0.10	0.2 ± 0.13	0.1 ± 0.11	0.6 ± 0.16	0.2 ± 0.13
Time to leave center, s	Male	Treatment	3.3 ± 0.97	3.5 ± 1.05	2.5 ± 0.79	4.2 ± 0.76	5.3 ± 0.91
	Female		2.2 ± 0.98	2.8 ± 0.57	4.1 ± 1.19	4.7 ± 1.41	3.7 ± 0.50
	Male	Recovery	10.0 ± 3.18	8.6 ± 1.28	8.9 ± 0.84	9.2 ± 2.98	10.2 ± 3.88
	Female		3.9 ± 1.02	2.8 ± 1.16	3.2 ± 1.15	6.2 ± 1.15	5.8 ± 1.36

Values are presented as mean ± SEM. Behavioral parameters are expressed as acts per animal, unless otherwise indicated. No statistically significant differences were observed between treated and control groups (*p* > 0.05). “Treatment” refers to the end of the three-week administration period, whereas “Recovery” refers to three weeks after treatment discontinuation.

**Table 11:** Effects of repeated administration of *S. dumetorum* tablets on major hematological parameters in mice during the treatment and recovery periods.

Parameter	Sex	Period	Intact Control	Vehicle Control	100 mg/kg	250 mg/kg	1000 mg/kg
Erythrocytes, ×10^12^/L	Male	Treatment	8.1 ± 0.30	8.4 ± 0.26	7.7 ± 0.14	8.5 ± 0.22	7.9 ± 0.49
	Female		7.7 ± 0.40	8.2 ± 0.40	8.1 ± 0.11	8.2 ± 0.11	8.5 ± 0.22
	Male	Recovery	8.4 ± 0.27	8.0 ± 0.25	8.3 ± 0.30	8.3 ± 0.27	8.4 ± 0.32
	Female		8.0 ± 0.17	7.8 ± 0.66	7.6 ± 0.64	7.4 ± 0.30	8.1 ± 0.44
Hemoglobin, g/L	Male	Treatment	139.7 ± 0.88	137.7 ± 3.71	133.3 ± 2.86	132.0 ± 1.53	131.0 ± 6.81
	Female		142.3 ± 3.48	139.7 ± 3.84	132.0 ± 1.53	149.0 ± 3.06	147.0 ± 7.21
	Male	Recovery	128.3 ± 3.76	131.7 ± 3.28	130.0 ± 5.29	131.0 ± 2.52	139.0 ± 4.93
	Female		128.0 ± 4.93	115.0 ± 7.55	115.0 ± 5.00	115.0 ± 5.00	124.0 ± 2.89
Hematocrit, L/L	Male	Treatment	42.7 ± 0.69	43.3 ± 0.93	42.9 ± 0.98	42.9 ± 0.52	41.9 ± 1.29
	Female		43.0 ± 1.72	42.8 ± 0.90	43.4 ± 0.62	45.1 ± 1.36	46.4 ± 4.16
	Male	Recovery	33.2 ± 1.11	35.7 ± 1.35	35.2 ± 1.91	36.2 ± 1.94	36.2 ± 1.28
	Female		38.1 ± 1.15	32.2 ± 2.76	35.3 ± 1.06	34.0 ± 1.00	35.4 ± 1.83
Platelets, ×10^9^/L	Male	Treatment	598.3 ± 37.20	605.7 ± 46.40	637.0 ± 21.55	522.7 ± 61.50	608.3 ± 10.73
	Female		575.3 ± 42.98	568.0 ± 70.53	547.7 ± 50.11	477.0 ± 14.57	582.7 ± 38.68
	Male	Recovery	607.0 ± 49.43	611.3 ± 34.83	651.0 ± 20.79	534.0 ± 53.59	575.0 ± 15.52
	Female		533.7 ± 7.36	573.3 ± 17.19	565.3 ± 27.72	542.3 ± 16.75	565.3 ± 27.72
Leukocytes, ×10^9^/L	Male	Treatment	11.3 ± 1.49	11.1 ± 0.77	12.1 ± 1.21	12.5 ± 1.91	11.7 ± 0.95
	Female		8.4 ± 0.38	8.0 ± 0.75	9.7 ± 1.35	14.5 ± 1.56	9.0 ± 0.49
	Male	Recovery	14.8 ± 1.13	14.6 ± 1.85	15.3 ± 2.36	15.6 ± 1.89	13.3 ± 1.13
	Female		7.3 ± 1.56	7.6 ± 1.21	8.8 ± 1.23	8.8 ± 0.75	8.7 ± 0.78
ESR, mm/h	Male	Treatment	2.3 ± 0.33	1.7 ± 0.33	2.3 ± 0.88	2.7 ± 0.67	1.3 ± 0.33
	Female		1.0 ± 0.00	1.3 ± 0.33	1.3 ± 0.33	1.0 ± 0.00	1.0 ± 0.00
	Male	Recovery	1.7 ± 0.33	1.3 ± 0.33	1.3 ± 0.33	1.7 ± 0.67	1.7 ± 0.33
	Female		1.3 ± 0.33	1.7 ± 0.33	2.0 ± 0.00	1.7 ± 0.33	1.3 ± 0.33

Values are presented as mean ± SEM. Treatment indicates the end of the three-week administration period, whereas recovery indicates three weeks after treatment discontinuation. No statistically significant differences were observed between treated and control groups (*p* > 0.05). Detailed leukocyte differential counts, clotting time, color index, and phagocytic index are provided in Supplementary Table S1.

**Table 12:** Effects of repeated administration of *S. dumetorum* tablets on selected plasma biochemical parameters in mice.

Parameter	Sex	Period	Intact Control	Vehicle Control	100 mg/kg	250 mg/kg	1000 mg/kg
Glucose, mmol/L	Male	Treatment	6.16 ± 0.68	6.25 ± 0.81	7.51 ± 0.59	5.75 ± 0.53	5.35 ± 0.28
	Female		6.08 ± 0.63	6.55 ± 0.73	6.66 ± 0.65	7.09 ± 0.49	5.59 ± 0.45
	Male	Recovery	5.05 ± 0.47	5.65 ± 0.44	5.03 ± 0.33	5.11 ± 0.36	4.66 ± 0.27
	Female		4.73 ± 0.44	5.28 ± 0.15	4.79 ± 0.35	4.97 ± 0.31	4.94 ± 0.26
Total protein, g/L	Male	Treatment	82.75 ± 11.00	78.72 ± 10.52	81.32 ± 10.85	83.17 ± 7.29	81.45 ± 2.81
	Female		108.64 ± 3.62	96.62 ± 4.33	100.83 ± 2.98	94.38 ± 10.58	102.79 ± 10.91
	Male	Recovery	72.25 ± 1.42	67.99 ± 1.71	71.03 ± 2.81	67.57 ± 0.53	74.92 ± 2.35
	Female		71.34 ± 1.47	75.59 ± 4.44	71.58 ± 1.13	72.19 ± 2.96	70.61 ± 2.69
Creatinine, μmol/L	Male	Treatment	207.11 ± 30.55	205.27 ± 18.22	202.81 ± 8.06	179.46 ± 2.66	186.83 ± 9.14
	Female		165.32 ± 10.54	189.91 ± 11.98	200.97 ± 15.10	199.74 ± 5.71	171.47 ± 9.33
	Male	Recovery	156.98 ± 3.99	158.04 ± 6.72	161.72 ± 7.06	171.21 ± 7.17	155.40 ± 3.37
	Female		173.31 ± 1.80	173.31 ± 5.40	170.15 ± 3.03	174.89 ± 7.25	172.26 ± 3.98
Urea, mmol/L	Male	Treatment	9.06 ± 0.46	10.31 ± 0.67	9.44 ± 0.84	11.93 ± 1.38	13.06 ± 1.58
	Female		9.77 ± 0.69	11.32 ± 1.28	12.38 ± 1.62	10.35 ± 0.99	10.97 ± 1.23
	Male	Recovery	6.40 ± 0.21	6.27 ± 0.31	5.43 ± 0.12	5.29 ± 0.36	6.12 ± 0.48
	Female		6.18 ± 0.20	6.57 ± 0.39	7.09 ± 0.73	6.16 ± 0.64	5.04 ± 0.68
Alkaline phosphatase, μkat/L	Male	Treatment	3.19 ± 0.39	2.91 ± 0.50	3.55 ± 0.24	3.18 ± 0.28	2.33 ± 0.08
	Female		4.34 ± 0.88	5.01 ± 0.41	5.62 ± 1.26	3.97 ± 0.58	6.75 ± 0.97
	Male	Recovery	2.87 ± 0.62	3.54 ± 0.40	2.72 ± 0.42	2.83 ± 0.25	2.31 ± 0.37
	Female		3.44 ± 0.16	3.78 ± 0.65	3.10 ± 0.28	3.39 ± 0.64	3.54 ± 0.35
ALT, μkat/L	Male	Treatment	0.41 ± 0.03	0.50 ± 0.05	0.48 ± 0.02	0.51 ± 0.07	0.42 ± 0.04
	Female		0.57 ± 0.02	0.54 ± 0.05	0.54 ± 0.05	0.57 ± 0.08	0.49 ± 0.04
	Male	Recovery	0.34 ± 0.02	0.36 ± 0.02	0.31 ± 0.03	0.26 ± 0.04	0.33 ± 0.03
	Female		0.35 ± 0.01	0.41 ± 0.03	0.37 ± 0.03	0.35 ± 0.05	0.39 ± 0.03
AST, μkat/L	Male	Treatment	0.67 ± 0.03	0.74 ± 0.04	0.70 ± 0.05	0.69 ± 0.05	0.74 ± 0.05
	Female		0.90 ± 0.06	0.84 ± 0.08	0.81 ± 0.08	0.76 ± 0.05	0.72 ± 0.05
	Male	Recovery	0.69 ± 0.01	0.73 ± 0.02	0.73 ± 0.04	0.70 ± 0.03	0.67 ± 0.03
	Female		0.77 ± 0.04	0.76 ± 0.08	0.74 ± 0.02	0.77 ± 0.05	0.72 ± 0.03
Total cholesterol, mmol/L	Male	Treatment	1.95 ± 0.27	2.63 ± 0.41	2.64 ± 0.27	2.44 ± 0.29	2.86 ± 0.24
	Female		2.21 ± 0.30	2.58 ± 0.40	2.52 ± 0.36	1.77 ± 0.27	2.04 ± 0.08
	Male	Recovery	1.96 ± 0.16	1.86 ± 0.13	2.06 ± 0.07	2.04 ± 0.15	2.06 ± 0.08
	Female		1.98 ± 0.05	1.91 ± 0.11	2.25 ± 0.11	2.19 ± 0.24	2.20 ± 0.12

Values are presented as mean ± SEM. Treatment indicates the end of the three-week administration period, whereas recovery indicates three weeks after treatment discontinuation. ALT, alanine aminotransferase; AST, aspartate aminotransferase. No statistically significant differences were observed between treated and control groups (*p* > 0.05). Detailed biochemical and immunological parameters, including antibody titers and IgG levels, are provided in Supplementary Table S2.

**Table 13:** Effects of 3-week oral administration of *S. dumetorum* tablets on relative organ weights and lymphoid organ cellularity in mice (M ± m).

Sex	Parameter	Control	Vehicle Control	100 mg/kg	250 mg/kg	1000 mg/kg
Males	Liver, %	3.1 ± 0.21	3.2 ± 0.06	3.4 ± 0.10	3.0 ± 0.07	3.0 ± 0.14
	Kidney, %	0.6 ± 0.03	0.6 ± 0.02	0.6 ± 0.01	0.6 ± 0.03	0.6 ± 0.01
	Adrenal glands, %	0.02 ± 0.002	0.02 ± 0.003	0.02 ± 0.001	0.02 ± 0.002	0.02 ± 0.002
	Thymus, %	0.1 ± 0.01	0.1 ± 0.02	0.1 ± 0.01	0.1 ± 0.01	0.1 ± 0.01
	Thymus cellularity, ×10^6^	36.8 ± 3.7	35.4 ± 3.7	33.4 ± 4.2	39.1 ± 2.4	37.1 ± 3.7
	Spleen, %	0.5 ± 0.04	0.4 ± 0.02	0.5 ± 0.07	0.5 ± 0.03	0.4 ± 0.04
	Spleen cellularity, ×10^6^	101.5 ± 6.2	103.7 ± 4.3	102.9 ± 7.1	106.7 ± 6.3	104.5 ± 5.2
Females	Liver, %	3.6 ± 0.21	3.7 ± 0.06	3.5 ± 0.09	3.6 ± 0.22	3.6 ± 0.20
	Kidney, %	0.6 ± 0.01	0.6 ± 0.02	0.6 ± 0.02	0.6 ± 0.05	0.6 ± 0.01
	Adrenal glands, %	0.03 ± 0.002	0.02 ± 0.003	0.03 ± 0.003	0.02 ± 0.003	0.03 ± 0.003
	Thymus, %	0.2 ± 0.02	0.2 ± 0.01	0.2 ± 0.004	0.2 ± 0.02	0.2 ± 0.02
	Thymus cellularity, ×10^6^	37.6 ± 4.2	35.4 ± 5.1	34.8 ± 2.6	36.5 ± 3.9	34.3 ± 4.7
	Spleen, %	0.5 ± 0.01	0.5 ± 0.02	0.5 ± 0.05	0.4 ± 0.05	0.6 ± 0.02
	Spleen cellularity, ×10^6^	105.4 ± 3.8	104.3 ± 5.1	105.9 ± 5.1	104.8 ± 6.3	103.7 ± 5.6

**Table 14:** Effects of *S. dumetorum* tablets on sexual behavior, fertility, and embryo–fetal development in rats.

Parameter	Control 1 (Intact)	Control 2 (Injection Water)	Tablets, 250 mg/kg
Sexual behavior			
Latency period, s	52.6 ± 3.54	53.7 ± 4.92	52.9 ± 3.54
Sexual activity, min	5.6 ± 0.13	5.8 ± 0.24	5.6 ± 0.34
Emotional approaches to male	10.2 ± 0.56	11.4 ± 0.61	10.3 ± 0.60
Lordosis responses	1.5 ± 0.67	1.4 ± 0.43	1.6 ± 0.26
Fertility and conception parameters			
Embryonic age, days	12.3 ± 0.80	13.2 ± 0.59	12.5 ± 0.73
Ovarian relative mass, g	0.05 ± 0.002	0.05 ± 0.003	0.05 ± 0.002
Corpora lutea, *n*	11.3 ± 0.57	11.3 ± 0.75	11.1 ± 0.69
Fetuses, *n*	10.0 ± 0.58	9.6 ± 0.60	9.6 ± 0.94
Pregnancy index, %	64.3 ± 9.22	64.3 ± 9.22	75.0 ± 9.45
Resorptions, %	0.3 ± 0.18	0.7 ± 0.29	0.4 ± 0.18
Preimplantation loss, %	8.4 ± 3.79	9.1 ± 2.98	9.4 ± 5.93
Postimplantation loss, %	2.9 ± 1.88	3.3 ± 2.72	3.5 ± 1.73
Embryo–fetal development			
Corpora lutea, *n*	11.7 ± 0.52	11.1 ± 0.84	10.5 ± 0.86
Implantation sites, *n*	8.3 ± 0.40	8.9 ± 1.08	8.3 ± 0.62
Resorptions, *n*	0.3 ± 0.10	0.3 ± 0.15	0.3 ± 0.27
Preimplantation loss, %	20.2 ± 2.37	19.5 ± 9.04	20.2 ± 3.81
Postimplantation loss, %	1.3 ± 1.25	1.3 ± 1.10	1.2 ± 1.01
Number of fetuses, *n*	9.2 ± 0.44	8.6 ± 1.13	8.8 ± 0.53
Cranio-caudal length, cm	3.4 ± 0.05	3.4 ± 0.05	3.4 ± 0.03
Fetal body weight, g	2.5 ± 0.05	2.5 ± 0.07	2.7 ± 0.12

Data are presented as mean ± SEM. No statistically significant differences were observed between the treated and control groups (*p* > 0.05). For the sexual behavior and fertility assessments, *S. dumetorum* tablets were administered intragastrically at 250 mg/kg for 2 weeks. For the embryo–fetal development assessment, tablets were administered intragastrically at 250 mg/kg during organogenesis (gestational days 6–16).

**Table 15:** Local irritant potential of *S. dumetorum* tablets following a single dermal application in guinea pigs.

Group	Dose	1 Day	2 Days	3 Days	4 Days	5 Days	6 Days	7 Days	8 Days	9 Days	10 Days
Control	—	0/10	0/10	0/10	0/10	0/10	0/10	0/10	0/10	0/10	0/10
*S. dumetorum* tablets	250 mg/kg	0/10	0/10	0/10	0/10	0/10	0/10	0/10	0/10	0/10	0/10

Data are presented as the number of animals with a positive skin reaction/total number of animals. Skin reactions were scored according to the S. V. Suvorov and V. V. Chernyshova scale.

**Table 16:** Sensitizing potential of *S. dumetorum* tablets following repeated epicutaneous application in guinea pigs.

Group	Test Substance	Dose	10 Applications			20 Applications		
			1 h	24 h	48 h	1 h	24 h	48 h
Control	Physiological saline	—	0/10	0/10	0/10	0/10	0/10	0/10
*S. dumetorum* tablets	Tablet suspension	250 mg/kg (20% solution)	0/10	0/10	0/10	0/10	0/10	0/10

Data are presented as the number of animals with a positive skin reaction/total number of animals. Skin reactions were scored according to the S. V. Suvorov and V. V. Chernyshova scale.

**Table 17:** Effect of *S. dumetorum* tablets on humoral immune parameters in mice after 3 weeks of intragastric administration.

Parameter	Control, Intact	Vehicle Control	100 mg/kg	1000 mg/kg	250 mg/kg
Male mice					
TNF-α, pg/mL	4.76 ± 2.3	4.38 ± 1.9	4.56 ± 2.7	4.65 ± 2.4	4.96 ± 1.8
IL-6, pg/mL	13.8 ± 3.7	12.6 ± 2.8	14.6 ± 1.8	13.7 ± 2.9	15.7 ± 3.6
C-reactive protein, μg/mL	1.76 ± 0.9	1.68 ± 0.7	1.84 ± 1.1	1.53 ± 0.6	1.47 ± 0.9
Ferritin, ng/mL	47.9 ± 9.8	46.8 ± 7.2	50.6 ± 4.3	48.7 ± 7.7	46.3 ± 6.9
Total antibody titer, log_2_	5.71 ± 0.81	5.86 ± 1.12	5.74 ± 0.91	5.69 ± 1.13	5.72 ± 1.02
IgG, log_2_	5.01 ± 1.12	4.89 ± 1.07	5.09 ± 1.07	4.98 ± 1.01	4.96 ± 1.05
Female mice					
TNF-α, pg/mL	4.81 ± 1.9	4.57 ± 1.7	4.43 ± 3.9	4.72 ± 3.1	4.93 ± 2.2
IL-6, pg/mL	13.7 ± 3.4	13.1 ± 3.1	15.4 ± 1.7	12.9 ± 3.8	14.8 ± 2.7
C-reactive protein, μg/mL	1.84 ± 0.7	1.73 ± 1.0	1.83 ± 1.4	1.62 ± 1.1	1.39 ± 0.8
Ferritin, ng/mL	46.7 ± 8.7	47.7 ± 9.1	49.4 ± 6.2	49.4 ± 7.9	45.4 ± 7.1
Total antibody titer, log_2_	4.76 ± 0.85	4.96 ± 1.13	5.04 ± 1.79	5.17 ± 1.15	5.16 ± 1.13
IgG, log_2_	5.13 ± 1.14	4.74 ± 1.08	4.78 ± 1.04	4.94 ± 1.05	4.97 ± 1.06

Data are presented as mean ± SEM. *p* > 0.05, treated groups vs. control groups.

**Table 18:** Humoral immune parameters in mice 3 weeks after discontinuation of *S. dumetorum* tablets.

Parameter	Control, Intact	Vehicle Control	100 mg/kg	1000 mg/kg	250 mg/kg
Male mice					
TNF-α, pg/mL	4.71 ± 1.6	4.49 ± 1.9	4.37 ± 3.2	4.81 ± 3.9	4.72 ± 2.4
IL-6, pg/mL	13.9 ± 4.1	13.8 ± 3.7	14.5 ± 2.6	13.5 ± 4.1	14.6 ± 2.1
C-reactive protein, μg/mL	1.79 ± 1.1	1.69 ± 0.8	1.76 ± 1.2	1.93 ± 1.0	1.48 ± 0.9
Ferritin, ng/mL	45.8 ± 9.1	48.4 ± 7.9	47.6 ± 6.9	48.3 ± 7.1	46.2 ± 8.3
Total antibody titer, log_2_	5.78 ± 0.74	5.47 ± 1.19	5.47 ± 1.34	5.47 ± 1.09	5.43 ± 1.13
IgG, log_2_	5.06 ± 1.09	5.08 ± 1.13	5.04 ± 1.04	4.73 ± 1.24	4.79 ± 1.19
Female mice					
TNF-α, pg/mL	4.65 ± 1.4	4.51 ± 1.7	4.41 ± 3.8	4.73 ± 3.4	4.76 ± 2.7
IL-6, pg/mL	14.7 ± 3.9	13.6 ± 4.1	12.9 ± 4.7	13.6 ± 3.9	14.1 ± 3.2
C-reactive protein, μg/mL	1.81 ± 1.0	1.61 ± 0.7	1.73 ± 1.0	1.87 ± 0.9	1.45 ± 1.1
Ferritin, ng/mL	45.7 ± 8.4	46.1 ± 7.7	49.4 ± 8.8	46.5 ± 6.7	45.4 ± 6.8
Total antibody titer, log_2_	4.83 ± 0.84	4.87 ± 1.15	5.12 ± 1.63	5.24 ± 1.18	5.32 ± 1.09
IgG, log_2_	5.07 ± 1.18	4.73 ± 1.16	4.65 ± 1.03	4.83 ± 1.09	4.96 ± 1.12

Data are presented as mean ± SEM. *p* > 0.05, treated groups vs. control groups.

## Data Availability

The original contributions presented in this study are included in the article/Supplementary Materials. Further inquiries can be directed to the corresponding author.

## Supplementary Materials

The following supporting information can be downloaded at: https://www.mdpi.com/article/10.3390/molecules31213802/s1, Figure S1: MS/MS fragmentation spectrum of malic acid in negative mode; Figure S2: MS/MS fragmentation spectrum of citric acid in negative mode; Figure S3: MS/MS fragmentation spectrum of fumaric acid in negative mode; Figure S4: MS/MS fragmentation spectrum of succinic acid in negative mode; Figure S5: MS/MS fragmentation spectrum of danshensu in negative mode; Figure S6: MS/MS fragmentation spectrum of acetylisobisflorin in negative mode; Figure S7: MS/MS fragmentation spectrum of gentioysil glucoside in negative mode; Figure S8: MS/MS fragmentation spectrum of dihydroxybenzoic acid (gentisic acid) in negative mode; Figure S9: MS/MS fragmentation spectrum of hydroxybenzoic acid in negative mode; Figure S10: MS/MS fragmentation spectrum of p-Coumaric acid glucoside in negative mode; Figure S11: MS/MS fragmentation spectrum of quercetin glucosyl-glucuronide isomer 1 in negative mode; Figure S12: MS/MS fragmentation spectrum of cistanoside E in negative mode; Figure S13: MS/MS fragmentation spectrum of epigallocatechin in negative mode; Figure S14: MS/MS fragmentation spectrum of caffeic acid in negative mode; Figure S15: MS/MS fragmentation spectrum of protocatechuic acid in negative mode; Figure S16: MS/MS fragmentation spectrum of yunnaneic acid E in negative mode; Figure S17: MS/MS fragmentation spectrum of luteolin rutinoside derivative in negative mode; Figure S18: MS/MS fragmentation spectrum of yunnaneic acid D isomer 1 in negative mode; Figure S19: MS/MS fragmentation spectrum of yunnaneic acid D isomer 2 in negative mode; Figure S20: MS/MS fragmentation spectrum of castilicetin in negative mode; Figure S21: MS/MS fragmentation spectrum of salviaflaside in negative mode; Figure S22: MS/MS fragmentation spectrum of yunnaneic acid F in negative mode; Figure S23: MS/MS fragmentation spectrum of apigenin glucuronide in negative mode; Figure S24: MS/MS fragmentation spectrum of salvianolic acid K in negative mode; Figure S25: MS/MS fragmentation spectrum of sagerinic acid in negative mode; Figure S26: MS/MS fragmentation spectrum of lithospermic acid A in negative mode; Figure S27: MS/MS fragmentation spectrum of lithospermic acid B (salvianolic acid B) in negative mode; Figure S28: MS/MS fragmentation spectrum of rosmarinic acid in negative mode; Figure S29: MS/MS fragmentation spectrum of silybin in negative mode; Figure S30: MS/MS fragmentation spectrum of protocatechuic aldehyde in negative mode; Figure S31: MS/MS fragmentation spectrum of pinellic acid in negative mode; Figure S32: MS/MS fragmentation spectrum of hydroxycarnosic acid in negative mode; Figure S33: MS/MS fragmentation spectrum of rosmanol in negative mode; Figure S34: MS/MS fragmentation spectrum of hispidulin in negative mode; Figure S35: MS/MS fragmentation spectrum of cirsimaritin in negative mode; Figure S36: MS/MS fragmentation spectrum of genkwanin in negative mode; Figure S37: MS/MS fragmentation spectrum of acacetin in negative mode; Figure S38: MS/MS fragmentation spectrum of hydroxylinoleic acid in negative mode; Figure S39: MS/MS fragmentation spectrum of sugiol in negative mode; Figure S40: Gallic acid calibration curve; Figure S41: Quercetin calibration curve; Figure S42: Calibration curve for Trolox; Figure S43: Calibration curve obtained with ascorbic acid; Figure S44: Calibration curve for Trolox. Table S1: Detailed hematological parameters of mice following three weeks of repeated administration of *Salvia dumetorum* tablets and after a three-week recovery period; Table S2: Detailed plasma biochemical and immunological parameters in mice following repeated administration of *Salvia dumetorum* tablets and after the recovery period; Table S3: Effects of 3-week oral administration of sage tablets on relative organ weights and thymus and spleen cellularity in mice.

## Abbreviations

The following abbreviations are used in this manuscript:

AcrE	Acarbose equivalent
AsA	Ascorbic acid equivalent
BPC	Base peak chromatogram
DBE	Number of double bonds
DMSO	Dimethyl sulfoxide
DPPH	2,2-diphenyl-1-picrylhydrazyl
EUCAST	European Committee on Antimicrobial Susceptibility Testing
FRAP	Ferric reducing antioxidant power
GAE	Gallic acid equivalent
HPLC-QTOF-ESI-MS/MS	High-performance liquid chromatography coupled to mass spectrometry
MBC	Minimum bactericidal concentration
MFC	Minimum fungicidal concentration
MH	Mueller–Hinton
MIC	Minimum inhibitory concentration
MSI	Metabolomics Standards Initiative
RT	Retention time
QE	Quercetin equivalent
SEM	Standard error of the mean
TAC	Total antioxidant capacity
TE	Trolox equivalent
TEAC	Trolox equivalent antioxidant capacity
TFC	Total flavonoid content
TPC	Total phenolic content
TPTZ	2,4,6-tris(2-pyridyl)-S-triazine
