# Supplementary material for: From Salvia dumetorum Hydroethanolic Extract to Tablet Formulation: Integrated Phytochemical, Biological, and Preclinical Safety Evaluation
Source: Molecules. 2026 Oct 22;31(21):3802. doi: 10.3390/molecules31213802 (PMC13764912; doi:10.3390/molecules31213802)
Supplement: Supplementary file 1 [file molecules-31-03802-s001.zip › Suppl.pdf]

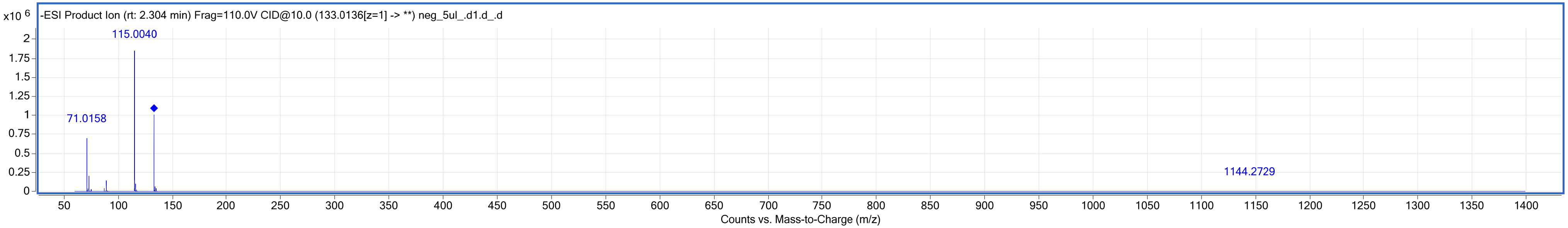

Figure S1. MS/MS fragmentation spectrum of malic acid in negative mode.

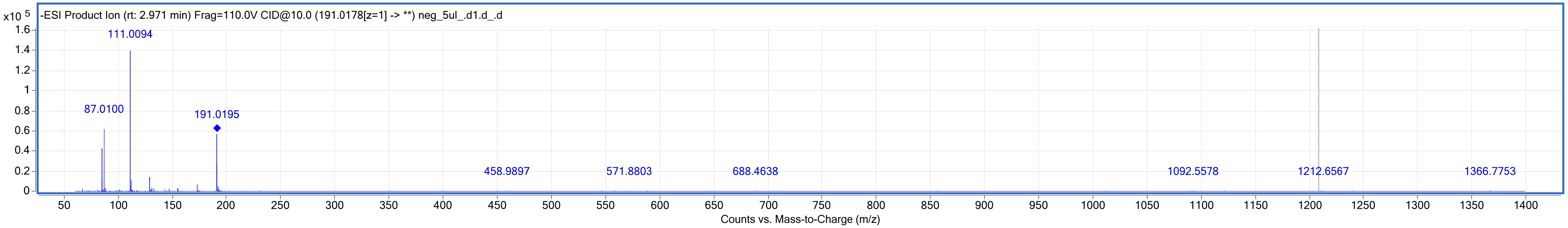

Figure S2. MS/MS fragmentation spectrum of citric acid in negative mode.

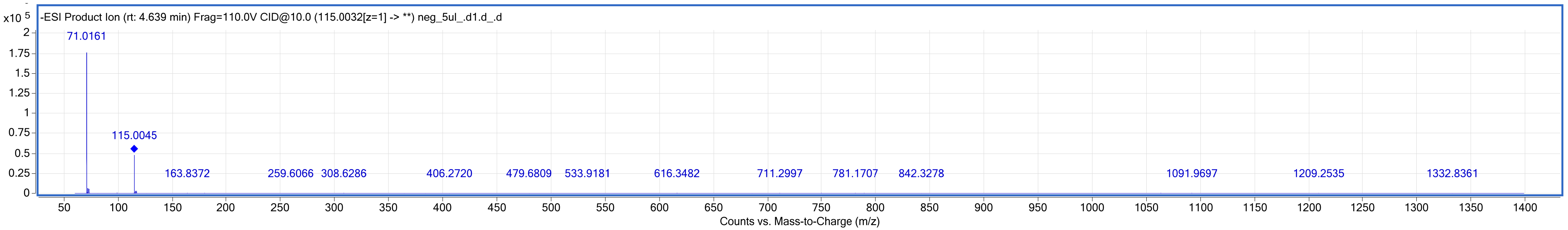

Figure S3. MS/MS fragmentation spectrum of fumaric acid in negative mode.

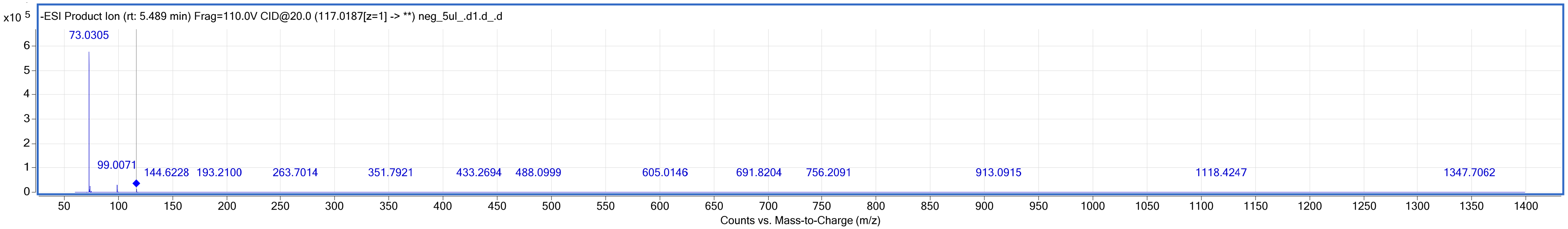

Figure S4. MS/MS fragmentation spectrum of succinic acid in negative mode.

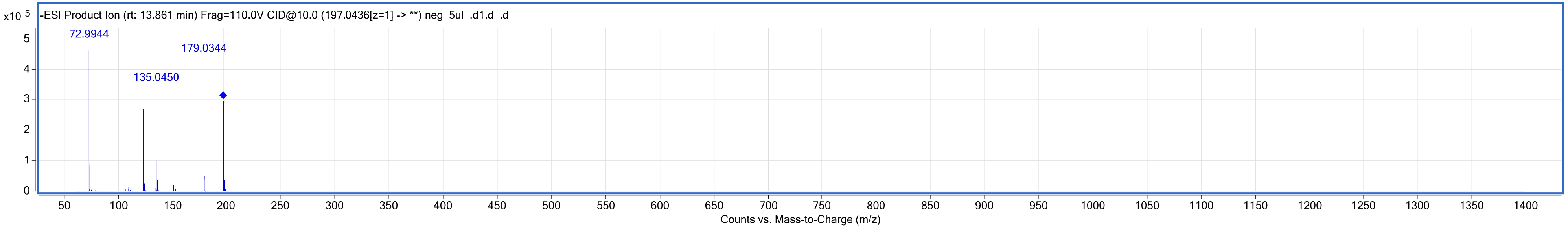

Figure S5. MS/MS fragmentation spectrum of danshensu in negative mode.

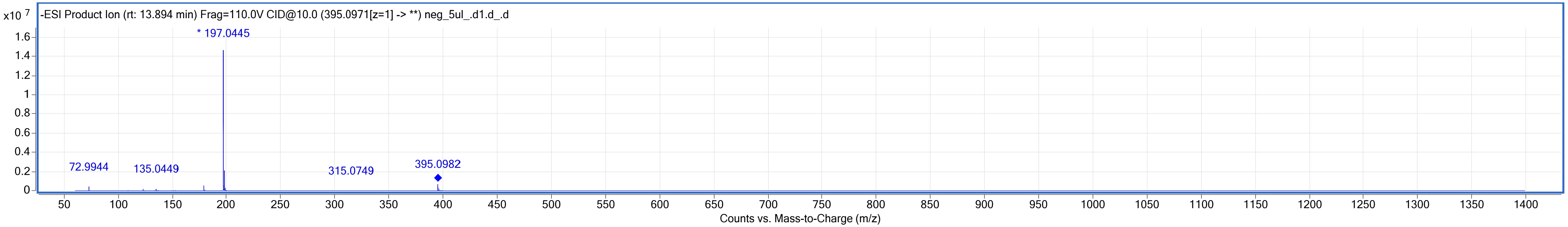

Figure S6. MS/MS fragmentation spectrum of acetylisobisflorin in negative mode.

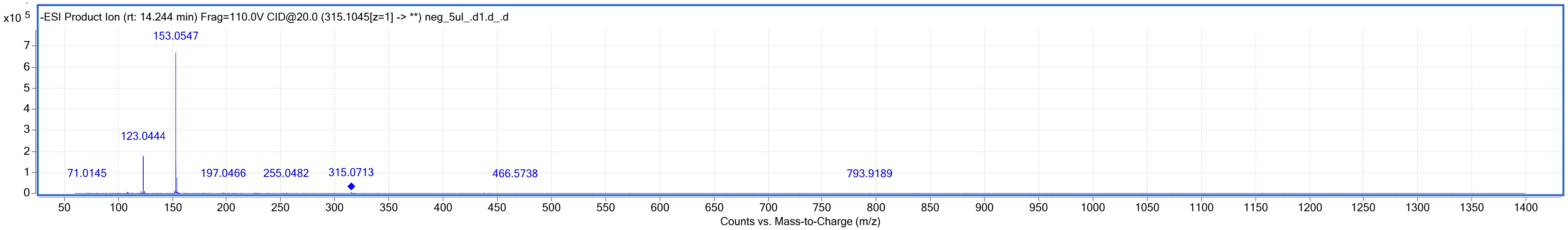

Figure S7. MS/MS fragmentation spectrum of gentioysil glucoside in negative mode.

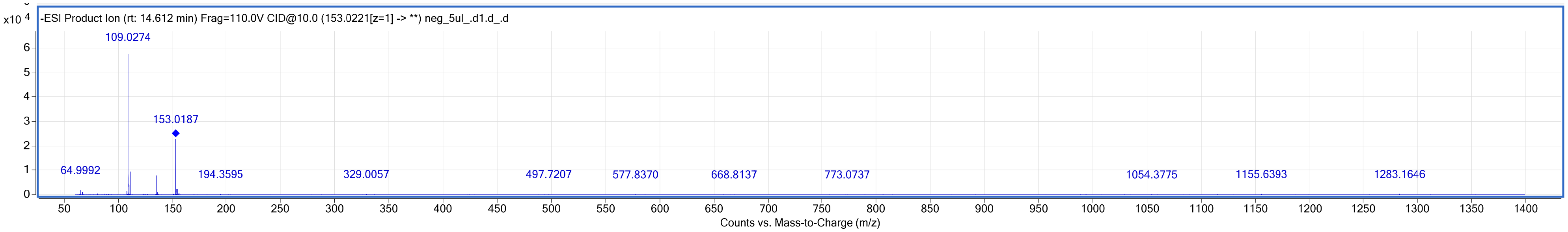

Figure S8. MS/MS fragmentation spectrum of dihydroxybenzoic acid (gentisic acid) in negative mode.

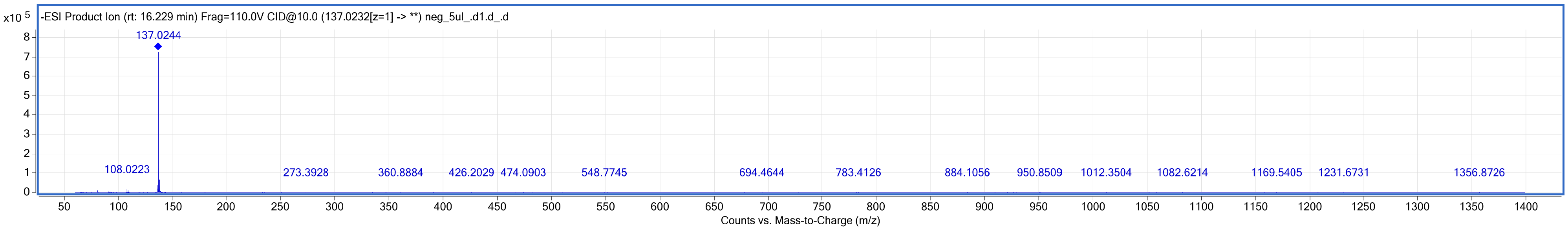

Figure S9. MS/MS fragmentation spectrum of hydroxybenzoic acid in negative mode.

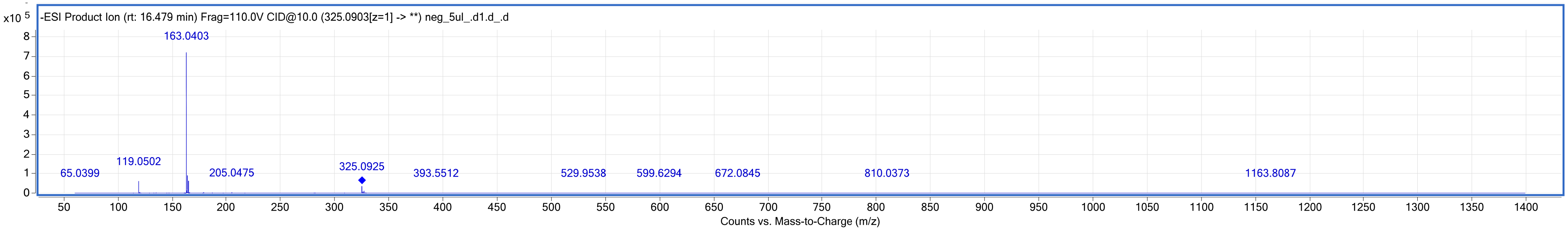

Figure S10. MS/MS fragmentation spectrum of p-Coumaric acid glucoside in negative mode.

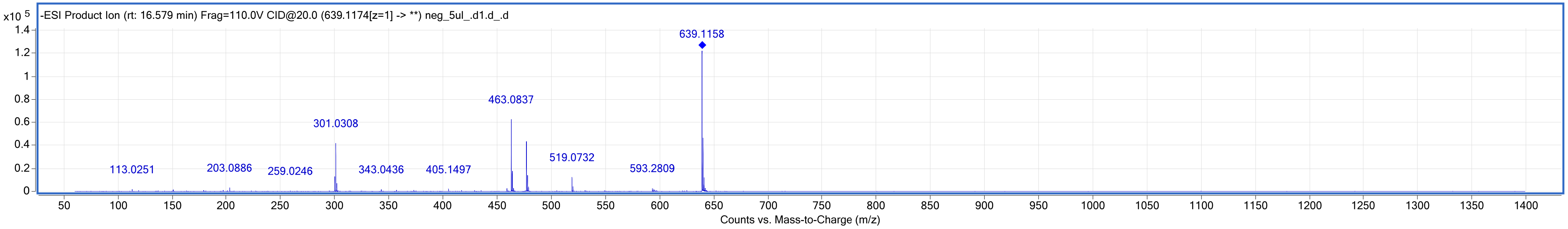

Figure S11. MS/MS fragmentation spectrum of quercetin glucosyl-glucuronide isomer 1 in negative mode.

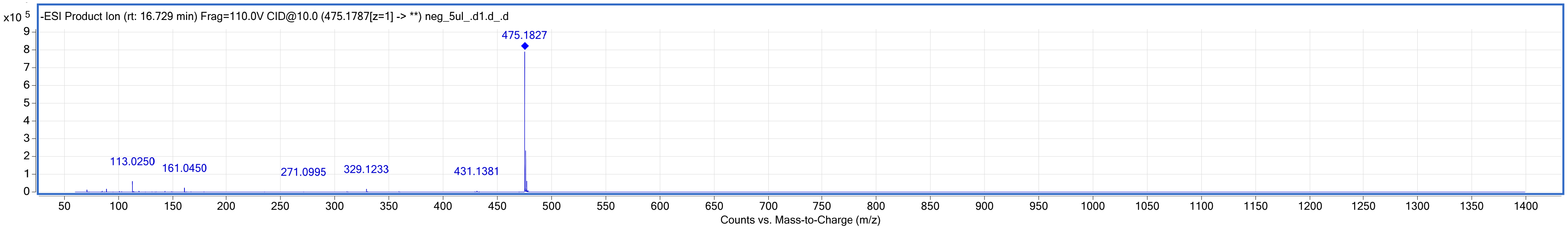

Figure S12. MS/MS fragmentation spectrum of cistanoside E in negative mode.

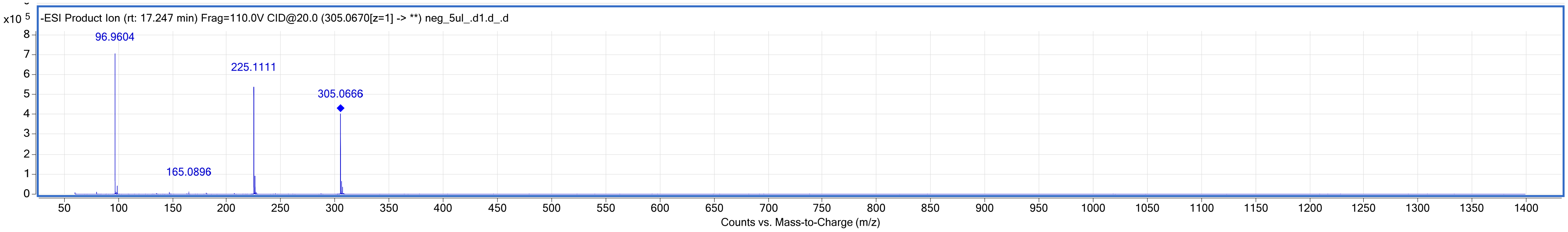

Figure S13. MS/MS fragmentation spectrum of epigallocatechin in negative mode.

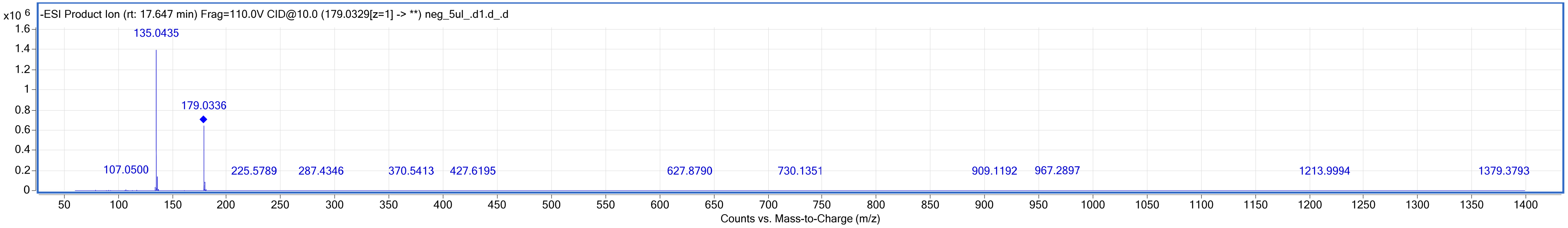

Figure S14. MS/MS fragmentation spectrum of caffeic acid in negative mode.

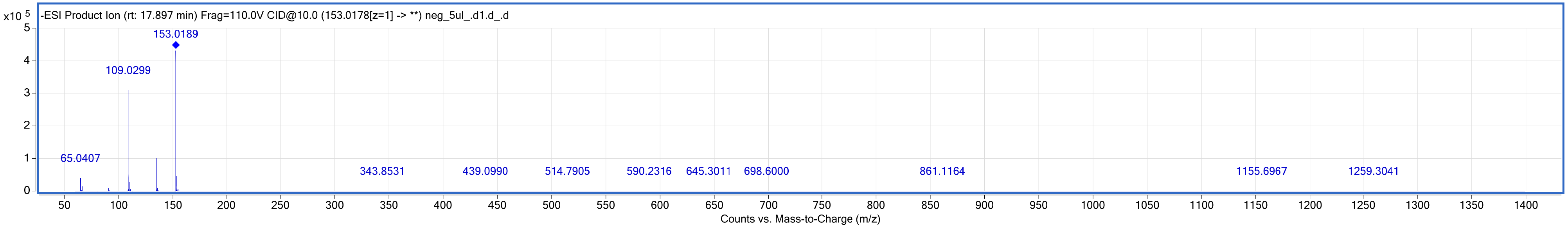

Figure S15. MS/MS fragmentation spectrum of protocatechuic acid in negative mode.

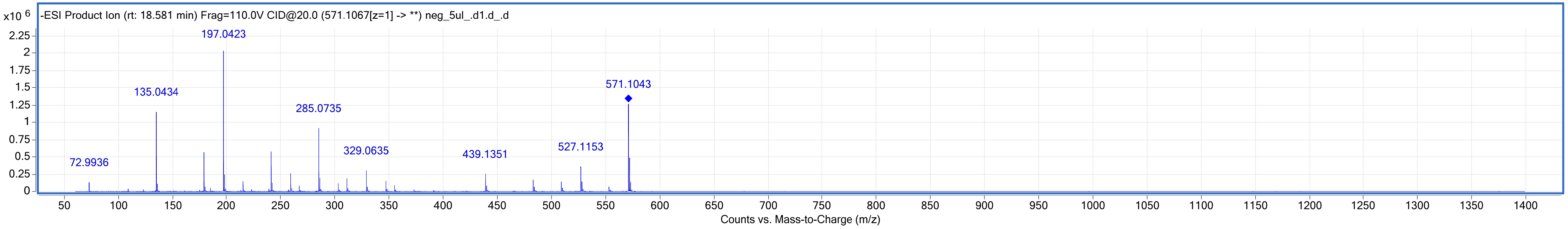

Figure S16. MS/MS fragmentation spectrum of yunnaneic acid E in negative mode.

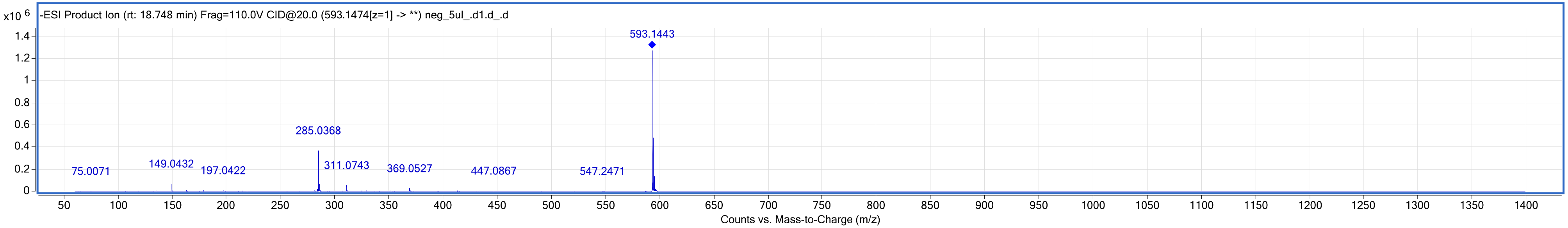

Figure S17. MS/MS fragmentation spectrum of luteolin rutinoside derivative in negative mode.

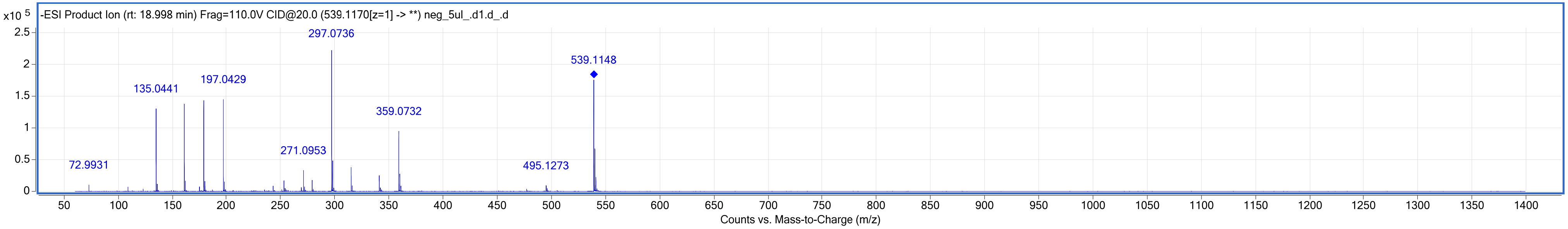

Figure S18. MS/MS fragmentation spectrum of yunnaneic acid D isomer 1 in negative mode.

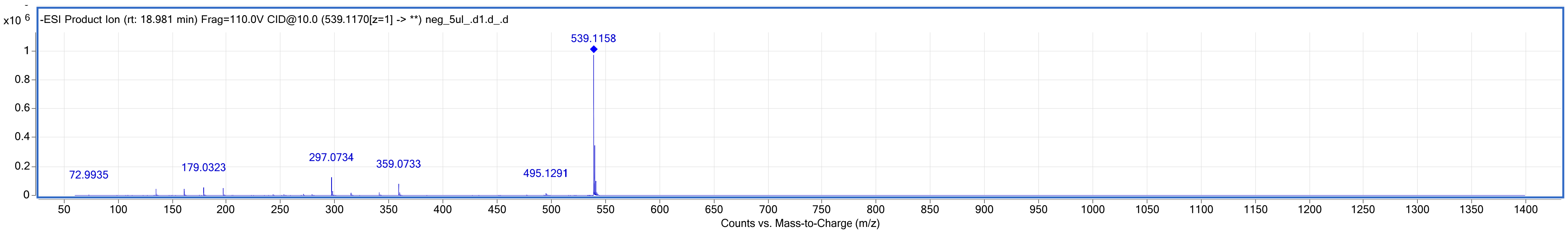

Figure S19. MS/MS fragmentation spectrum of yunnaneic acid D isomer 2 in negative mode.

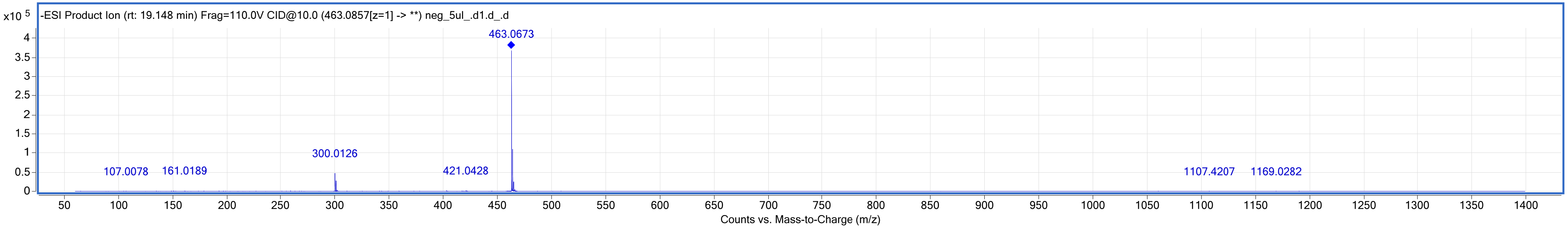

Figure S20. MS/MS fragmentation spectrum of castilicetin in negative mode.

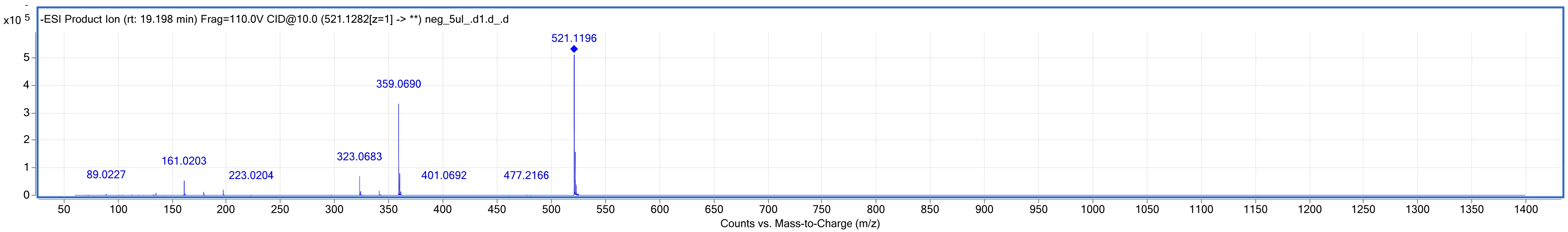

Figure S21. MS/MS fragmentation spectrum of salviaflaside in negative mode.

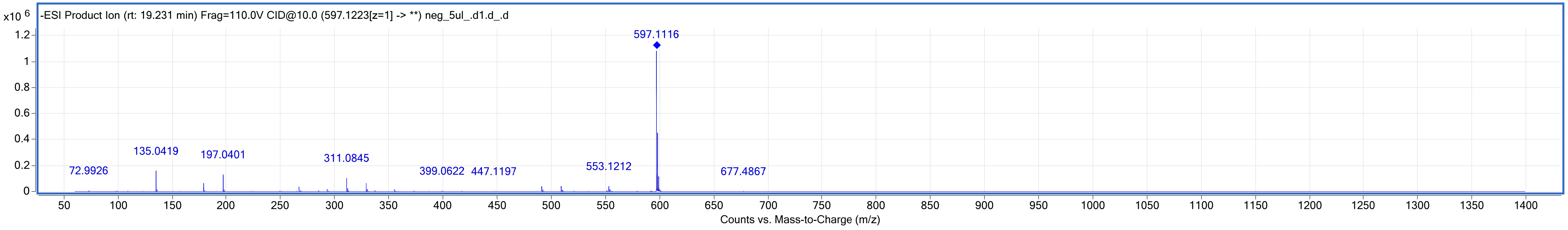

Figure S22. MS/MS fragmentation spectrum of yunnaneic acid F in negative mode.

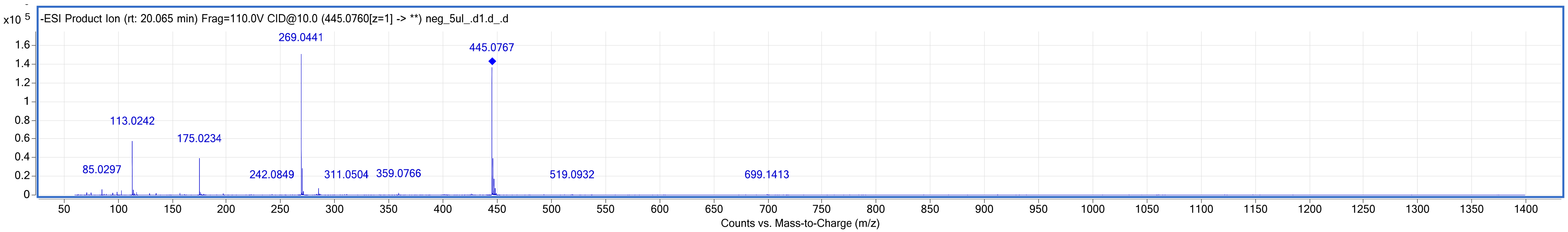

Figure S23. MS/MS fragmentation spectrum of apigenin glucuronide in negative mode.

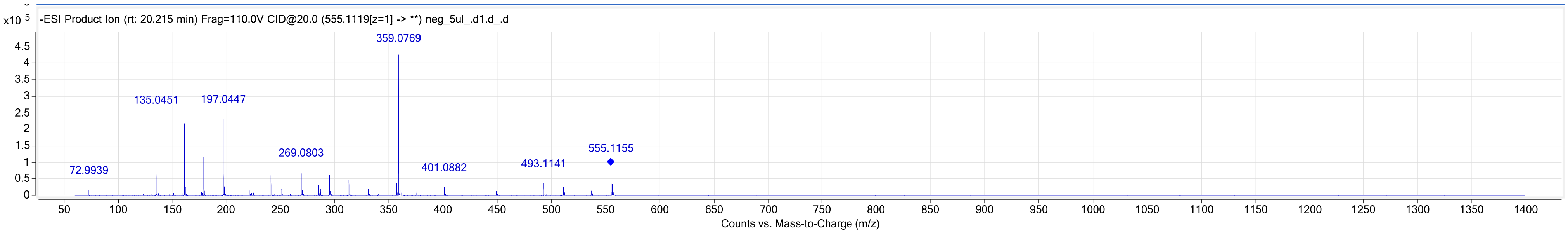

Figure S24. MS/MS fragmentation spectrum of salvianolic acid K in negative mode.

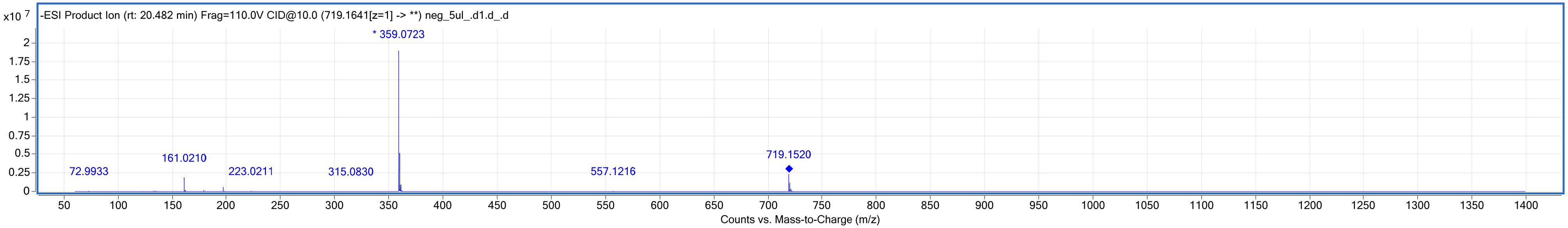

Figure S25. MS/MS fragmentation spectrum of sagerinic acid in negative mode.

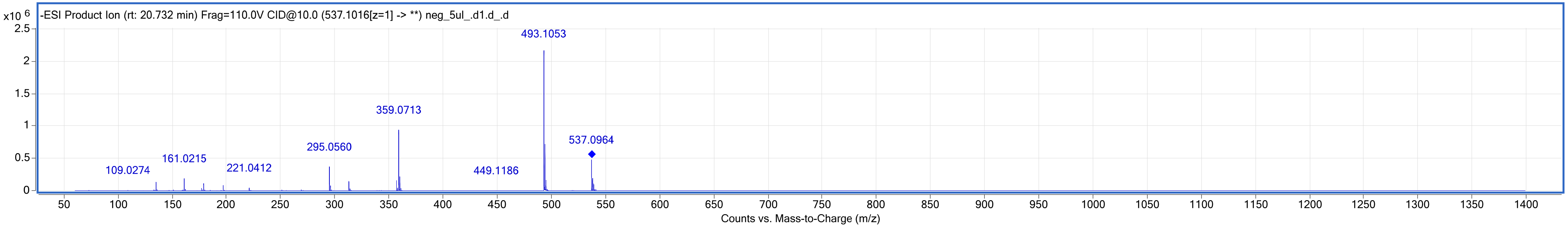

Figure S26. MS/MS fragmentation spectrum of lithospermic acid A in negative mode.

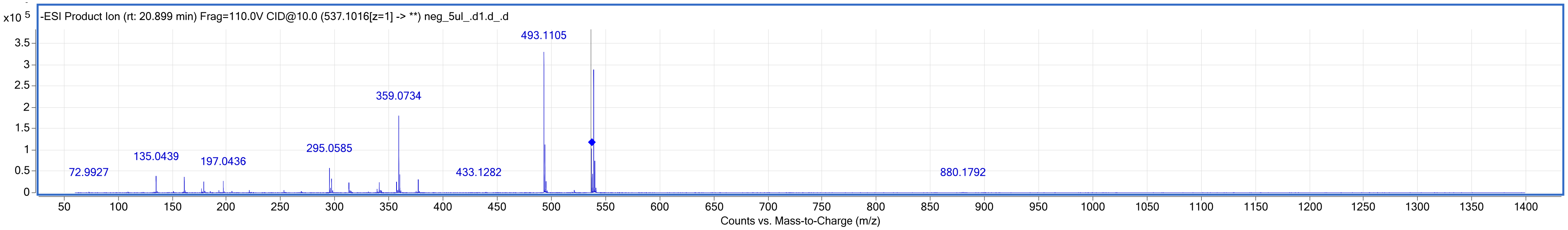

Figure S27. MS/MS fragmentation spectrum of lithospermic acid B (salvianolic acid B) in negative mode.

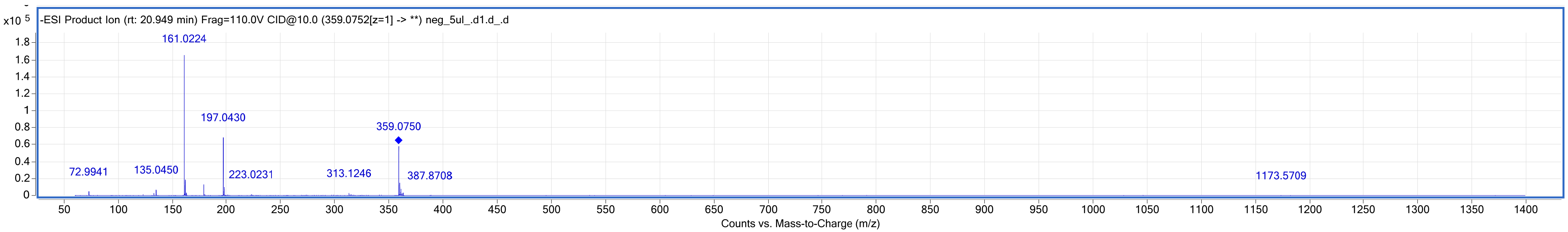

Figure S28. MS/MS fragmentation spectrum of rosmarinic acid in negative mode.

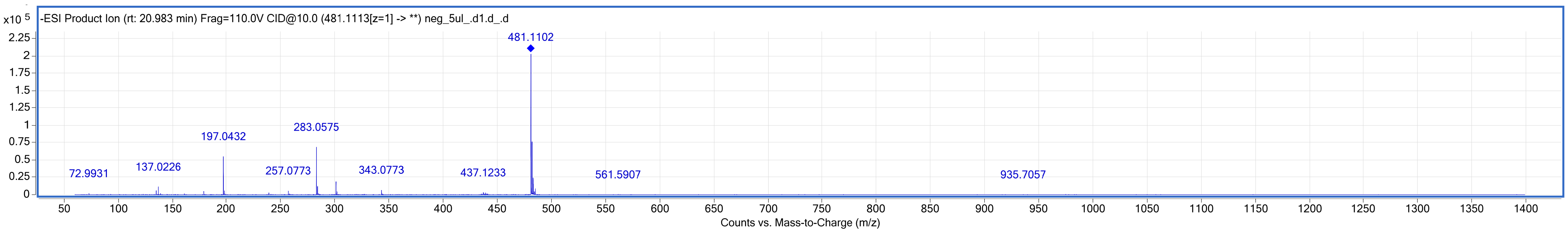

Figure S29. MS/MS fragmentation spectrum of silybin in negative mode.

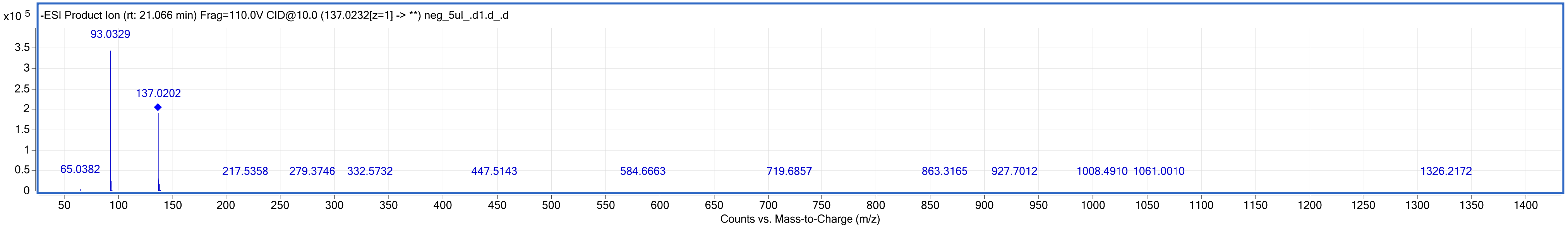

Figure S30. MS/MS fragmentation spectrum of protocatechuic aldehyde in negative mode.

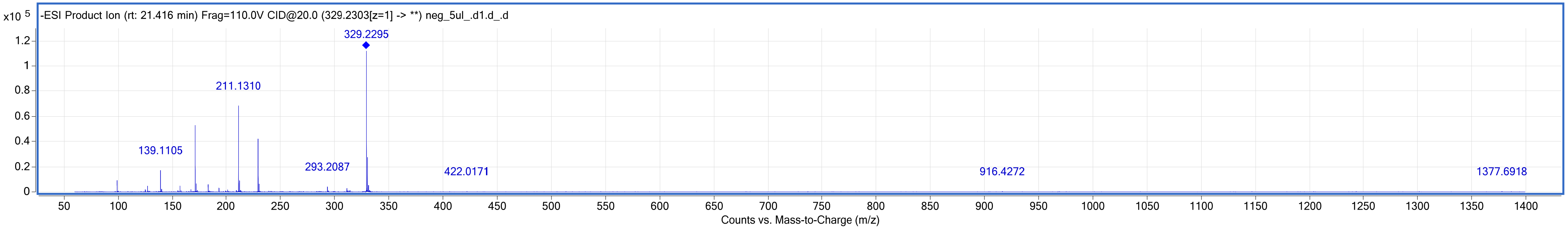

Figure S31. MS/MS fragmentation spectrum of pinellic acid in negative mode.

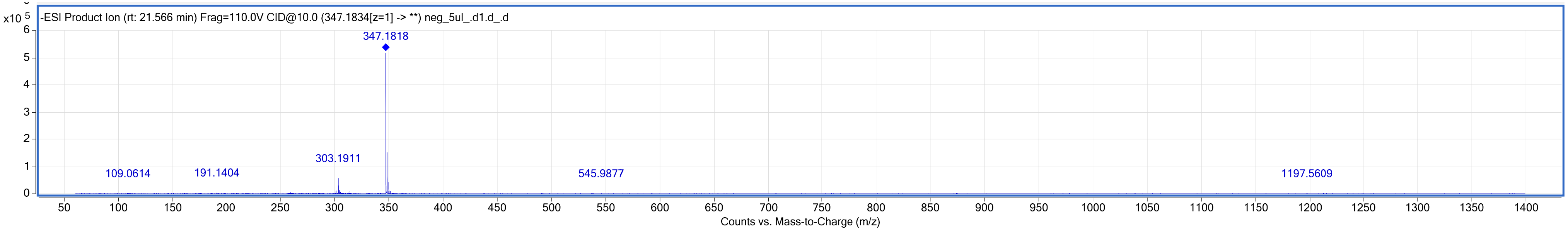

Figure S32. MS/MS fragmentation spectrum of hydroxycarnosic acid in negative mode.

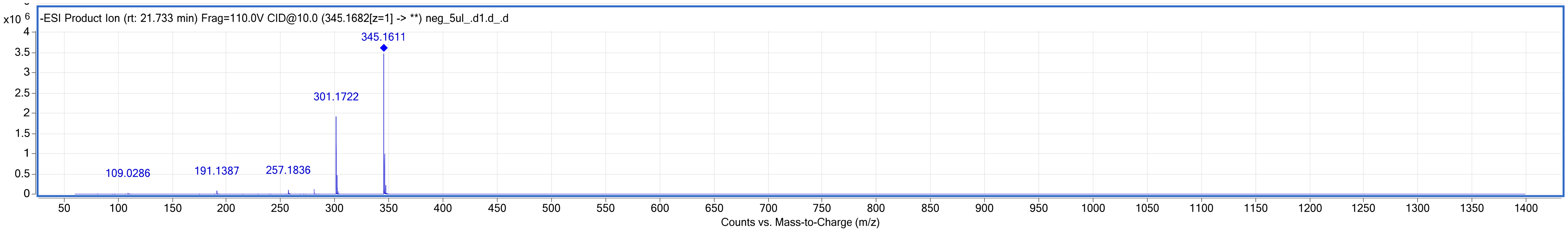

Figure S33. MS/MS fragmentation spectrum of rosmanol in negative mode.

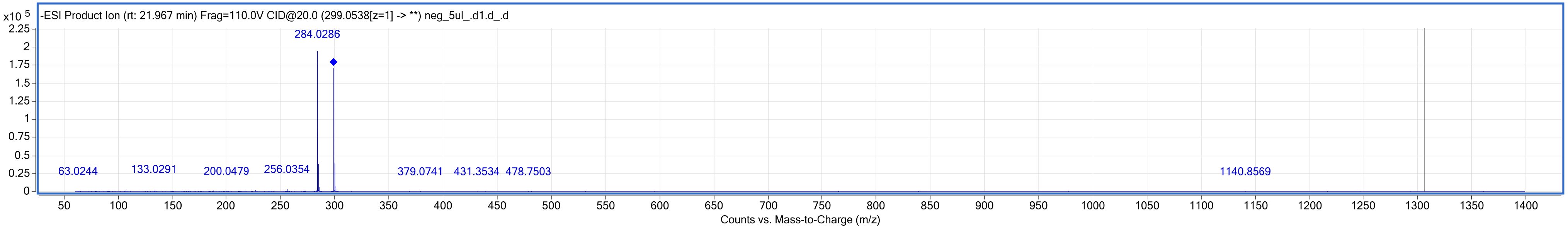

Figure S34. MS/MS fragmentation spectrum of hispidulin in negative mode.

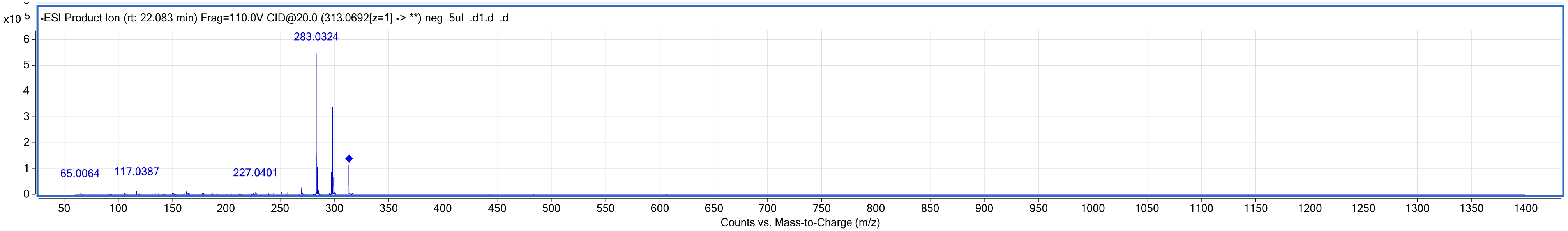

Figure S35. MS/MS fragmentation spectrum of cirsimaritin in negative mode.

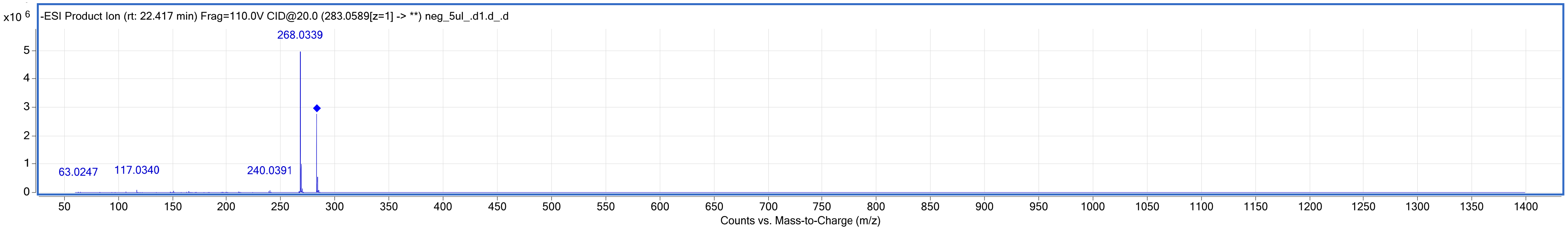

Figure S36. MS/MS fragmentation spectrum of genkwanin in negative mode.

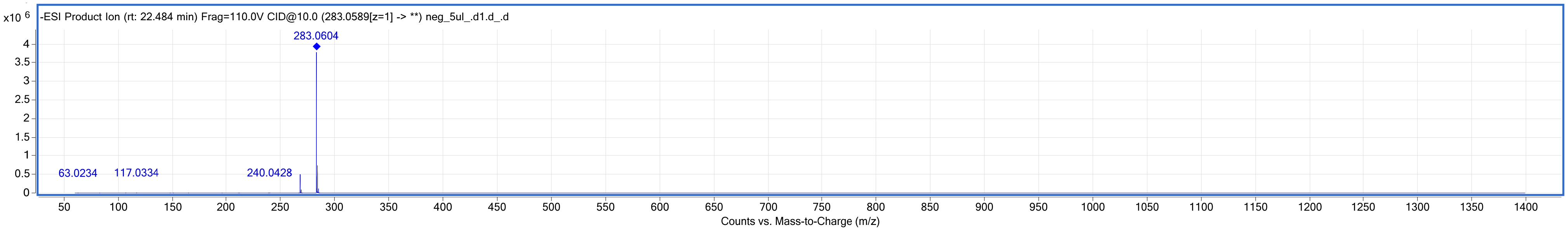

Figure S37. MS/MS fragmentation spectrum of acacetin in negative mode.

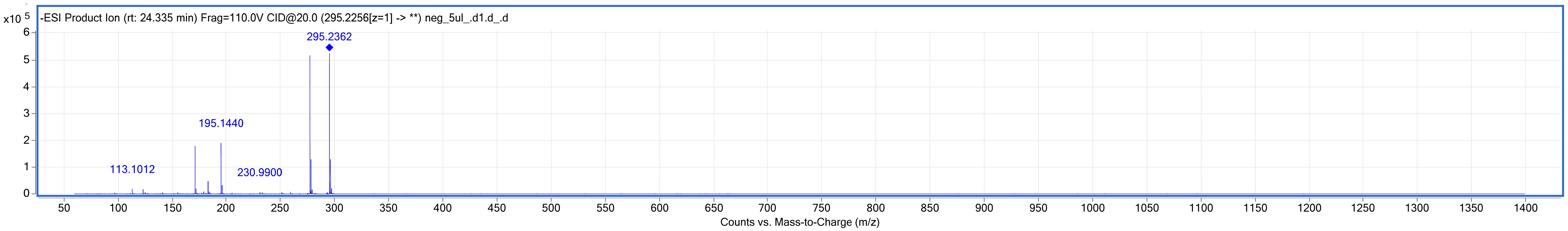

Figure S38. MS/MS fragmentation spectrum of hydroxylinoleic acid in negative mode.

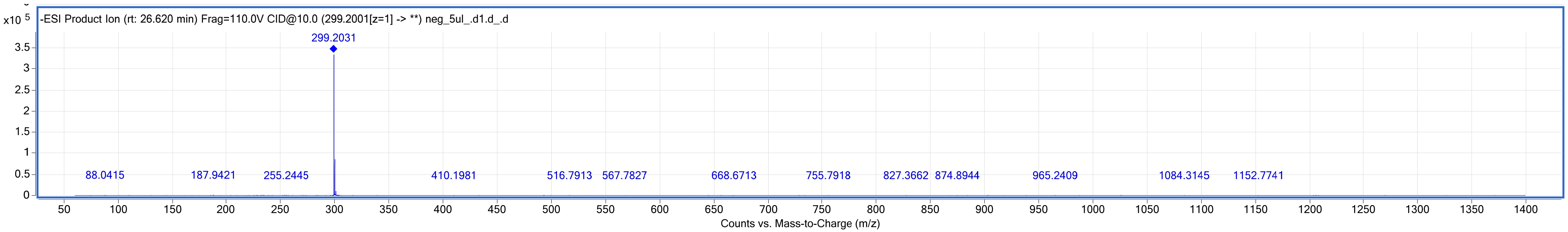

Figure S39. MS/MS fragmentation spectrum of sugiol in negative mode.

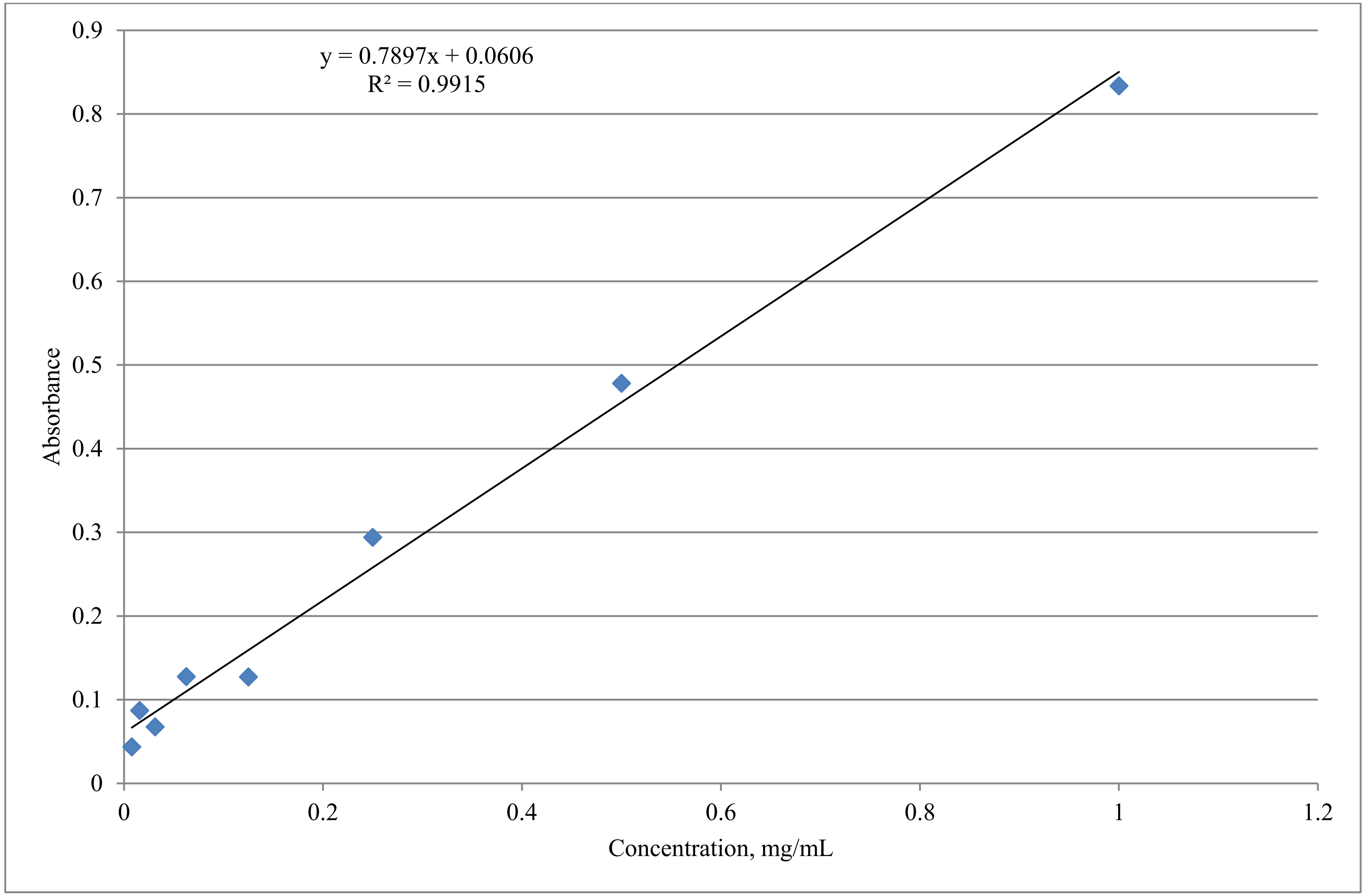

Figure S40. Gallic acid calibration curve.

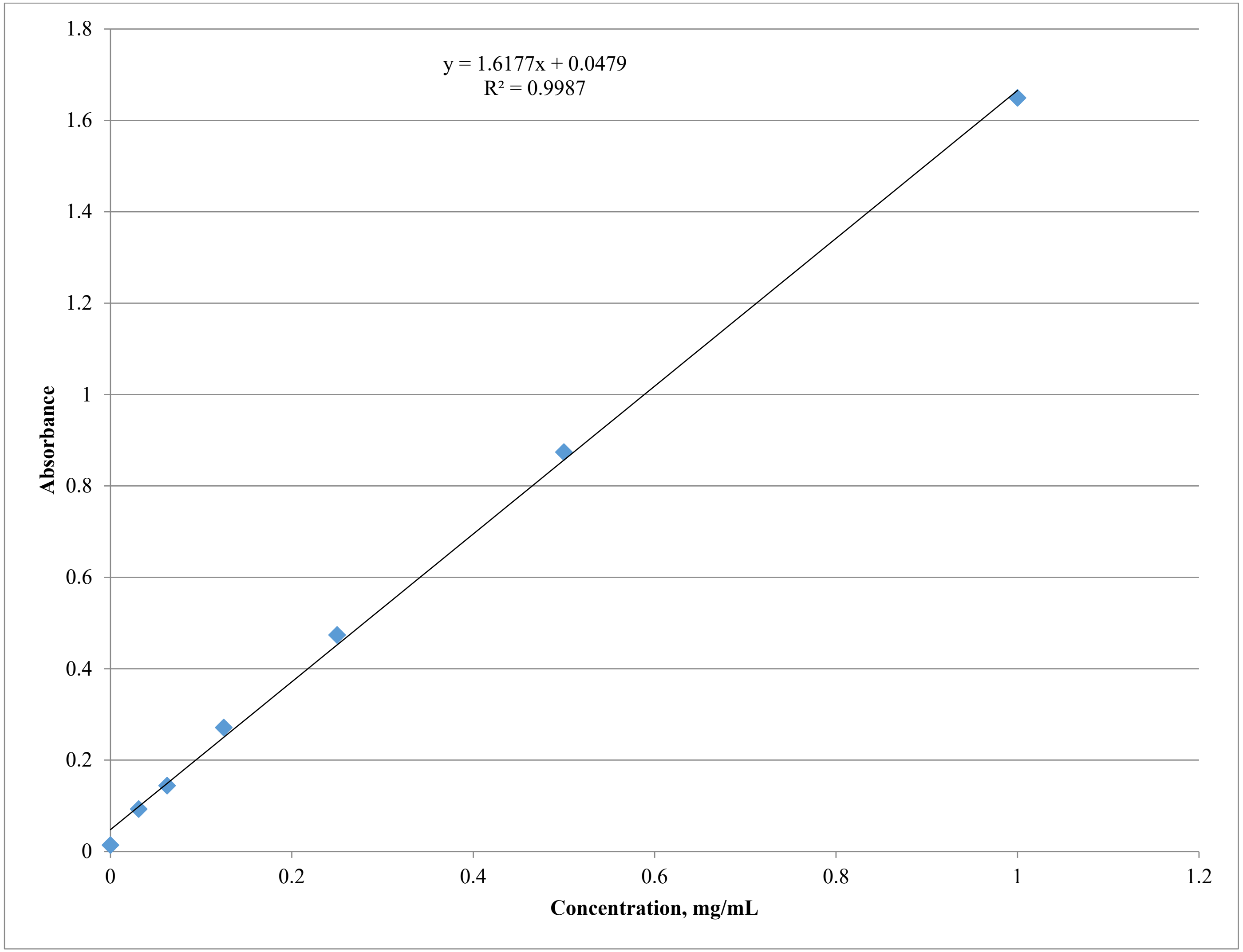

Figure S41. Quercetin calibration curve.

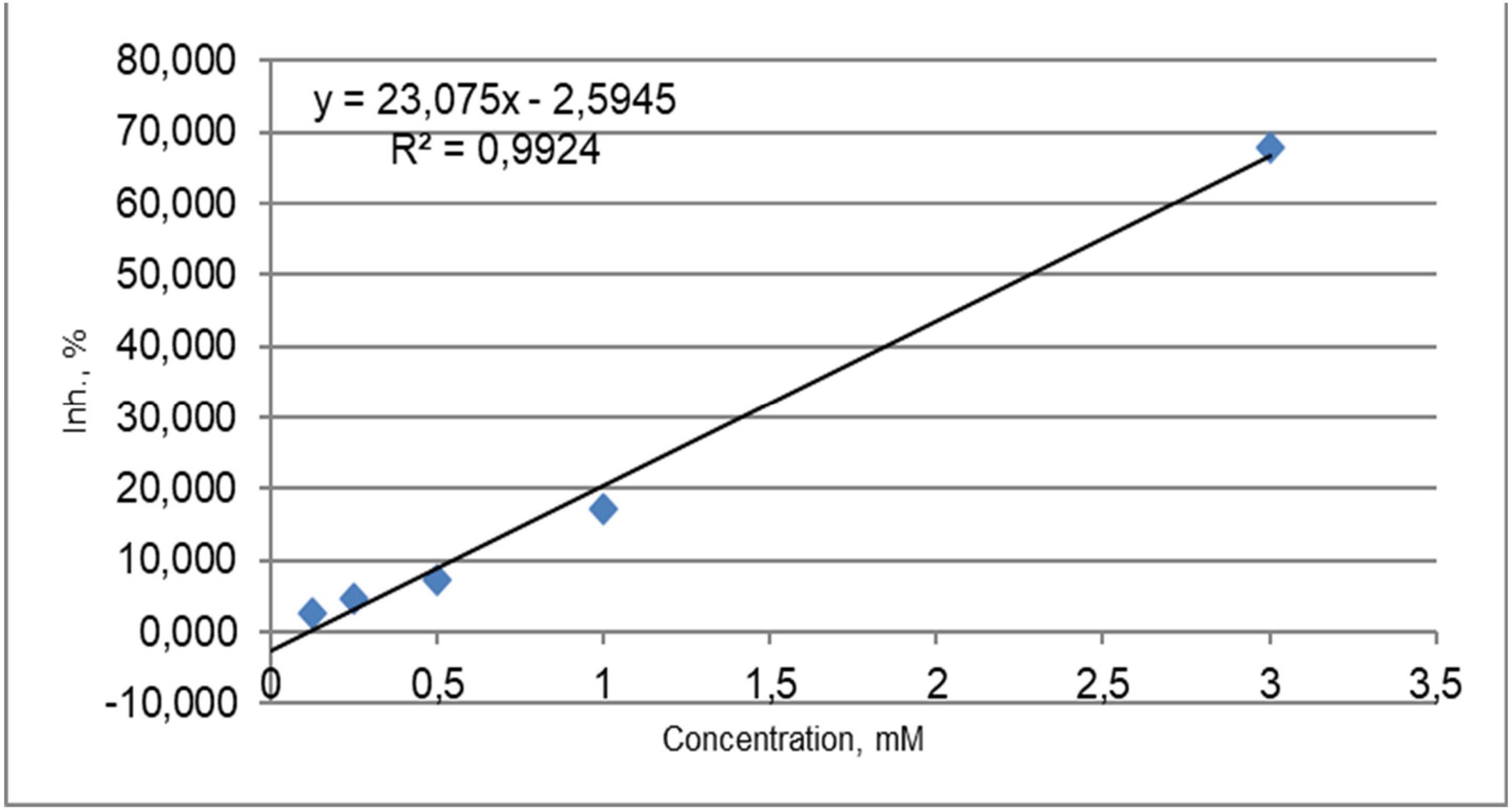

Figure S42. Calibration curve for Trolox.

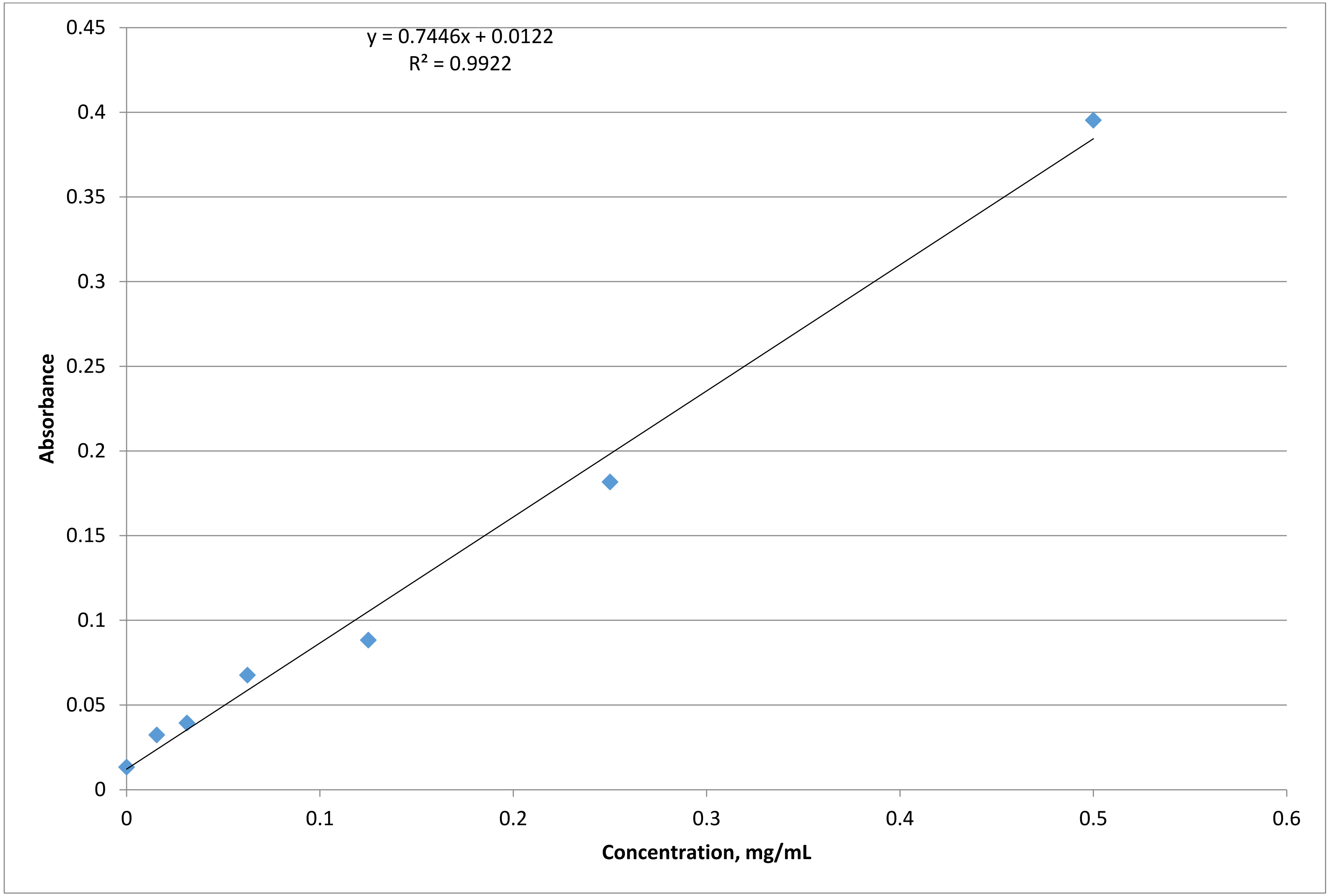

Figure S43. Calibration curve obtained with ascorbic acid.

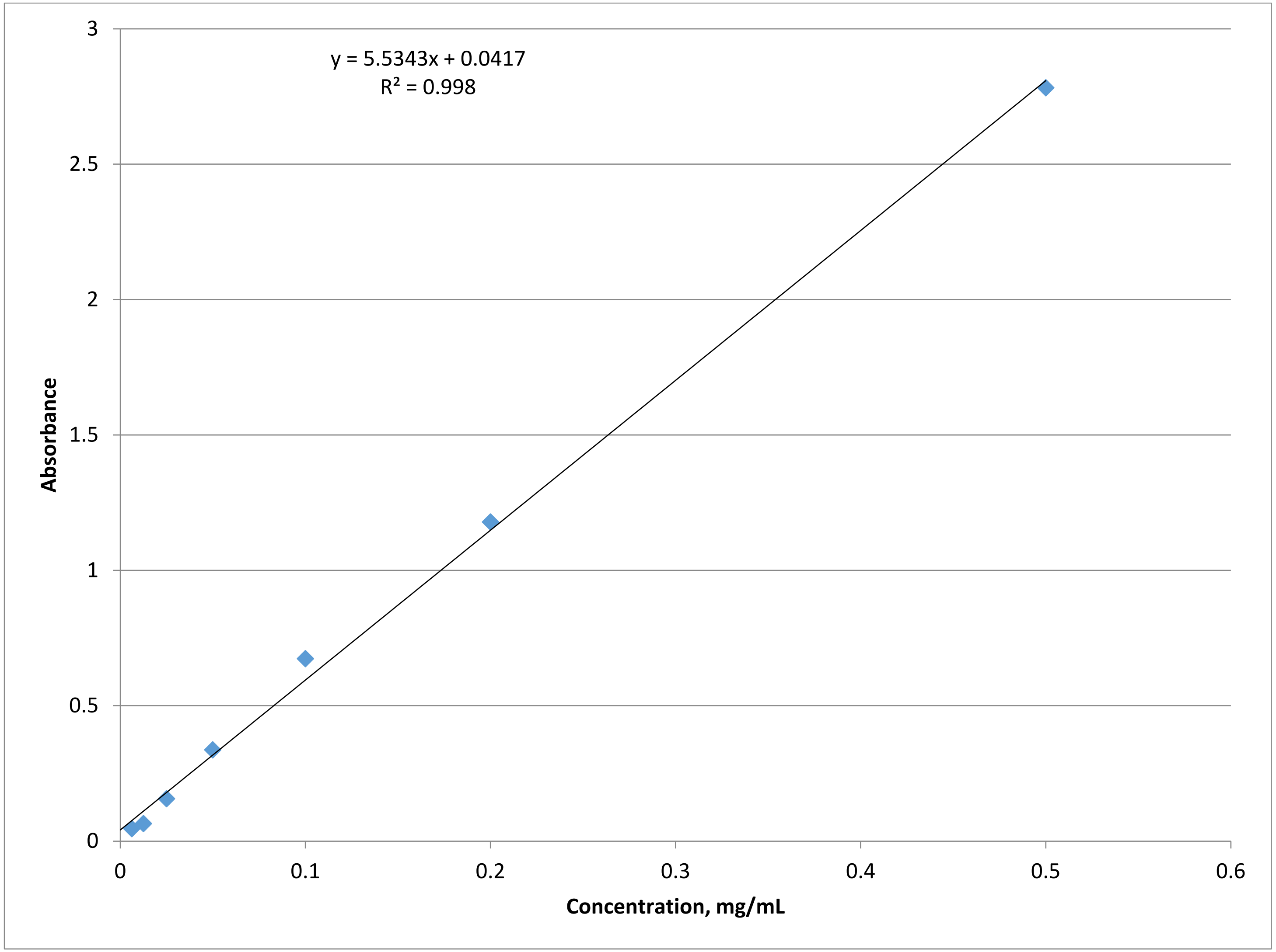

Figure S44. Calibration curve for Trolox.

Table S1. Detailed hematological parameters of mice following three weeks of repeated administration of *Salvia dumetorum* tablets and after a three-week recovery period.

Parameter	Unit	Sex	Period	Intact control	Vehicle control	100 mg/kg	250 mg/kg	1000 mg/kg
Erythrocytes	$\times 10^{12}$/L	Male	Treatment	8.1 ± 0.30	8.4 ± 0.26	7.7 ± 0.14	8.5 ± 0.22	7.9 ± 0.49
		Female	Treatment	7.7 ± 0.40	8.2 ± 0.40	8.1 ± 0.11	8.2 ± 0.11	8.5 ± 0.22
		Male	Recovery	8.4 ± 0.27	8.0 ± 0.25	8.3 ± 0.30	8.3 ± 0.27	8.4 ± 0.32
		Female	Recovery	8.0 ± 0.17	7.8 ± 0.66	7.6 ± 0.64	7.4 ± 0.30	8.1 ± 0.44
Hemoglobin	g/L	Male	Treatment	139.7 ± 0.88	137.7 ± 3.71	133.3 ± 2.86	132.0 ± 1.53	131.0 ± 6.81
		Female	Treatment	142.3 ± 3.48	139.7 ± 3.84	132.0 ± 1.53	149.0 ± 3.06	147.0 ± 7.21
		Male	Recovery	128.3 ± 3.76	131.7 ± 3.28	130.0 ± 5.29	131.0 ± 2.52	139.0 ± 4.93
		Female	Recovery	128.0 ± 4.93	115.0 ± 7.55	115.0 ± 5.00	114.7 ± 2.85	124.0 ± 2.89
Color index	—	Male	Treatment	0.50 ± 0.02	0.40 ± 0.00	0.40 ± 0.01	0.40 ± 0.01	0.50 ± 0.01
		Female	Treatment	0.50 ± 0.02	0.50 ± 0.03	0.40 ± 0.01	0.50 ± 0.00	0.50 ± 0.01
		Male	Recovery	0.40 ± 0.01	0.50 ± 0.01	0.40 ± 0.01	0.40 ± 0.01	0.50 ± 0.01
		Female	Recovery	0.40 ± 0.01	0.40 ± 0.04	0.40 ± 0.05	0.40 ± 0.02	0.40 ± 0.01
Clotting time	s	Male	Treatment	36.0 ± 3.21	33.7 ± 1.86	32.7 ± 1.76	31.7 ± 3.53	33.3 ± 1.33
		Female	Treatment	29.0 ± 3.21	24.3 ± 0.88	23.3 ± 1.67	24.3 ± 0.88	26.0 ± 5.77
		Male	Recovery	37.0 ± 2.52	36.0 ± 3.61	39.7 ± 3.93	37.7 ± 4.71	33.7 ± 4.98
		Female	Recovery	35.7 ± 3.18	37.3 ± 2.67	36.0 ± 0.58	29.7 ± 3.18	33.0 ± 1.15
ESR	mm/h	Male	Treatment	2.3 ± 0.33	1.7 ± 0.33	2.3 ± 0.88	2.7 ± 0.67	1.3 ± 0.33
		Female	Treatment	1.0 ± 0.00	1.3 ± 0.33	1.3 ± 0.33	1.0 ± 0.00	1.0 ± 0.00
		Male	Recovery	1.7 ± 0.33	1.3 ± 0.33	1.3 ± 0.33	1.7 ± 0.67	1.7 ± 0.33
		Female	Recovery	1.3 ± 0.33	1.7 ± 0.33	2.0 ± 0.00	1.7 ± 0.33	1.3 ± 0.33
Hematocrit	L/L	Male	Treatment	42.7 ± 0.69	43.3 ± 0.93	42.9 ± 0.98	42.9 ± 0.52	41.9 ± 1.29
		Female	Treatment	43.0 ± 1.72	42.8 ± 0.90	43.4 ± 0.62	45.1 ± 1.36	46.4 ± 4.16
		Male	Recovery	33.2 ± 1.11	35.7 ± 1.35	35.2 ± 1.91	36.2 ± 1.94	36.2 ± 1.28
		Female	Recovery	38.1 ± 1.15	32.2 ± 2.76	35.3 ± 1.06	34.0 ± 1.00	35.4 ± 1.83

Platelets	×10⁹/L	Male	Treatment	598.3 ± 37.20	605.7 ± 46.40	637.0 ± 21.55	522.7 ± 61.50	608.3 ± 10.73
		Female	Treatment	575.3 ± 42.98	568.0 ± 70.53	547.7 ± 50.11	477.0 ± 14.57	582.7 ± 38.68
		Male	Recovery	607.0 ± 49.43	611.3 ± 34.83	651.0 ± 20.79	534.0 ± 53.59	575.0 ± 15.52
		Female	Recovery	533.7 ± 7.36	573.3 ± 17.19	565.3 ± 27.72	542.3 ± 16.75	565.3 ± 27.72
Leukocytes	×10⁹/L	Male	Treatment	11.3 ± 1.49	11.1 ± 0.77	12.1 ± 1.21	12.5 ± 1.91	11.7 ± 0.95
		Female	Treatment	8.4 ± 0.38	8.0 ± 0.75	9.7 ± 1.35	14.5 ± 1.56	9.0 ± 0.49
		Male	Recovery	14.8 ± 1.13	14.6 ± 1.85	15.3 ± 2.36	15.6 ± 1.89	13.3 ± 1.13
		Female	Recovery	7.3 ± 1.56	7.6 ± 1.21	8.8 ± 1.23	8.8 ± 0.75	8.7 ± 0.78
Band neutrophils	%	Male	Treatment	3.3 ± 1.45	3.7 ± 0.88	2.3 ± 0.88	3.3 ± 0.33	3.0 ± 1.53
		Female	Treatment	4.7 ± 1.45	3.0 ± 1.00	4.3 ± 0.33	1.7 ± 0.67	3.3 ± 2.33
		Male	Recovery	1.3 ± 0.33	1.7 ± 0.33	1.4 ± 0.33	1.3 ± 1.45	1.7 ± 0.67
		Female	Recovery	5.0 ± 1.00	3.3 ± 0.88	3.3 ± 0.67	4.0 ± 1.53	3.3 ± 1.33
Segmented neutrophils	%	Male	Treatment	12.7 ± 2.19	10.7 ± 3.84	9.7 ± 1.67	20.0 ± 3.21	15.3 ± 3.76
		Female	Treatment	21.3 ± 6.49	20.3 ± 6.17	13.3 ± 3.84	12.3 ± 5.24	15.7 ± 1.20
		Male	Recovery	11.7 ± 2.03	19.3 ± 5.90	13.7 ± 5.93	18.3 ± 5.21	15.0 ± 4.04
		Female	Recovery	23.0 ± 5.51	21.0 ± 4.58	11.0 ± 3.00	23.3 ± 4.67	11.0 ± 1.53
Eosinophils	%	Male	Treatment	2.7 ± 0.33	2.7 ± 0.67	2.7 ± 0.33	2.0 ± 0.58	4.3 ± 1.67
		Female	Treatment	2.0 ± 0.58	2.7 ± 1.20	0.7 ± 0.33	0.7 ± 0.33	2.7 ± 0.88
		Male	Recovery	0.7 ± 0.33	2.3 ± 0.88	3.0 ± 0.58	1.3 ± 0.88	1.3 ± 0.33
		Female	Recovery	3.3 ± 1.20	2.7 ± 1.20	2.7 ± 0.67	2.0 ± 0.58	1.3 ± 0.33
Basophils	%	Male	Treatment	0.0 ± 0.00	0.0 ± 0.00	0.0 ± 0.00	0.0 ± 0.00	0.3 ± 0.33
		Female	Treatment	0.0 ± 0.00	0.0 ± 0.00	0.0 ± 0.00	0.0 ± 0.00	0.0 ± 0.00
		Male	Recovery	0.0 ± 0.00	0.3 ± 0.33	0.3 ± 0.33	0.0 ± 0.00	0.0 ± 0.00
		Female	Recovery	0.0 ± 0.00	0.0 ± 0.00	0.0 ± 0.00	0.0 ± 0.00	0.0 ± 0.00
Monocytes	%	Male	Treatment	1.3 ± 0.33	1.7 ± 0.33	2.3 ± 0.33	1.7 ± 0.33	1.3 ± 0.33
		Female	Treatment	2.3 ± 1.33	3.0 ± 1.15	1.7 ± 0.88	2.0 ± 0.58	1.3 ± 0.33
		Male	Recovery	1.3 ± 0.33	1.7 ± 0.33	1.0 ± 0.00	1.0 ± 0.58	1.0 ± 0.00

		Female	Recovery	3.3 ± 0.88	4.0 ± 1.15	2.3 ± 0.33	3.0 ± 0.58	2.0 ± 0.00
Lymphocytes	%	Male	Treatment	80.0 ± 1.15	82.0 ± 3.79	83.0 ± 1.73	73.0 ± 2.52	76.7 ± 5.33
		Female	Treatment	69.7 ± 6.12	71.0 ± 8.19	80.0 ± 4.58	84.3 ± 4.91	77.0 ± 3.00
		Male	Recovery	85.0 ± 2.89	73.7 ± 5.24	80.7 ± 5.81	77.0 ± 7.00	81.0 ± 4.93
		Female	Recovery	65.3 ± 4.67	69.0 ± 3.46	70.7 ± 3.93	67.7 ± 5.36	72.3 ± 2.67
Phagocytic index	%	Male	Treatment	29.17 ± 3.91	27.46 ± 4.72	29.84 ± 4.52	30.01 ± 2.74	28.39 ± 3.76
		Female	Treatment	28.35 ± 2.78	30.08 ± 4.21	29.34 ± 3.56	30.31 ± 2.89	28.35 ± 2.87
		Male	Recovery	30.06 ± 4.27	28.28 ± 3.86	29.24 ± 3.46	27.26 ± 3.74	28.97 ± 4.84
		Female	Recovery	28.29 ± 3.74	29.25 ± 2.86	30.02 ± 4.05	27.97 ± 4.83	29.23 ± 2.96

Values are presented as mean ± SEM. Treatment indicates the end of the three-week administration period, whereas recovery indicates three weeks after treatment discontinuation. No statistically significant differences were observed between treated and control groups ($p > 0.05$).

Table S2. Detailed plasma biochemical and immunological parameters in mice following repeated administration of *Salvia dumetorum* tablets and after the recovery period.

Parameter	Unit	Sex	Period	Intact control	Vehicle control	100 mg/kg	250 mg/kg	1000 mg/kg
**Glucose**	mmol/L	Male	Treatment	6.16 ± 0.68	6.25 ± 0.81	7.51 ± 0.59	5.75 ± 0.53	5.35 ± 0.28
		Female	Treatment	6.08 ± 0.63	6.55 ± 0.73	6.66 ± 0.65	7.09 ± 0.49	5.59 ± 0.45
		Male	Recovery	5.05 ± 0.47	5.65 ± 0.44	5.03 ± 0.33	5.11 ± 0.36	4.66 ± 0.27
		Female	Recovery	4.73 ± 0.44	5.28 ± 0.15	4.79 ± 0.35	4.97 ± 0.31	4.94 ± 0.26
**Total protein**	g/L	Male	Treatment	82.75 ± 11.00	78.72 ± 10.52	81.32 ± 10.85	83.17 ± 7.29	81.45 ± 2.81
		Female	Treatment	108.64 ± 3.62	96.62 ± 4.33	100.83 ± 2.98	94.38 ± 10.58	102.79 ± 10.91
		Male	Recovery	72.25 ± 1.42	67.99 ± 1.71	71.03 ± 2.81	67.57 ± 0.53	74.92 ± 2.35

		Female	Recovery	71.34 ± 1.47	75.59 ± 4.44	71.58 ± 1.13	72.19 ± 2.96	70.61 ± 2.69
**Creatinine**	µmol/L	Male	Treatment	207.11 ± 30.55	205.27 ± 18.22	202.81 ± 8.06	179.46 ± 2.66	186.83 ± 9.14
		Female	Treatment	165.32 ± 10.54	189.91 ± 11.98	200.97 ± 15.10	199.74 ± 5.71	171.47 ± 9.33
		Male	Recovery	156.98 ± 3.99	158.04 ± 6.72	161.72 ± 7.06	171.21 ± 7.17	155.40 ± 3.37
		Female	Recovery	173.31 ± 1.80	173.31 ± 5.40	170.15 ± 3.03	174.89 ± 7.25	172.26 ± 3.98
**Alkaline phosphatase**	µkat/L	Male	Treatment	3.19 ± 0.39	2.91 ± 0.50	3.55 ± 0.24	3.18 ± 0.28	2.33 ± 0.08
		Female	Treatment	4.34 ± 0.88	5.01 ± 0.41	5.62 ± 1.26	3.97 ± 0.58	6.75 ± 0.97
		Male	Recovery	2.87 ± 0.62	3.54 ± 0.40	2.72 ± 0.42	2.83 ± 0.25	2.31 ± 0.37
		Female	Recovery	3.44 ± 0.16	3.78 ± 0.65	3.10 ± 0.28	3.39 ± 0.64	3.54 ± 0.35
**Urea**	mmol/L	Male	Treatment	9.06 ± 0.46	10.31 ± 0.67	9.44 ± 0.84	11.93 ± 1.38	13.06 ± 1.58
		Female	Treatment	9.77 ± 0.69	11.32 ± 1.28	12.38 ± 1.62	10.35 ± 0.99	10.97 ± 1.23
		Male	Recovery	6.40 ± 0.21	6.27 ± 0.31	5.43 ± 0.12	5.29 ± 0.36	6.12 ± 0.48
		Female	Recovery	6.18 ± 0.20	6.57 ± 0.39	7.09 ± 0.73	6.16 ± 0.64	5.04 ± 0.68
**Alanine aminotransferase (ALT)**	µkat/L	Male	Treatment	0.41 ± 0.03	0.50 ± 0.05	0.48 ± 0.02	0.51 ± 0.07	0.42 ± 0.04
		Female	Treatment	0.57 ± 0.02	0.54 ± 0.05	0.54 ± 0.05	0.57 ± 0.08	0.49 ± 0.04
		Male	Recovery	0.34 ± 0.02	0.36 ± 0.02	0.31 ± 0.03	0.26 ± 0.04	0.33 ± 0.03
		Female	Recovery	0.35 ± 0.01	0.41 ± 0.03	0.37 ± 0.03	0.35 ± 0.05	0.39 ± 0.03

**Aspartate aminotransferase (AST)**	µkat/L	Male	Treatment	0.67 ± 0.03	0.74 ± 0.04	0.70 ± 0.05	0.69 ± 0.05	0.74 ± 0.05
		Female	Treatment	0.90 ± 0.06	0.84 ± 0.08	0.81 ± 0.08	0.76 ± 0.05	0.72 ± 0.05
		Male	Recovery	0.69 ± 0.01	0.73 ± 0.02	0.73 ± 0.04	0.70 ± 0.03	0.67 ± 0.03
		Female	Recovery	0.77 ± 0.04	0.76 ± 0.08	0.74 ± 0.02	0.77 ± 0.05	0.72 ± 0.03
**Total cholesterol**	mmol/L	Male	Treatment	1.95 ± 0.27	2.63 ± 0.41	2.64 ± 0.27	2.44 ± 0.29	2.86 ± 0.24
		Female	Treatment	2.21 ± 0.30	2.58 ± 0.40	2.52 ± 0.36	1.77 ± 0.27	2.04 ± 0.08
		Male	Recovery	1.96 ± 0.16	1.86 ± 0.13	2.06 ± 0.07	2.04 ± 0.15	2.06 ± 0.08
		Female	Recovery	1.98 ± 0.05	1.91 ± 0.11	2.25 ± 0.11	2.19 ± 0.24	2.20 ± 0.12
**Total antibody titer**	$\log_2$	Male	Treatment	5.71 ± 0.81	5.86 ± 1.12	5.74 ± 0.91	5.72 ± 1.02	5.69 ± 1.13
		Female	Treatment	4.76 ± 0.85	4.96 ± 1.13	5.04 ± 1.79	5.16 ± 1.13	5.17 ± 1.15
		Male	Recovery	5.78 ± 0.74	5.47 ± 1.19	5.47 ± 1.34	5.43 ± 1.13	5.47 ± 1.09
		Female	Recovery	4.83 ± 0.84	4.87 ± 1.15	5.12 ± 1.63	5.32 ± 1.09	5.24 ± 1.18
**IgG**	$\log_2$	Male	Treatment	5.01 ± 1.12	4.89 ± 1.07	5.09 ± 1.07	4.96 ± 1.05	4.98 ± 1.01
		Female	Treatment	5.13 ± 1.14	4.74 ± 1.08	4.78 ± 1.04	4.97 ± 1.06	4.94 ± 1.05
		Male	Recovery	5.06 ± 1.09	5.08 ± 1.13	5.04 ± 1.04	4.79 ± 1.19	4.73 ± 1.24
		Female	Recovery	5.07 ± 1.18	4.73 ± 1.16	4.65 ± 1.03	4.96 ± 1.12	4.83 ± 1.09

Values are presented as mean ± SEM. Treatment indicates the end of the three-week administration period, whereas recovery indicates three weeks after treatment discontinuation. No statistically significant differences were observed between treated and control groups ($p > 0.05$). ALT, alanine aminotransferase; AST, aspartate aminotransferase.

Table S3. Effects of 3-week oral administration of sage tablets on relative organ weights and thymus and spleen cellularity in mice

Sex	Organ/parameter	Control	Vehicle control	100 mg/kg	1000 mg/kg	250 mg/kg
**Males**	Brain	0.6 ± 0.02	0.6 ± 0.02	0.6 ± 0.03	0.6 ± 0.02	0.6 ± 0.02
	Heart	0.3 ± 0.02	0.3 ± 0.02	0.3 ± 0.03	0.3 ± 0.01	0.3 ± 0.01
	Lungs	0.6 ± 0.05	0.6 ± 0.02	0.6 ± 0.05	0.6 ± 0.03	0.6 ± 0.06

	Liver	3.1 ± 0.21	3.2 ± 0.06	3.4 ± 0.10	3.0 ± 0.14	3.0 ± 0.07
	Kidneys	0.6 ± 0.03	0.6 ± 0.02	0.6 ± 0.01	0.6 ± 0.01	0.6 ± 0.03
	Adrenal glands	0.02 ± 0.002	0.02 ± 0.003	0.02 ± 0.001	0.02 ± 0.002	0.02 ± 0.002
	Epididymides	0.3 ± 0.01	0.3 ± 0.01	0.3 ± 0.02	0.3 ± 0.02	0.3 ± 0.02
	Testes	0.9 ± 0.04	0.9 ± 0.03	0.9 ± 0.10	0.8 ± 0.08	0.9 ± 0.07
	Thymus	0.1 ± 0.01	0.1 ± 0.02	0.1 ± 0.01	0.1 ± 0.01	0.1 ± 0.01
	Thymus cellularity (×10^6)	36.8 ± 3.7	35.4 ± 3.7	33.4 ± 4.2	37.1 ± 3.7	39.1 ± 2.4
	Spleen	0.5 ± 0.04	0.4 ± 0.02	0.5 ± 0.07	0.4 ± 0.04	0.5 ± 0.03
	Spleen cellularity (×10^6)	101.5 ± 6.2	103.7 ± 4.3	102.9 ± 7.1	104.5 ± 5.2	106.7 ± 6.3
**Females**	Brain	0.8 ± 0.03	0.8 ± 0.01	0.8 ± 0.02	0.8 ± 0.06	0.8 ± 0.02
	Heart	0.4 ± 0.005	0.4 ± 0.01	0.3 ± 0.03	0.3 ± 0.02	0.3 ± 0.01
	Lungs	0.7 ± 0.03	0.7 ± 0.10	0.6 ± 0.02	0.7 ± 0.06	0.7 ± 0.02
	Liver	3.6 ± 0.21	3.7 ± 0.06	3.5 ± 0.09	3.6 ± 0.20	3.6 ± 0.22
	Kidneys	0.6 ± 0.01	0.6 ± 0.02	0.6 ± 0.02	0.6 ± 0.01	0.6 ± 0.05
	Adrenal glands	0.03 ± 0.002	0.02 ± 0.003	0.03 ± 0.003	0.03 ± 0.003	0.02 ± 0.003
	Ovaries	0.06 ± 0.01	0.06 ± 0.01	0.06 ± 0.01	0.04 ± 0.003	0.05 ± 0.01
	Thymus	0.2 ± 0.02	0.2 ± 0.01	0.2 ± 0.004	0.2 ± 0.02	0.2 ± 0.02
	Thymus cellularity (×10^6)	37.6 ± 4.2	35.4 ± 5.1	34.8 ± 2.6	34.3 ± 4.7	36.5 ± 3.9
	Spleen	0.5 ± 0.01	0.5 ± 0.02	0.5 ± 0.05	0.6 ± 0.02	0.4 ± 0.05
	Spleen cellularity (×10^6)	105.4 ± 3.8	104.3 ± 5.1	105.9 ± 5.1	103.7 ± 5.6	104.8 ± 6.3

Values are presented as mean ± standard error of the mean (M ± m). Relative organ weights are expressed as percentages of body weight. No statistically significant differences were observed between the treatment groups and the control groups ($p > 0.05$).
